# A Molecular Networking Strategy: High-Throughput Screening and Chemical Analysis of Brazilian Cerrado Plant Extracts against Cancer Cells

**DOI:** 10.3390/cells10030691

**Published:** 2021-03-20

**Authors:** Patrícia C. Cortelo, Daniel P. Demarque, Renata G. Dusi, Lorena C. Albernaz, Raimundo Braz-Filho, Ekaterina I. Goncharova, Heidi R. Bokesch, Kirk R. Gustafson, John A. Beutler, Laila S. Espindola

**Affiliations:** 1 Laboratório de Farmacognosia, Universidade de Brasília, Campus Universitário Darcy Ribeiro, Brasília 70910-900, Brazil; pattycar8@gmail.com (P.C.C.); dpdemarque@gmail.com (D.P.D.); renatadusi@hotmail.com (R.G.D.); lalbernaz@unb.br (L.C.A.); 2 Molecular Targets Program, National Cancer Institute, Frederick, MD 21702, USA; katya.goncharova@nih.gov (E.I.G.); heidibokesch@hotmail.com (H.R.B.); gustafki@mail.nih.gov (K.R.G.); beutlerj@mail.nih.gov (J.A.B.); 3 FAPERJ/Departamento de Química, Universidade Federal Rural do Rio de Janeiro, Seropédica, 23897-035 RJ, Brazil and Laboratório de Ciências Químicas, Universidade Estadual do Norte Fluminense, Campos dos Goytacazes, 28013-600 RJ, Brazil; braz@uenf.br; 4 Advanced Biomedical Computational Science, Frederick National Laboratory for Cancer Research, Frederick, MD 21702, USA

**Keywords:** Brazilian Cerrado biome, plant extracts, cancer cell lines, high-throughput screening, NCI-60 cancer cell lines, molecular networking, mass spectrometry

## Abstract

Plants have historically been a rich source of successful anticancer drugs and chemotherapeutic agents, with research indicating that this trend will continue. In this contribution, we performed high-throughput cytotoxicity screening of 702 extracts from 95 plant species, representing 40 families of the Brazilian Cerrado biome. Activity was investigated against the following cancer cell lines: colon (Colo205 and Km12), renal (A498 and U031), liver (HEP3B and SKHEP), and osteosarcoma (MG63 and MG63.3). Dose-response tests were conducted with 44 of the most active extracts, with 22 demonstrating IC_50_ values ranging from <1.3 to 20 µg/mL. A molecular networking strategy was formulated using the Global Natural Product Social Molecular Networking (GNPS) platform to visualize, analyze, and annotate the compounds present in 17 extracts active against NCI-60 cell lines. Significant cytotoxic activity was found for *Salacia crassifolia*, *Salacia elliptica*, *Simarouba versicolor*, *Diospyros hispida*, *Schinus terebinthifolia*, *Casearia sylvestris* var. *lingua*, *Magonia pubescens*, and *Rapanea guianensis*. Molecular networking resulted in the annotation of 27 compounds. This strategy provided an initial overview of a complex and diverse natural product data set, yielded a large amount of chemical information, identified patterns and known compounds, and assisted in defining priorities for further studies.

## 1. Introduction

Natural product drug discovery has relied heavily on terrestrial plants, particularly in the anticancer field, with numerous agents of therapeutic importance. According to Newman and Cragg 2020, 41% of approved antitumor drugs are inspired by natural products; thus, they can be an important source of new structures and can be exploited with synthetic chemistry or synthetic biology [1]. Vinblastine, vincristine, etoposide, and paclitaxel are examples of plant-derived chemotherapeutics currently used in cancer treatment. However, poor solubility in aqueous media, together with significant toxic side effects of many current therapeutics, and limited clinical efficacy, warrant further research to discover novel agents with improved characteristics. Analogues or prodrugs of these agents as well as antibody-drug conjugates have been synthesized to increase aqueous solubility and specifically target tumors with reduced off-target effects. The discovery of biologically active structures from natural products remains important to identify potential drug development candidates [1].

**Figure 1:**
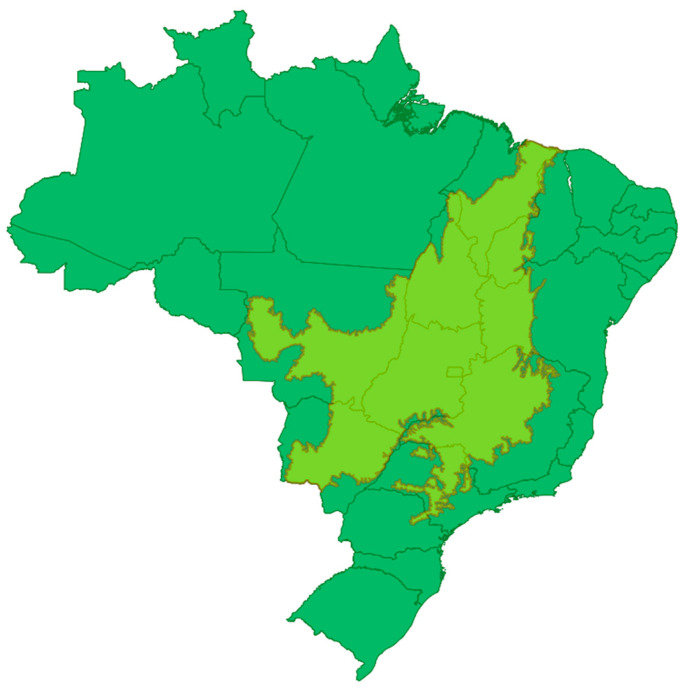
Cerrado delimitation area: light green area in Brazil map. Image from Laboratório de Farmacognosia/UnB adapted from templates available at Project MapBiomas (Project MapBiomas–Collection 5 of Brazilian Land Cover & Use Map Series, accessed on 26 February 2021 through the link: https://mapbiomas.org/mapas-de-referencia?cama_set_language=pt-BR).

The Cerrado is the second largest biome in Brazil, occupying 21% of the total land area (Figure 1). It is considered a global hotspot for conservation of biodiversity due to its species richness and rapid loss of natural habitats. More than 50% of the original forest cover has been converted for new uses, mainly croplands and pasturelands. Unfortunately, soil degradation and habitat fragmentation result in pressure on the last large remnants of native vegetation. Besides its biological importance, the Cerrado plays a crucial role in producing and conserving water resources that contribute to three important hydrological basins in Brazil. This contribution is related to the Cerrado soils that are old clayey Oxisols favoring rainwater infiltration. Cerrado vegetation also represents a significant carbon stock in above and below ground biomass. The great diversity of habitats is supported by factors such as distance from watercourses, depth of soil and groundwater, soil composition, frequency of fires, and regional climate. The high number of endemic species and overall species richness make the Cerrado a rich source of potential natural products discoveries and studies [2].

Metabolomic and chemometric tools have played an important role in the discovery of natural bioactive compounds by the exploration and correlation of metabolic profiles, or as dereplication strategies to avoid re-isolation of known compounds [3,4]. One of the most widely used techniques is high-resolution mass spectrometry (HRMS) which can collect data from untargeted (qualitative detection of all measurable metabolites) or targeted (quantification of a specific group of compounds) analyses [4]. In molecular networking, experimental data are analyzed using algorithms to group compounds of the same class according to their mass fragmentation similarities. Comparison with databases (dereplication) in public or in-house libraries enables the annotation of known metabolites. This step can provide an initial overview of vast data sets and helps to guide research priorities [5]. One of the platforms used to perform molecular networking is Global Natural Products Social Molecular Networking (GNPS). This platform consists of an open-access web-based platform available for mass spectrometry raw data analysis (organization, processing and annotation of fragments), storage, and sharing of both raw and processed data, which enables molecular network construction applied to natural products and peptides [3,4].

In the current study, a panel of eight cancer cell lines was utilized, followed by NCI-60 screening and HRMS/MS molecular networking, to evaluate extracts from the Brazilian Cerrado for their cytotoxic activity against tumor cells and explore the associated chemical diversity. The eight cancer cell lines were chosen to include two each from the NCI-60 kidney and colon cancer panels, which respond with different sensitivity to many drugs [6,7], and two each of osteosarcoma [8] and liver cancer cell lines [9] which have rarely been tested with natural products.

Extracts housed at the Laboratório de Farmacognosia (Universidade de Brasília) were evaluated by high-throughput cell screening (HTS) [6,7,8,9] and an untargeted mass spectrometry-based molecular networking strategy using the GNPS platform [3]. The NCI-60 cell line screen is a tool employed early in cancer drug discovery and development utilizing a panel of 60 human tumor cell lines representative of a variety of tumor types [10,11,12]. The assay is used to assess extract or compound activity, and it also provides valuable information regarding potential mechanisms of drug action based on the sensitivity profile of the 60 individual cell lines.

## 2. Materials and Methods

### 2.1. Plant Extracts

Plant material was collected in the Cerrado biome near Brasilia, DF, Brazil. Figure 1 illustrates the original area of Cerrado in Brazil. The Cerrado climate is highly seasonal, marked with dry (May–September) and rainy (October–April) seasons. The different species were collected mainly in the dry season (74%), in which the additional stress stimulates Cerrado plants to produce higher metabolite diversity. All individuals were adults and were collected on oxisol or inceptisol soils (Table S1). 

These two soil types cover about 85% of the Federal District in the Cerrado biome’s central region. Oxisols are highly weathered and deep soils, with low fertility and a thick, leached horizon of hydrous iron- and aluminum-oxide clays. While oxisols are considered old soils, inceptisols are soils in the early stages of development. This type of soil is less thick and occurs in wavy relief [13,14]. 

All species were collected by the botanist Professor José Elias de Paula (*in memoriam*) and voucher specimens were deposited in the Universidade de Brasília (UB/UnB) Herbarium. Plant organs were separated, dried, then pulverized and extracted by maceration with solvents of different polarities. The extract solutions were filtered and concentrated with a rotary evaporator at 35 °C to yield crude extracts, which were stored at −20 °C (Table S1). The Laboratório de Farmacognosia of the Universidade de Brasília is authorized by national authorities to access Brazilian biodiversity under a license granted by CGEN/IBAMA No. 06/2012–Process No. 02000.002272/2006-73. Extract transportation to the United States was authorized under special CGEN/IBAMA exportation license No. 14BR014583/DF.

### 2.2. Antiproliferative Bioassays 

The XTT endpoint assay developed by the Assay Development and Screening Section of the Molecular Targets Program was used for the initial antiproliferative bioassays. The tests were performed as described previously [15]. Extracts were dissolved in DMSO and the 702 extracts were screened at a single concentration of 10 µg/mL against 8 cancer cell lines: Colo205 and Km12 (colon), A498 and U031 (renal), HEP3B and SKHEP (liver), and MG63 and MG63.3 (osteosarcoma). A hit was defined as >50 percent inhibition of cell growth. Next, the 44 active extracts were tested in a 5-point dose response format in colon, renal, and osteosarcoma cell lines (Table S2), and the IC_50_ values determined in duplicate for the 22 most active extracts by regression analysis. Based on these results, 17 extracts were selected for the NCI-60 cell assay. This screen utilizes 60 different human tumor cell lines, representing leukemia, melanoma, and cancers of the lung, colon, brain, ovary, breast, prostate, and kidney. Three endpoints were calculated: (1) GI_50_, the concentration causing 50% growth inhibition; (2) TGI, the concentration causing total growth inhibition, and (3) LC_50_, the concentration causing 50% lethality of the starting cells [10,12,16].

### 2.3. HRESIMS Data Acquisition

Crude extracts (25 mg) were dissolved in methanol and loaded on SPE C18 cartridges (Strata C18-Phenomenex–500 mg). Each cartridge was then eluted with 3 mL methanol. The resulting fractions were dried in a stream of nitrogen, weighed, and an aliquot resuspended in methanol at 1 mg/mL. The solutions were filtered (0.22-µm filter, Millipore) and a 10-µL aliquot injected into a UHPLC-MS/MS system (Bruker Daltonics, Elute pump UHPLC, Elute autosampler UHPLC, Elute DAD) with an ESI-qTOF mass spectrometer (Compact QTOF, Bruker Daltonics). The diode array detector (DAD) was programmed to scan from 200 to 700 nm. The mobile phase used was MeOH/H_2_O, in an exploratory gradient ranging from 5 to 98% B in A (B: MeOH/0.1% acetic acid and A: H_2_O ultrapure/0.1% acetic acid) for 13 min, the column was stabilized with 5% B for 2 min at the start and the end of the run, with a flow rate of 0.5 mL/min. The column used was an Intensity Solo 1.8 C18-2 (Bruker Daltonics), dimensions of 100 × 2.1 mm, with 1.8-μm particles and 100-Å pore, protected with a pre-column of the same material. The column temperature was set at 40 °C. The samples were maintained at 20 °C in the autosampler. The data were acquired by Data Analysis software v.4.4 (Bruker Daltonics). The MS adjustment parameters were: desolvation gas (nitrogen) flow of 9 L/min, at a pressure of 4 bar in the collision cell. The capillary voltage was 4.5 kV and the source temperature was 200 °C. The mass range was *m/z* 50–1000 for both positive and negative ionization modes. The cone voltage and collision energy were 4 eV and 7 eV, respectively, for quadrupole and the collision cell. The waiting time was automatically set to 5 µs. The analysis was performed in Auto_MS.

### 2.4. Data Processing and Molecular Networking Construction

UPLC-MS/MS technology was used to analyze the 17 active extracts assayed in the NCI-60 panel test, in the positive ion mode according to the above conditions. ESI-MS/MS data were converted to .mzXML format using the Bruker data analysis software (v.4.3). The MZmine2 software [17,18] was used for data processing: peak detection and normalization, deconvolution, deisotoping, noise filtering, and gap-filling were set as follows-mass detection at the centroid and chromatogram building used a minimum time span of 0.05 min, a minimum height of 10000, and *m/z* tolerance of 10 ppm. The ADAP (Automated Data Analysis Pipeline) deconvolution algorithm [19] was used (chromatographic threshold = 1%, peak duration range = 0.05–2 min, minimum relative height = 1%, minimum absolute height = 5000, minimum ratio of peak top/edge = 2). Chromatograms were deisotoped using the isotopic peak grouper algorithm with an *m/z* tolerance of 10 ppm and an RT tolerance of 2 min. Peak alignment was performed using the Join aligner method as follows: *m/z* tolerance at 10 ppm, absolute retention time (RT) tolerance 2 min, absolute RT tolerance after correction of 1 min, and a threshold value of 1. The peak list was subsequently gap-filled with the peak finder module-intensity tolerance at 5%, *m/z* tolerance at 10 ppm, and absolute RT tolerance of 2 min. Molecular networking analysis was performed with an exported MZmine2 .mgf table file into the global natural products social molecular networking (GNPS) online platform using the feature-based molecular networking (FBMN) [20] mode and the following parameters to build networks: clustering the data- precursor ion tolerance was set at 0.1 Da; fragment mass tolerance at 0.05 Da; cosine score > 0.7; minimum matched fragment ions at 6, and network topK at 10. Finally, the resulting molecular networking was visualized using the Cytoscape software (v.3.7.2) [21]. Annotation was made using the Dictionary of Natural Products (DNP) [22] and Metlin (XCMSonline) databases [23,24].

## 3. Results

A total of 702 Brazilian Cerrado plant extracts from 95 different species, representing 40 families (Table S1), were submitted to high-throughput screening (10 μg/mL) against 8 cancer cell lines. This initial screening against colon (Colo205 and Km12), renal (A498 and U031), liver (HEP3B and SKHEP), and osteosarcoma (MG63 and MG63.3) cells resulted in the selection of 44 active extracts (6.3%) for dose-response testing. None of the initial extracts inhibited hepatic cancer cell growth. 

**Figure 2:**
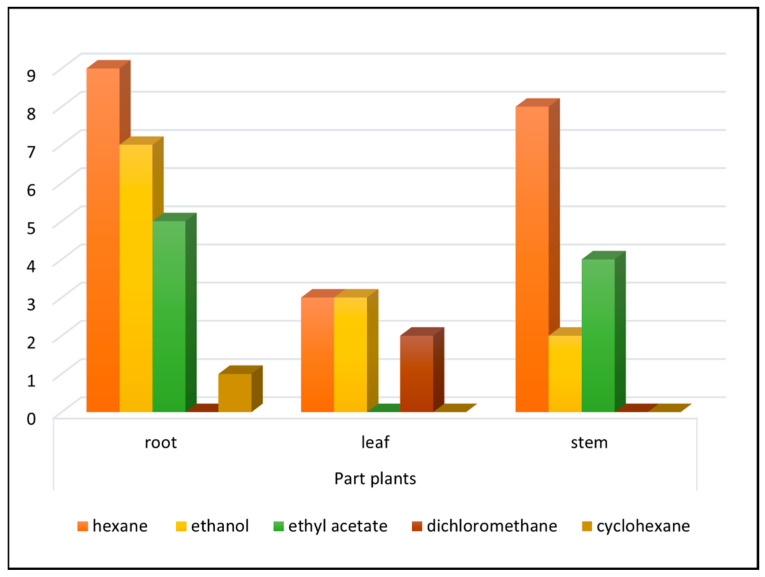
Plant parts and extraction solvents of the 44 active extracts selected for dose-response determination.

The 44 extracts selected were from different plant parts: root wood and root bark (22 extracts, 50%), stem wood and stem bark (14 extracts, 32%), leaf (7 extracts, 16%), and rhizome (1 extract, 2%). Extracts were prepared using different solvents: hexane, ethanol, ethyl acetate, dichloromethane or cyclohexane (Figure 2). Of the 702 extracts, 207 (31%) were from the root and 270 (39%) were from the stem. While 44 extracts (6.3% of the extract bank) were active, a higher percentage (10%) of the bank’s root extracts were cytotoxic (21/207 root extracts), an indication that the root may constitute a chemically rich organ in Cerrado plants, possessing defensive metabolites with inhibitory properties, including cancer cell line growth inhibition. In previous screening studies involving the same extract library against protozoa and fungi, the root and stem extracts proved the most promising, primarily root and stem bark extracts [25].

### 3.1. High-Throughput Screening 

Dose-response curves (of 44 extracts tested) were generated and IC_50_ values of the 22 most active extracts ranged from 1.3 to 20 μg/mL against 6 cell lines (Table 1). The Z-factors for plates used in initial screening and secondary dose response testing are described in Table S2. The hexane extract of *Salacia crassifolia* (Celastraceae) root wood was cytotoxic against the KM12 colon cancer cell line (IC_50_ 1.7 μg/mL) and demonstrated no activity against the Colo205 colon cancer cells. Similarly, it was active against the A498 renal cancer cell line (IC_50_ 1.6 μg/mL) but showed no activity against renal cancer U031 cells. The *Salacia elliptica* root wood EtOAc extract was active against all 6 cell lines (IC_50_ 2.0 to 7.1 μg/mL), with the highest activity against U031 of the extracts tested. 

The ethanol extract of *Simarouba versicolor* (Simaroubaceae) root bark was the most cytotoxic extract against both osteosarcoma cell lines, MG63 and MG63.3, with IC_50_ values <1.3 μg/mL. The *Schinus terebinthifolia* (Anacardiaceae) leaf dichloromethane extract was also active against MG63 (IC_50_ 3.4 μg/mL). *Casearia sylvestris* var. *lingua* (Salicaceae) stem wood hexane extract was cytotoxic against the 6 cell lines (IC_50_ 2.7 to 5.4 μg/mL). The *Enterolobium gummiferum* (Fabaceae) root wood ethanol extract was active against both renal cell lines, with higher activity against A498 cells (IC_50_ < 1.3 μg/mL). For this same species, the stem bark hexane extract was selective for colon cancer cells Colo205 and KM12, with IC_50_ 2.2 and 4.9 μg/mL, respectively. Two of the *Rapanea guianensis* (Primulaceae) extracts (stem wood hexane and root wood ethanol) showed activity against the colon and renal cells tested, with IC_50_ values between 2.7 and 5.3 μg/mL (Table 1).

Based on the selective cytotoxicity results, we submitted the 17 most active extracts to NCI-60 cell one-dose and then five-dose screening (Figures S5–S55). The strongest activity was found for the *S. crassifolia* root wood hexane extract (BR 640; N192803), presenting a mean GI_50_ 0.3 μg/mL for all 60 cell lines, with the HCT-15 colon cancer cell line the most sensitive (GI_50_ 0.1 μg/mL) (Table 1). Another *Salacia* species extract, *S. elliptica* root wood EtOAc (BR 652; N192805), demonstrated a mean GI_50_ 2.0 μg/mL and was most potent against the leukemia MOLT-4 cell line (GI_50_ 0.7 μg/mL) (Table 1). *Simarouba versicolor* root bark ethanol (BR 254; N192829) and *Diospyros hispida* (Ebenaceae) root (wood and bark) EtOAc (BR 501; N192799) extracts, displayed similar results against the non-small cell lung NCI-H522 cell line (GI_50_ 0.7 μg/mL) (Table 1). This cell line was also sensitive to the *S. terebinthifolia* leaf dichloromethane (BR 436; N192835) and *C. sylvestris* var. *lingua* stem wood hexane (BR 177; N192825) extracts, with GI_50_ values of 0.9 μg/mL and 2.5 μg/mL, respectively (Table 1). Both the *Magonia pubescens* (Sapindaceae) (BR 204; N192797) and *Rapanea guianensis* (BR 627; N192801) root wood ethanol extracts were primarily active against the leukemia SR cell line, with respective GI_50_ values of 0.3 μg/mL and 2.4 μg/mL (Table 1). 

### 3.2. Molecular Networking

Analysis of the 17 most active extracts using a MS/MS dereplication approach resulted in the construction of the molecular network. The LC-MS/MS data were converted to .mzXML files, preprocessed using the MZmine2 [17,18] software in order to align and detect the most relevant information, together with the most intense chromatographic peaks. The resulting .cvs table was uploaded to the GNPS platform and organized according to fragmentation pattern profile similarities. This process improved data visualization by generating a network with clusters corresponding to different compound classes. Clusters are groups of nodes (plotted here as pie charts representing compound percentages in each of the 17 extracts) and the connecting lines constitute edges (cosine values that represent related patterns between nodes). In this study, the total molecular networking (MN), visualized in the Supplementary Material as Figure S1, consists of 47 clusters and 279 selfloops. It was possible to annotate 25 compounds. An additional 2 compounds were annotated using public databases-Dictionary of Natural Products (DNP) [22] and Metlin [23,24]. The annotated compounds, 27 in total, are listed in Table 2. 

**Figure 3:**
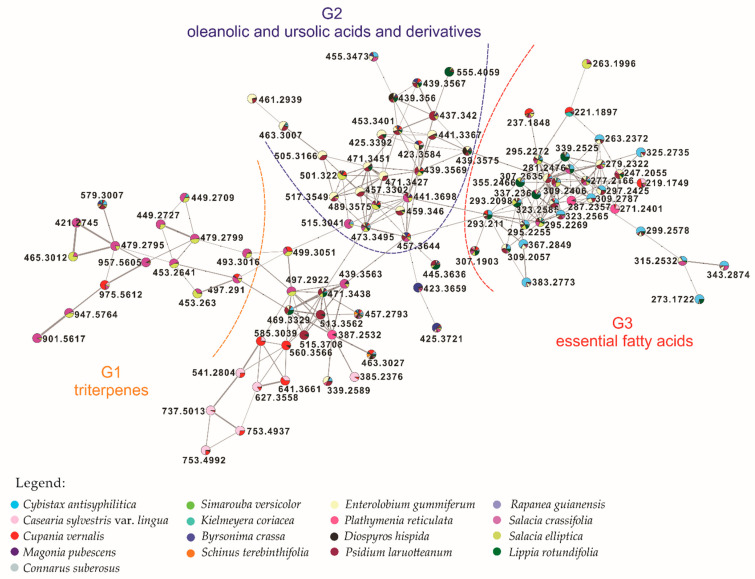
Main cluster-3 sub-networks: G1 triterpenes; G2 oleanolic and ursolic acids and derivatives; G3 essential fatty acids–linoleic and linolenic acids and derivatives.

To better visualize and analyze the data collected from the 17 extracts selected, we performed a molecular networking workflow associated with high-throughput cancer screening. Detailed inspection of one main cluster (Figure 3) showed that the metabolites were grouped into three sub-networks (G): G1-triterpenes; G2-oleanolic/ursolic acids and derivatives; and G3-essential fatty acids-linoleic and linolenic acids and derivatives interconnected by common biosynthetic routes (biosynthesis of fatty acids, unsaturated fatty acids, elongation and reduction of the fatty acid chain). The metabolites here are present in all 17 plant extracts, except for triterpenes that are mainly detected in *Salacia* species.

In the G2 sub-network, oleanolic acid [M + H − H_2_O]^+^ *m/z* 439.3567; 3α-cyclopenta [α] chrysene-3α-carboxylic acid [M + H – H_2_O]^+^ *m/z* 441.3367, and platanic acid [M + H]^+^ *m/z* 459.346 were annotated. Oleanolic and ursolic acids are pentacyclic triterpene isomers, differing in the methyl group position in the E ring [26]. Both acids are used in Chinese medicine as hepatoprotectives, with oleanolic acid also possessing anti-tumor activity [27,28], and ursolic acid possessing anti-HIV, anti-inflammatory, antiulcer, hypolipidemic, and antiatherosclerotic activities [29]. Platanic acid was isolated primarily from *Syzygium claviflorum* leaves that exhibit anti-HIV activity, with studies of its derivatives suggesting antitumoral activity [30,31,32]. We observed that the G3 sub-network contains a conjugated linoleic acid [M + H]^+^ *m/z* 281.2476 and derivatives: ethyl-linoleic ester [M + H]^+^ *m/z* 309.2787; ethyl-linolenic ester [M + H]^+^ *m/z* 307.2635; 9-oxo-10E,12Z-octadecadienoic acid [M + H]^+^ *m/z* 295.2269; 13-keto-9Z,11E-octadecadienoic acid [M + H]^+^ *m/z* 295.2272; stearidonic acid [M + H]^+^ *m/z* 277.2166; 9(10)-epoxy-12Z-octadecenoic acid [M + H − H_2_O]^+^ *m/z* 279.2322, and 9S,13R-12-oxophytodienoic acid [M + H]^+^ *m/z* 293.2098 (Table 2). These essential fatty acids are produced exclusively by plants [33]. Recent research suggests that a diet enriched with linoleic acid is related to a reduction in the incidence of cancer [32,33]. Linolenic and linoleic acids may act via different mechanisms to inhibit the development of some types of cancer, especially breast cancer [33,34,35]. The G1 sub-network contains: pristimerin [M + H]^+^ m/z 465.3012; tingenone [M + H]^+^ m/z 421.2745; 20-oxo-20,21-seco-tingen-21-oic acid [M + H]^+^ m/z 453.263, and other unidentified derivatives in the database. The common fragment (*m/z* 201.0609) suggests that these unidentified derivatives resulted from fragmentation involving carbocation formation, followed by displacement of a methyl and pericyclic rearrangement. They are predominantly present in *S. crassifolia* and *S. elliptica*, with reported activity against cancer cell lines [36,37].

In minor clusters (Figure S3, purple highlighted circle), it was possible to annotate a catechin derivative cluster: catechin gallate [M + H]^+^ *m/z* 443.0972 and epigallocatechin gallate [M + H]^+^ *m/z* 459.0944. These catechin-derived flavonoids have been studied for their properties of inhibiting pancreatic cancer with promising results [38,39]. In another cluster (Figure S3, orange highlighted circle), luteolin 3′,4′-di-O-beta-D-glucopyranoside, a glycosylated flavone with [M + NH_4_]^+^ *m/z* 628.1959 was annotated [40]. Although it was not possible to identify other compounds here, the fragment ions obtained showed that they were glycosylated flavones probably derived from the aforementioned annotated flavone.

## 4. Discussion

Cancer remains a complex and challenging disease that is often fatal [41]. Natural products constitute a high percentage of chemotherapeutic and chemoprevention drugs, which may be used in isolation or as part of a combined cancer treatment strategy [41]. The cytotoxicity data in this study supports the Brazilian Cerrado biome as a rich source of potential chemotherapeutic agents. 

Initial screening of 702 plant extracts against 8 different cell lines resulted in the selection of 44 active extracts for dose-response determination. This data, together with the IC_50_ value determination of 22 of these extracts, led to NCI-60 cell screening of the 17 most active extracts, corresponding to 2.4% of the Brazilian Cerrado biome extract bank. This test has been used for the last three decades to select chemicals and natural product extracts with the ability to inhibit the growth of, or kill, cancer cells [10,11]. 

Some of these 17 extracts were previously investigated against other biological targets such as: (i) protozoa-*S. terebinthifolia* and *D. hispida* (*Plasmodium falciparum*) [42], *C. sylvestris* var. *lingua* (*Trypanosoma cruzi* and *Leishmania donovani*) [43], C. v*ernalis* (*L. donovani*) [43] and *C. suberosus* (*L.* (*L.*) *amazonensis*) [25]; and (ii) yeasts and dermatophytes - *D. hispida* and *C. suberosus* [25,42].

*S. crassifolia* and *S. elliptica* root wood extracts exhibited the strongest cytotoxicity, previously reported for the *Salacia* genus [36]. Another root extract, *Simarouba versicolor* root bark ethanol, presented strong activity against both osteosarcoma cell lines (Table 1). A previous study involving a hexane extract of the same *S. versicolor* organ led to the isolation of glaucarubinone, a cytotoxic compound [44].

From molecular networking analysis, *S. crassifolia* and *S. elliptica* extracts, as shown in the attached clusters formed, represent a different metabolic profile from the other plants, with metabolites not reported in the database (Figure S3, green highlighted circle). Furthermore, they exhibited the most potent activity against cancer cell lines in the high-throughput screening. *C. sylvestris* var. *lingua* and *Cupania vernalis* (Sapindaceae) extracts generated clusters which indicated similar metabolic profiles despite belonging to different plant families. However, *C. sylvestris* var. *lingua* exhibited more potent activity against 6 cancer cell lines (IC_50_ value determination) and in the NCI-60 screen. The latter also showed higher activity against SF-295, HCT-8, MDA-MB-435 and LH-60 cell lines with IC_50_ values ranging from 0.5 and 2.1 μg/mL when compared to *C. vernalis* [44] 

An interesting result was noted regarding *S. versicolor* and *D. hispida*. Both species showed considerable activity, but did not form an exclusive cluster (noted in Figure S3). However, a few compounds were more intense (peak area intensity) in these plants than in the other species. A similar observation was made for *Rapanea guianensis* and *Connarus suberosus* (Connaraceae), which have comparable metabolite profiles. These observations pave the way for future studies to fractionate the extract and determine if a single active compound or combination of compounds results in the observed activity. For example, in this study, *C. sylvestris* var. *lingua* and *C. vernalis* are particularly interesting in that they presented high activity in the NCI-60 screen and showed 2 interesting cluster formations (Figures S2 and S3, red highlighted circle), with no compounds annotated. Further investigations involving isolation and characterization are necessary to elucidate the active compounds.

In conclusion, molecular networking provides a useful addition to the decision-making process when presented with of a large number of positive results. This streamlined strategy can not only economize time and resources by avoiding the isolation of known compounds, it also guides the isolation of unreported active compounds. Limitations of this analytical technique include the lack of compounds in databases and the possible presence of minor compounds that could account for the activity. Nevertheless, we have shown that a large amount of chemical information can be extracted using this strategy to provide an overview of chemical diversity and biological potential. We obtained metabolite information that did not match any compound described in the databases, possibly responsible for the cancer activity results. Finally, the chemical diversity from Brazilian Cerrado plants against cancer cell lines supports conservation of this biome hotspot to safeguard its valuable and unique biodiversity.

## Figures and Tables

**Table 1:** Brazilian Cerrado plant extract activity against cancer cell lines (IC_50_ μg/mL) and NCI-60 sensitivity (potency in μg/mL).

Plant Species/Plant Organ (Solvent)*	Codes BR/NCI60	IC_50_ (μg/mL)						NCI-60 Sensitivity Potency (μg/mL)		
		Colon		Renal		Osteosarcoma				
		Colo205	KM12	A498	U031	MG63	MG 63.3	Mean GI_50_/Most Sensitive Cell Line	Mean TGI	Mean LC_50_
*Peschiera affinis* SB (h)	BR 075	17.8	19.1	>20	>20	>20	>20	-	-	-
*Cybistax antisyphilitica* SB (h)	BR 125 N192795	-	-	-	-	-	-	85	>100	>100
*Tabebuia caraiba* L (h)	BR 139	>20	18.9	>20	>20	>20	>20	-	-	-
*Casearia sylvestris* var*. lingua* SW (h)	BR 177 N192825	4.6	4.5	3.6	2.7	3.9	5.7	11/2.5 NCI-H522	28	76
*Cupania vernalis* L (h)	BR 193 N192827	12.3	10	18.7	7.6	13.8	15	43	140	490
*Magonia pubescens* R (e)	BR 197	4.2	7.2	3.6	3.9	14.5	14.5	-	-	-
*Magonia pubescens* RW (e)	BR 204 N192797	3.5	5.5	2.9	3.8	15.4	15.6	15/0.3SR	34	81
*Simarouba versicolor* RB (e)	BR 254 N192829	6.8	>20	3.7	5	<1.3	<1.3	4.9/0.7 NCI-H522	29	620
*Simaba suffruticosa* L (a)	BR 261	16.8	1.6	>20	17.6	2.2	4.9	-	-	-
*Kielmeyera coriacea* SW (h)	BR 331 N192831	11.9	4.9	15.9	14.9	>20	>20	56	210	600
*Byrsonima crassa* RB (h)	BR 411 N192833	15	15	>20	>20	>20	>20	220	530	910
*Schinus terebinthifolia* L (d)	BR 436 N192835	12.4	6.9	16.2	7.8	3.4	14.8	100/0.9 NCI-H522	87	360
*Enterolobium gummiferum* RW (e)	BR 467	10.5	10.9	<1.3	4.6	12.6	14.9	-	-	-
*Enterolobium gummiferum* SB (h)	BR 469 N192837	2.2	4.9	>20	>20	>20	>20	180	710	980
*Plathymenia reticulata* RW (h)	BR 489 N192839	8.9	6.7	12.4	4.9	>20	>20	60	330	680
*Diospyros hispida* R (a)	BR 501 N192799	-	-	-	-	-	-	7/0.7 NCI-H522	30	81
*Maprounea guianensis* RB (a)	BR 536	8.1	9.9	6.7	>20	9.6	8.5	-	-	-
*Psidium laruotteanum* SB (h)	BR 549 N192841	-	-	-	-	-	-	87	340	830
*Andira humilis* SB (e)	BR 587	>20	18.6	>20	>20	>20	>20	-	-	-
*Rapanea guianensis* SW (h)	BR 624	5.3	3.2	4.3	4.4	17.8	14.9	-	-	-
*Rapanea guianensis* RW (e)	BR 627 N192801	3.5	4.0	2.7	3.7	14.6	14	16/2.4 SR	361	81
*Salacia crassifolia* RW (h)	BR 640 N192803	>20	1.7	1.6	>20	3.7	5.7	0.3/0.1 HCT-15	1.0	5.0
*Salacia elliptica* RW (a)	BR 652 N192805	3.6	3.4	3.5	2.0	5.1	7.1	2.0/0.7 MOLT-4	5.1	20
*Lippia rotundifolia* SW (a)	BR 660 N192843	12.3	9.9	>20	>20	>20	>20	-	-	-
*Connarus suberosus* RW (a)	BR 693 N192845	5.7	4.5	4.7	7.7	17.6	19	44	200	830

IC_50_: inhibitory concentration (50%) *Plant organ: L–leaf; SB–stem bark; SW–stem wood; R–root (wood + bark); RW–root wood; RB–root bark; Rz-rhizome. *Extraction solvent: h–hexane; e–ethanol; a–ethyl acetate. GI_50_: the concentration causing 50% growth inhibition; TGI: the concentration causing total growth inhibition; LC_50_: the concentration causing 50% lethality of the starting cells.

**Table 2:** Compounds annotated by molecular networking of 17 Cerrado plant crude extracts.

Compound Name	Molecular Formula	*m/z*	Rt (min)
oleanolic acid*	C_30_H_48_O_3_	439.3567	9.7
ursolic acid*	C_30_H_48_O_3_	439.3567	9.7
3α-cyclopenta[α]chrysene-3α-carboxylic acid	C_29_H_46_O_4_	441.3367	7.5
platanic acid	C_29_H_46_O_4_	459.346	7.6
linoleic acid	C_18_H_32_O_2_	281.2476	8.4
linoleic acid ethyl ester	C_20_H_36_O_3_	309.2787	10.0
linolenic acid ethyl ester	C_20_H_34_O_2_	307.2635	9.6
13-keto-9Z,11E-octadecadienoic acid	C_18_H_30_O_3_	295.2272	8.4
9-oxo-10E,12Z-octadecadienoic acid	C_18_H_30_O_3_	295.2269	8.5
stearidonic acid	C_18_H_28_O_2_	277.2166	8.3
9(10)-epoxy-12Z-octadecenoic acid	C_18_H_32_O_3_	279.2322	8.1
9S,13R-12-oxophytodienoic acid	C_18_H_28_O_3_	293.2098	5.3
pristimerin	C_30_H_40_O_4_	465.3012	10.3
tingenone	C_28_H_36_O_3_	421.2745	8.6
20-oxo-20,21-seco-tingen-21-oic acid	C_28_H_36_O_5_	453.263	5.9
(-)-catechin gallate	C_22_H_18_O_10_	443.0972	3.7
epigallocatechin gallate	C_22_H_17_O_11_	459.0944	3.2
luteolin 3′,4′-di-O-beta-D-glucopyranoside	C_27_H_29_O_16_	628.1959	10.7
13-docosenamide	C_22_H_43_NO_3_	338.342	11.7
9-octadecenamide	C_18_H_35_NO	282.2793	10.1
N-phenyl-1-naphthylamine	C_16_H_13_N	220.1124	8.2
(2R,3S,4S,5R,6S)-2-[[(2S,3R,4R)-3,4-dihydroxy-4-(hydroxymetil)oxolan-2-yl]oxymetil]-6-(3,4,5-trimethoxyphenoxy)oxane-3,4,5-triol	C_20_H_30_O_13_	496.1988	3.0
(3R,5R,6R,7S,9S,10R,13R,17R)-17-((R)-5-ethoxy-5-oxypentan-2-yl)-10,13-dimethylhexadecahydro-1H-cyclopenta[a]phenanthreno-3,6,7-triyl triacetate	C_32_H_50_O	585.3039	9.0
1-linoleoilglycerol	C_16_H_36_O_3_	355.282	7.9
hesperidine	C_28_H_34_O_15_	611.498	4.1
palmitamide	C_20_H_41_NO	256.2639	10.0
4-(2,6,6-trimethyl-4-oxo-2-ciclohexen-1-yl)-2-butanyl beta-D-glucopyranoside	C_19_H_32_O_7_	373.2192	4.2

* The annotation may change. Rt: retention time.

## Data Availability

The data presented in this study are available on request from the corresponding author.

## Supplementary Materials

The following are available online at https://www.mdpi.com/2073-4409/10/3/691/s1, Table S1. Brazilian Cerrado plant extracts submitted to high-throughput screening in a set of 8 cancer cell lines: colon (Colo205 and Km12), renal (A498 and U031), liver (HEP3B and SKHEP) and osteosarcoma (MG63 and MG63.3). Table S2. Z-factors for plates used in initial screening and secondary dose response testing. Figure S1. Complete molecular networking (MN) of 17 Cerrado plant extracts assayed in NCI-60. Figure S2. Expansion of the network, top portion. Figure S3. Expansion of the network, central portion. Figure S4. Expansion of the network, bottom portion. Figure S5–S55. Results graphs of NCI-60 screening.
