# Supplementary material for: A Molecular Networking Strategy: High-Throughput Screening and Chemical Analysis of Brazilian Cerrado Plant Extracts against Cancer Cells
Source: Cells. 2021 Mar 20;10(3):691. doi: 10.3390/cells10030691 (PMC8004027; doi:10.3390/cells10030691)
Supplement: Supplementary file 1 [file cells-10-00691-s001.pdf]

Article

A molecular networking strategy: High-throughput screening and chemical analysis of Brazilian Cerrado plant extracts against cancer cells

Patrícia C. Cortelo[1], Daniel P. Demarque[1], Renata G. Dusi[1,2], Lorena C. Albernaz[1], Raimundo Braz-Filho[3], Ekaterina I. Goncharova[2,4], Heidi R. Bokesch[2], Kirk R. Gustafson[2], John A. Beutler[2], Laila S. Espindola[1,2,*]

[1] Laboratório de Farmacognosia, Universidade de Brasília, Campus Universitário Darcy Ribeiro, Brasília, 70910-900, Brazil; pattycar8@gmail.com (P.C.C.); dpdemarque@gmail.com (D.P.D); renatadusi@hotmail.com (R.G.D); lalbernaz@unb.br (L.C.A); darvenne@unb.br (L.S.E.)

[2] Molecular Targets Program, National Cancer Institute, Frederick, MD 21702, USA; katya.goncharova@nih.gov (E.I.G.); heidibokesch@hotmail.com (H.R.B.); gustafki@mail.nih.gov (K.R.G.); beutlerj@mail.nih.gov (J.A.B.)

[3] FAPERJ/Departamento de Química, Universidade Federal Rural do Rio de Janeiro, Seropédica, RJ and Laboratório de Ciências Químicas, Universidade Estadual do Norte Fluminense, Campos dos Goytacazes, RJ, Brazil; braz@uenf.br (R.B.F.)

[4] Advanced Biomedical Computational Science, Frederick National Laboratory for Cancer Research, Frederick, MD, 21702, USA; katya.goncharova@nih.gov

* Correspondence: darvenne@unb.br (L.S.E.); + 55 61 31072016

Dedicated to the memory of Professor José Elias de Paula

CONTENTS

Table S1. Brazilian Cerrado plant extracts submitted to high-throughput screening against a set of 8 cancer cell lines: colon (Colo205 and Km12), renal (A498 and U031), liver (HEP3B and SKHEP) and osteosarcoma (MG63 and MG63.3)........ **1**

Table S2. Z-factors for plates used in initial screening and secondary dose response testing……………15

MOLECULAR NETWORKING

Figure S1. Complete molecular networking (MN) of 17 more active Cerrado plants........ **16**

Figure S2. Expansion of the network, top portion........ **17**

Figure S3. Expansion of the network, central portion........ **18**

Figure S4. Expansion of the network, bottom portion........ **19**

RESULTS GRAPHS OF NCI-60 SCREENING S5 to S55

A. *Cybistax antisyphilitica*

Figure S5. Dose response curves of the *Cybistax antisyphilitica* stem bark hexane extract (BR 125/N192795) against 9 cell panels with different susceptibility........ **20**

Figure S6. Mean bar graph of the *Cybistax antisyphilitica* stem bark hexane extract (BR 125/N192795) in the NCI-60 cell five-dose screen. **21**
Figure S7. Composite of the NCI-60 dose response curves for the *Cybistax antisyphilitica* stem bark hexane extract (BR 125/N192795). **22**

B. *Magonia pubescens*
Figure S8. Dose response curves of the *Magonia pubescens* root wood ethanol extract (BR 204/N192797) against 9 cell panels with the highest activity against the leukemia SR cell line. **23**
Figure S9. Mean bar graph of the *Magonia pubescens* root wood ethanol extract (BR 204/N192797) in the NCI-60 cell five-dose screen. **24**
Figure S10. Composite of the NCI-60 dose response curve of the *Magonia pubescens* root wood ethanol extract (BR 204/N192797) with higher activity against the leukemia SR cell line. **25**

C. *Diospyros hispida*
Figure S11. Dose response curves of the *Diospyros hispida* root (wood + bark) ethyl acetate extract (BR 501/N192799) against NCI-60 panels, with the highest activity against the non-small cell lung cancer NCI-H522. .... **26**
Figure S12. Mean bar graph of the *Diospyros hispida* root (wood + bark) ethyl acetate extract (BR 501/N192799) in the NCI-60 cell five-dose screen. **27**
Figure S13. Composite of the NCI-60 dose response curves of the *Diospyros hispida* root (wood + bark) ethyl acetate extract (BR 501/N192799) with higher activity against the non-small cell lung cancer NCI-H522.. **28**

D. *Rapanea guianensis*
Figure S14. Dose response curves of the *Rapanea guianensis* root wood ethanol extract (BR 627/N192801) against NCI-60 panels, with the highest activity against the leukemia SR cell line. **29**
Figure S15. Mean bar graph of the *Rapanea guianensis* root wood ethanol extract (BR 627/N192801) in the NCI-60 cell five-dose screen. **30**
Figure S16. Composite of the NCI-60 dose response curves of the *Rapanea guianensis* root wood ethanol extract (BR 627/N192801) with higher activity against the leukemia SR cell line. **31**

E. *Salacia crassifolia*
Figure S17. Dose response curves of the *Salacia crassifolia* root wood hexane extract (BR 640/N192803) against NCI-60 panels, with the highest activity against the colon cancer HCT-15 cell line. **32**
Figure S18. Cytotoxicity of the *Salacia crassifolia* root wood hexane extract (BR 640/N192803) in the NCI-60 cell five-dose screen. **33**
Figure S19. Composite of the NCI-60 dose response curves of the *Salacia crassifolia* root wood hexane extract (BR 640/N192803) with higher activity against the colon cancer HCT-15 cell line. **34**

F. *Salacia elliptica*

Figure S20. Dose response curves of the *Salacia elliptica* root wood ethyl acetate extract (BR 652/N192805) against NCI-60 panels, with the highest activity against the leukemia MOLT-4 cell line. **35**

Figure S21. Mean bar graphs of the *Salacia elliptica* root wood ethyl acetate extract (BR 652/N192805) in the NCI-60 cell five-dose screen. **36**

Figure S22. Composite of the NCI-60 dose response curves of the *Salacia elliptica* root wood ethyl acetate extract (BR 652/N192805) with higher activity against the leukemia MOLT-4 cell line. **37**

G. *Casearia sylvestris var. lingua*

Figure S23. Dose response curves of the *Casearia sylvestris var. lingua* stem wood hexane extract (BR 177/N192825) against NCI-60 panels, with the highest activity against the non-small cell lung cancer NCI-H522. .... **38**

Figure S24. Mean bar graph of the *Casearia sylvestris var. lingua* stem wood hexane extract (BR 177/N192825) in the NCI-60 cell five-dose screen. **39**

Figure S25. Composite of the NCI-60 dose response curves of the *Casearia sylvestris var. lingua* stem wood hexane extract (BR 177/N192825) with higher activity against the non-small cell lung cancer NCI-H522. **40**

H. *Cupania vernalis*

Figure S26. Dose response curves of the *Cupania vernalis* leaf hexane extract (BR 193/N192827) against NCI-60 panels, with different susceptibility. **41**

Figure S27. Mean bar graph of the *Cupania vernalis* leaf hexane extract (BR 193/N192827) in the NCI-60 cell five-dose screen. **42**

Figure S28. Composite of the NCI-60 dose response curves of the *Cupania vernalis* leaf hexane extract (BR 193/N192827). **43**

I. *Simarouba versicolor*

Figure S29. Dose response curves of the *Simarouba versicolor* root bark ethanol extract (BR 254/N192829) against NCI-60 panels, with the highest activity against the non-small cell lung cancer NCI-H522. .... **44**

Figure S30. Mean bar graph of the *Simarouba versicolor* root bark ethanol extract (BR 254/N192829) in the NCI-60 cell five-dose screen. **45**

Figure S31. NCI-60 dose response curves of the *Simarouba versicolor* root bark ethanol extract (BR 254/N192829) with higher activity against the non-small cell lung cancer NCI-H522. .... **46**

J. *Kielmeyera coriacea*

Figure S32. Dose response curves of the *Kielmeyera coriacea* stem wood hexane extract (BR 331/N192831) against NCI-60 panels, with different susceptibility. **47**

Figure S33. Mean bar graph of the *Kielmeyera coriacea* stem wood hexane extract (BR 331/N192831) in the NCI-60 cell five-dose screen. **48**

Figure S34. NCI-60 dose response curves of the *Kielmeyera coriacea* stem wood hexane extract (BR 331/N192831). **49**

K. ***Byrsonima crassa***

Figure S35. Dose response curves of the *Byrsonima crassa* root bark hexane extract (BR 411/N192833) against NCI-60 panels, with different susceptibility. **50**

Figure S36. Mean bar graph of the *Byrsonima crassa* root bark hexane extract (BR 411/N192833) in the NCI-60 cell five-dose screen. **51**

Figure S37. Composite of the NCI-60 dose response curves of the *Byrsonima crassa* root bark hexane extract (BR 411/N192833). **52**

L. ***Schinus terebinthifolia***

Figure S38. Dose response curves of the *Schinus terebinthifolia* leaf dichloromethane extract (BR 436/N192835) against NCI-60 panels, with the highest activity against the non-small cell lung cancer NCI-H522. .... **53**

Figure S39. Mean bar graph of the *Schinus terebinthifolia* leaf dichloromethane extract (BR 436/N192835) in the NCI-60 cell five-dose screen. **54**

Figure S40. Composite of the NCI-60 dose response curves of the *Schinus terebinthifolia* leaf dichloromethane extract (BR 436/N192835) with higher activity against the non-small cell lung cancer NCI-H522. .... **55**

M. ***Enterolobium gummiferum***

Figure S41. Dose response curves of the *Enterolobium gummiferum* stem bark hexane extract (BR 469/N192837) against 9 cell panels with different susceptibility. **56**

Figure S42. Mean bar graph of the *Enterolobium gummiferum* stem bark hexane extract (BR 469/N192837) in the NCI-60 cell five-dose screen. **57**

Figure S43. Composite of the NCI-60 dose response curves of the *Enterolobium gummiferum* stem bark hexane extract (BR 469/N192837). **58**

N. ***Plathymenia reticulata***

Figure S44. Dose response curves of the *Plathymenia reticulata* root wood hexan extract (BR 489/N192839) against 9 cell panels with different susceptibility. **59**

Figure S45. Mean bar graph of the *Plathymenia reticulata* root wood hexane extract (BR 489/N192839) in the NCI-60 cell five-dose screen. **60**

Figure S46. Composite of the NCI-60 dose response curves of the *Plathymenia reticulata* root wood hexane extract (BR 489/N192839). **61**

O. ***Psidium laruotteanum***

Figure S47. Dose response curves of the *Psidium laruotteanum* stem bark hexane extract (BR 549/N192841) against 9 cell panels with different susceptibility. **62**

Figure S48. Mean bar graph of the *Psidium laruotteanum* stem bark hexane extract (BR 549/N192841) in the NCI-60 cell five-dose screen. **63**

Figure S49. Composite of the NCI-60 dose response curves of the *Psidium laruotteanum* stem bark hexane extract (BR 549/N192841). **64**

P. ***Lippia rotundifolia***

Figure S50. Dose response curves of the *Lippia rotundifolia* stem wood ethyl acetate extract (BR 660/N192843) against 9 cell panels with different susceptibility............ **65**

Figure S51. Mean bar graph of the *Lippia rotundifolia* stem wood ethyl acetate extract (BR 660/N192843) in the NCI-60 cell five-dose screen. **66**

Figure S52. Composite of the NCI-60 dose response curves of the *Lippia rotundifolia* stem wood ethyl acetate extract (BR 660/N192843). **67**

Q. ***Connarus suberosus***

Figure S53. Dose response curves of the *Connarus suberosus* root wood ethyl acetate extract (BR 693/N192845) against 9 cell panels with different susceptibility. **68**

Figure S54. Mean bar graph of the *Connarus suberosus* root wood ethyl acetate extract (BR 693/N192845) in the NCI-60 cell five-dose screen. **69**

Figure S55. Composite of the NCI-60 dose response curves of the *Connarus suberosus* root wood ethyl acetate extract (BR 693/N192845). **70**

Table S1. Brazilian Cerrado plant extracts submitted to high-throughput screening against a set of 8 cancer cell lines: colon (Colo205 and Km12), renal (A498 and U031), liver (HEP3B and SKHEP) and osteosarcoma (MG63 and MG63.3).

Family/Plant Species	Voucher number	Part of plant (solvent)	Vernacular Name	Traditional Use(s)	Vegetation types/ Collection season/ Soil types
**Alismataceae**					
Echinodorus macrophyllus (Kunth) Micheli	(UB) 3748	L[a] (d[1], hs[2])	Chapéu de couro	Anti-inflammatory, diuretic and antiarrhythmic [1]	Cerrado Ralo/ *Dry season/ Oxisol
**Anacardiaceae**					
Astronium fraxinifolium Schott	(UB) 3814	L (h[3], a[4], e[5]), SW[b] (h, e, w[6]), SB[c] (h, a, e), RW[d] (a, e, w), RB[e] (h, a, e, w)	Gonçalo-alves, Pau-Gonçalves	Antimicrobial, used to treat diarrhea and hemorrhoids, skin ulcers and rheumatism [2]	Cerrado sensu stricto/ Dry season/ Oxisol
Schinus terebinthifolia Raddi	(UB) 3753	L (h, d, hs), SW (h, hs, al[7]), SB (h, d, hs), RW (hs, al), RB (h)	Pimenta-rosa, Aroeira-vermelha	Treatment of tumors and leprosy [3]	Mata Ciliar/ Dry season/ Oxisol
**Annonaceae**					
Annona crassiflora Mart.	(UB) 3700	L (h, e), SW (h, e), SB (h, e), RW (e), RB (e)	Araticum	Treatment of fever and Chagas disease [4]	Cerrado sensu stricto/ Dry season/ Oxisol
Cardiopetalum calophyllum Schltdl.	(UB) 3703	L (h, e), SW (h, e), SB (h, e), R[f] (h, e)	Imbira	Treatment of fever [4]	Mata Ciliar/ Dry season/ Inceptisol

Duguetia furfuracea (A. St. Hil.) Saff.	(UB) 3679	L (h, e), SW (e), RW (h, e), RB (h, e), S[g] (h, e)	Pinha-de-guará, Araticum-seco	Treatment of rheumatism and stomach pains [5]	Cerrado sensu stricto/ Dry season/ Oxisol
Xylopia aromatica (Lam.) Mart	(UB) 3699	L (h, e), SW (h, e), SB (h, e), RW (h, e), RB (h, e)	Pimenta de macaco	Carminative properties [4]	Cerrado sensu stricto/ Dry season/ Oxisol
Xylopia emarginata Mart.	(UB) 3690	L (h, e), SW (h), SB (h, e), RW (h, e), RB (h, e)	Pindaíba-do-brejo	Bactericidal properties [4]	Mata Ciliar/ Dry season/ Oxisol
**Apocynaceae**					
Aspidosperma macrocarpon Mart.	(UB) 3692	L (h, e), SW (h, e), SB (h, e), RW (h, e), RB (h, e)	Pau-pereira, Peroba-gigante-do-cerrado	Used in the treatment of malaria and as an anti-inflammatory [6]	Cerrado sensu stricto/ *Rainy season/ Oxisol
Aspidosperma tomentosum Mart.	(UB) 3744	L (h, d, hs, al), SW (h, d, hs), SB (h, d, hs), R (d, hs)	Peroba-do-Cerrado	Treatment of malaria, leishmaniasis, cancer, inflammations, fever and rheumatism [7]	Cerrado sensu stricto/ Dry season/ Oxisol
Condylocarpon isthmicum (Vell.) A. DC.	(UB) 3663	L (h, e), SW (h, e), SB (h, e)	Cipó de leite	Used as an aromatic plant [8]	Mata Ciliar/ Rainy season/ Inceptisol
Hancornia pubescens Nees & C. Mart.	(UB) 3677	L (h, e), RW (h, e), RB (h, e)	Mangaba	Treatment of digestive problems [6]	Campo sujo de Cerrado/ Rainy season/ Oxisol
Himatanthus obovatus (Müll. Arg.) Woodson	(UB) 3678	L (h, e), RW (h, e), RB (h, e)	Tiborna, Pau-de-leite	Used to treat cancer, herpes and helminthiases [4]	Campo fechado de Cerrado/ Rainy season/ Oxisol

Peschiera affinis (Müll. Arg.) Miers	(UB) 3717	SW (h, e), SB (h, e), R (h, e)	Leiteiro, Mata-pasto, Grão de galo	Used as anti-microbial and anti-tumor [9]	Cerrado sensu stricto/ Rainy season/ Inceptisol
**Araliaceae**					
Didymopanax macrocarpus (Cham. & Schltdl.) Seem.	(UB) 3821	L (d), SB (h, a, e)	Mandiocão, Verga D'anta	Analgesic [10]	Cerrado sensu stricto/ Dry season/ Oxisol
**Asteraceae**					
Eremanthus glomerulatus Less.	(UB) 3721	L (h, e), SW (h, e), SB (h, e), RW (h, e), RB (h, e)	Candeia	Astringent [11]	Cerrado sensu stricto/ Dry season/ Oxisol
Eremanthus sphaerocephalus (DC.) Baker	(UB) 3708	C^h (e)	João-bobo	Treatment of gastric diseases [12]	Cerrado sensu stricto/ Dry season/ Oxisol
Piptocarpha macropoda (DC.) Baker	(UB) 3680	L (h, e), SB (h, e)	Cambará preto	No use reported	Mata Ciliar/ Rainy season/ Inceptisol
Piptocarpha rotundifolia (Less.) Baker	(UB) 3676	L (h, e), SB (h, e), RW (h), RB (h, e)	Paratudo, infalível	Treatment of wounds and used as an anti-syphilitic [2]	Cerrado sensu stricto/ Dry season/ Oxisol
**Bignoniaceae**					
Anemopaegma arvense (Vell.) Stellfeld ex J.F. Souza	(UB) 3691	L (h, e), S (h, e), R (h, e), F^i (h, e)	Catuaba	Aphrodisiac and central nervous system stimulant [13]	Cerrado sensu stricto/ Dry season/ Inceptisol

Anemopaegma chamberlaynii (Sims) Bureau & K. Schum.	(UB) 3715	L (h), S (h, d, e)	Cipó preto	No use reported	Mata Ciliar/ Dry season/ Inceptisol
Arrabidaea florida DC.	(UB) 3714	L (e), FS[j] (h, d, hs)	Cipó-neve	No use reported	Mata Ciliar/ Dry season/ Oxisol
Cybistax antisyphilitica (Mart.) Mart.	(UB) 3696	L (h, e), SW (h, e), SB (h, e), S (h, e), F (h, e)	Caroba-brava, ipê-branco	Depurative, antisyphilitic and diuretic [14]	Cerrado sensu stricto/ Dry season/ Oxisol
Jacaranda ulei Bureau & K. Schum.	(UB) 3791	Rz[k] (ch[8], a, e), AP[l] (a)	Carobinha-do-campo	Treatment of prostate and ovary inflammations; allergies; scabies; syphilis; constipation; liver dysfunction; together with blood purification and cholesterol reduction [15]	Cerrado sensu stricto/ Dry season/ Oxisol
Tabebuia caraiba (Mart.) Bureau	(UB) 3701	L (h, e), SW (h, e, hs), SB (h, e), RW (e), RB (e)	Ipê-amarelo-do cerrado, pau-d'arco	Used to treat common colds, chills and inflammations; purgative and anti-syphilitic [2]	Cerrado sensu stricto/ Dry season/ Oxisol
Zeyheria montana Mart.	(UB) 3799	S (h, a, e), RW (h), RB (a)	Bolsa-de-pastor, Chapéu-de-frade	Used to treat skin conditions; anti-syphilitic [10]	Cerrado sensu stricto/ Dry season/ Oxisol

Burseraceae

Protium heptaphyllum (Aubl.) Marchand	(UB) 3689	L (h, e), SW (e), SB (e), RW (e), RB (h, e)	Breu-branco-verdadeiro, almecegueira-vermelha	Used as a wound healer and expectorant, antiulcerogenic and anti-inflammatory [16]	Mata Ciliar/ Dry season/ Oxisol
Protium ovatum Engl.	(UB) 3694	L (h, d, e), SB (h), S (h, e), R (h, e), F (h, e)	Breu-do-cerrado	Analgesic, expectorant and wound healer [17]	Cerrado sensu stricto/ Dry season/ Inceptisol
**Calophyllaceae**					
Calophyllum brasiliense Cambess.	(UB) 3754	L (h, d, hs), SW (h, d, a, hs), SB (h, d, hs), RW (h, d, hs), RB (h, d), R (hs, al)	Guanandi, guarandi	Used to treat rheumatism, varicose veins, hemorrhoids and chronic ulcers [16]	Mata Ciliar/ Dry season/ Inceptisol
Kielmeyera coriacea Mart. & Zucc.	(UB) 3745	L (h, d, hs), SW (h, d, a, hs), SB (h, d, hs), RW (h, d), RB (h, d), F (h, hs)	Pau-santo	Used to treat fungal and bacterial infections, malaria, schistosomiasis, leishmaniasis [18]	Cerrado sensu stricto/ Dry season/ Oxisol
**Celastraceae**					
Cheiloclinium cognatum (Miers) A.C. Sm.	(UB) 3805	L (h, a), SW (h, a, e), SB (h, w)	Saputá	Treatment of fever and edema [19]	Mata Ciliar de Interfluvio/ Rainy season/ Inceptisol
Plenckia populnea Reissek	(UB) 3747	L (a, e), SW (a), SB (a, e)	Marmeleiro-do-campo	Allergy treatments and wound healing [10]	Cerrado sensu stricto/ Dry season/ Oxisol
Salacia crassifolia (Mart. ex Schult.) G. Don	(UB) 3776	L (a, e, w), SW (h, a, e), SB (h, a, e), RW (h)	Bacupari-do-Cerrado, Bacupary de caapuêra,	Used as anti-microbial and anti-tumor [20]	Cerrado sensu stricto/ Dry season/ Oxisol
Salacia elliptica (Mart.) G. Don	(UB) 3819	L (a, e), SW (h, a), RW (a)	Laranjeira do Cerrado	Laxative [21]	Campo sujo de Cerrado/ Dry season/ Inceptisol

**Clusiaceae**					
Clusia pernambucensis G. Mariz	(UB) 3771	L (h), SW (h, a), SB (h, a, e)	Cebolinha da mata	No use reported	Mata Ciliar em galeria/ Dry season/ Inceptisol
**Combretaceae**					
Terminalia fagifolia Mart. & Zucc.	(UB) 3812	L (h, a, e, w), SW (a, e, w), SB (h, a, e, w), RB (h), M^m (a)	Capitão-do-Cerrado	Used to treat canker sores and tumors [22]	Campo aberto de Cerrado/ Dry season/ Oxisol
Terminalia argentea Mart. & Zucc.	(UB) 3808	L (h, d)	Cachaporra-do-gentio	Used to treat coughs, canker sores and common colds [2]	Cerrado sensu stricto/ Rainy season/ Oxisol
**Connaraceae**					
Connarus suberosus Planch.	(UB) 3820	L (a), SW (h, a), RW (h, a, e), RB (h, a)	Cabelo-de-negro	Used to treat diarrhea [2]	Cerrado sensu stricto/ Dry season/ Oxisol
**Dilleniaceae**					
Davilla elliptica A. St.-Hil	(UB) 3773	L (h, a), SW (e), SB (h)	Cipó cabloco, sambaibinha	Used as an anti-inflammatory, anti-ulcerogenic, laxative and aphrodisiac [23]	Cerrado sensu stricto/ Dry season/ Inceptisol
**Ebenaceae**					
Diospyros hispida A. DC.	(UB) 3760	SW (h, d, hs), SB (h, d, hs), RW (w), R (h, a, e)	Caqui do Cerrado, fruta de boi	Treatment of infectious diseases [24]	Campo aberto de Cerrado/ Dry season/ Inceptisol
**Euphorbiaceae**					

Croton goyazensis Müll. Arg.	(UB) 3793	R (ch, e), AP (ch, a, e)	Pé-de-perdiz	Treatment of infections and general inflammation(s) [25]	Cerrado sensu stricto/ Dry season/ Oxisol
Croton urucurana Baill.	(UB) 3813	L (h, hs), SW (h, hs), SB (hs)	Sangue de dragão	Treatment of rheumatism, wounds, ulcers, diarrhea, and cancer [13]	Mata Ciliar/ Rainy season/ Oxisol
Maprounea guianensis Aubl.	(UB) 3772	L (d), SW (e), SB (h, a, e), RB (h, a, e)	Pinga-orvalho, milho-torrado	Used as an anti-diarrhea compound and to treat leg rashes [26]	Mata Ciliar em galeria/ Dry season/ Inceptisol
**Fabaceae**					
Andira humilis Mart. ex. Benth.	(UB) 3764	L (h, e), SW (a, e), SB (e), RW (h), RB (e)	Angelim rasteira, mata barata	Treatment of common colds, helminthiases and vaginal discharge [27]	Cerrado sensu stricto/ Dry season/ Oxisol
Andira vermifuga Mart. ex. Benth.	(UB) 3763	L (h)	Angelim	Purgative, emetic and narcotic effects [8]	Cerrado sensu stricto/ Dry season/ Oxisol
Chamaecrista desvauxii (Collad.) Killip	(UB) 3800	AP (a, e)	Rabo de pitu, vassourinha	Laxative and purgative [28]	Cerrado sensu stricto/ Rainy season/ Oxisol
Enterolobium ellipticum Benth.	(UB) 3739	L (h, d, hs), SW (h, hs), SB (h), S (h, hs), RW (h, d, hs, al), RB (h)	Tamboril, Favela branca	Used to treat lung infections [8]	Cerrado sensu stricto/ Rainy season/ Oxisol
Enterolobium gummiferum (Mart.) J.F. Macbr.	(UB) 3807	L (a), SW (h, a), SB (h), RW (h, a, e), RB (a)	Timburi, Orelha de macaco	Used to treat the lungs and dermatitis, antihelminthic [29]	Cerrado sensu stricto/ Dry season/ Oxisol
Plathymenia reticulata Benth.	(UB) 3794	SW (a), SB (h, e), RW (h), RB (h)	Vinhático-do-cerrado	Used to treat varicose veins, some inflammatory diseases, infections and cases of hemorrhaging resulting from an insect/tick bite [30]	Cerrado sensu stricto/ Dry season/ Oxisol

Sclerolobium aureum (Tul.) Baill.	(UB) 3818	L (a), SW (h, a, e), SB (a), RW (h, a, e), RB (a)	Gonçalo do campo	Used to treat fungal infections and as a hepatoprotector [31]	Cerrado sensu stricto/ Dry season/ Oxisol
Stryphnodendron adstringens (Mart.) Coville	(UB) 3740	L (h, d, hs, al), SW (h, d), SB (h, d, hs), RW (h, d), RB (h)	Barbatimão	Used as an anti-inflammatory, astringent and for healing wounds and vaginal infections [16]	Cerrado sensu stricto/ Dry season/ Oxisol
Vatairea macrocarpa (Benth.) Ducke	(UB) 3815	L (a), SW (h, a, e), SB (a), RW (h, a), RB (h, a)	Amargoso, angelim-do-Cerrado, maleiteira	Treatment of diabetes, superficial mycoses and cancer [31]	Cerrado sensu stricto/ Dry season/ Inceptisol
**Hypericaceae**					
Vismia decipiens var. *pyrifolia* Schltdl. & Cham.	(UB) 3769	L (h, a, e), SW (h, a, e), SB (h, a, e, w), RW (h), RB (e), F (h, a)		No use reported	Mata Ciliar/ Dry season/ Oxisol
**Magnoliaceae**					
Talauma ovata A.St.-Hil.	(UB) 3738	L (h, e), SW (h, e), SB (h, e)	Baguaçu	Treatment of diabetes and fever [6]	Mata Ciliar/ Rainy season/ Oxisol
**Malpighiaceae**					
Byrsonima coccolobifolia Kunth	(UB) 3774	RW (h, a), SW (e), RB (h, e), SB (h, e)	Murici-pequeno	Treatment of gastrointestinal disorders [26]	Campo sujo de Cerrado/ Dry season/ Inceptisol
Byrsonima crassa Nied.	(UB) 3743	L (h, d, hs), SW (h, hs), SB (h, hs, al), RW (d, hs), RB (h)	Murici-cascudo, murici-vermelho	Antiemetic, diuretic, febrifuge, used to treat ulcers, gastritis and diarrhea [32]	Cerrado sensu stricto/ Dry season/ Oxisol

**Meliaceae**					
Guarea guidonia (L.) Sleumer	(UB) 3712	L (h, e), S (h, e), R (h, e)	Cedro-macho, açafroa	Astringent, purgative, febrifuge, emetic and abortive [16]	Mata Ciliar/ Dry season/ Oxisol
Guarea kunthiana A. Juss.	(UB) 3710	L (h, e), S (h, d, e), R (h, d, e), F (e)	Jatuaúba	Antimalarial and used to treat stomach pains [6]	Mata Ciliar/ Dry season/ Oxisol
**Myrtaceae**					
Blepharocalyx salicifolius (Kunth) O. Berg	(UB) 3798	L (h, a, e, w), SW (h, a), SB (h, a), RB (w)	Anacahuita	Used as an external astringent, treatment of diarrhea, leucorrhoea, urethritis, rectal prolapse; antirheumatic, hypoglycemic, diuretic [16]	Cerrado sensu stricto/ Dry season/ Oxisol
Eugenia dysenterica DC.	(UB) 3803	L (hs)	Cagaita	The fruit is a laxative and the leaves are used to treat diarrhea [33]	Cerrado sensu stricto/ Rainy season/ Oxisol
Myrcia linearifolia Cambess.	(UB) 3817	R (h, a), AP (a)	Ratanhia, araçazinho	An edible fruit, with leaves used for their allelopathic activity [34]	Cerrado sensu stricto/ Dry season/ Inceptisol
Psidium laruotteanum Cambess.	(UB) 3810	L (h, a), SW (h, a), SB (h)	Araça cascudo	Used against stomachache and colic, colds, diarrhea, sore throat and worms [26]	Cerrado sensu stricto/ Rainy season/ Oxisol
**Nyctaginaceae**					

Guapira noxia (Netto) Lundell	(UB) 1537	SW (a), RW (a), RB (a)	Pau-lepra, capa-rosa	No use reported	Cerrado sensu stricto/ Dry season/ Inceptisol
Neea theifera Oerst.	(UB) 3821	L (a), SW (h, a), RW (h, a, e), RB (a)	Capa-rosa-do-campo	Used to treat diarrhea, enterocolitis and enterorrhagia [8]	Cerrado sensu stricto/ Dry season/ Inceptisol
**Ochnaceae**					
Ouratea floribunda Engl.	(UB) 3713	L (d), F (h)	Caju bravo	Used as an astringent, tonic, vermifuge and to treat paralysis, erysipelas, wounds in the uterus, ulcers, gastric disorders and wound healing [35]	Cerrado sensu stricto/ Dry season/ Oxisol
**Opiliaceae**					
Agonandra brasiliensis Miers ex Benth. & Hook. f.	(UB) 3797	L (h, a, e), R (h)	Pau-marfim, quina de veado	Used to treat rheumatism, healing and treating bronchitis [29]	Cerrado sensu stricto/ Dry season/ Oxisol
**Primulaceae**					
Myrsine guianensis (Aubl.) Kuntze	(UB) 3795	L (h, a), SB (h, a, e, w), R (a)	Capororoca branca	No use reported	Cerrado sensu stricto/ Dry season/ Oxisol
Rapanea guianensis Aubl.	(UB) 3804	L (h, a, e), SW (h, e), RW (h, a, e), RB (w)	Capororoca	Used to treat snake bites, cleanse tumors and wounds [9]	Cerrado sensu stricto/ Rainy season/ Oxisol
**Rubiaceae**					
Chomelia pohliana Mull. Arg.	(UB) 3741	L (d, hs), S (d), R (hs)	Limaorana, mentolzinho	No use reported	Mata Ciliar/ Dry season/ Inceptisol

Palicourea rigida Kunth	(UB) 3722	L (h, d), R (h, d, hs), AP (h, e)	Congonha-dourada, douradinha	Treatment of urinary tract inflammation [36]	Cerrado sensu stricto/ Dry season/ Oxisol
Sabicea brasiliensis Wernham	(UB) 3709	R (h, e), F (h, e)	Sangue de cristo	Treatment of infections in female genitalia [37]	Cerrado sensu stricto/ Dry season/ Oxisol
Tocoyena formosa (Cham. & Schltdl.) K. Schum.	(UB) 3792	L (h, a, e)	Genipa-brava, Trombeta, Genipaparaná	Use for the treatment of rheumatic pains [38]	Cerrado sensu stricto/ Dry season/ Oxisol
**Rutaceae**					
Spiranthera odoratissima A. St.-Hil.	(UB) 3768	L (h, a, e), R (h, a, e), F (h)	Manacá	Used for the treatment of rheumatism, gout, acne, boils, kidney infections and inflammation [39]	Cerrado sensu stricto/ Dry season/ Oxisol
Zanthoxylum rhoifolium Lam.	(UB) 3770	L (ch, a, e), SW (h, a, e, w), SB (a), RW (h, a, e, w), RB (ch, h, a, e)	Mamica-de-cadela, mamica-de-porca	Treatment and prevention of malaria [40]	Cerrado sensu stricto/ Dry season/ Inceptisol
**Salicaceae**					
Casearia sylvestris var. *lingua* (Cambess.) Eichler.	(UB) 3693	L (h, e), SW (h), SB (e), RW (h, e), RB (e), F (h, e)	Erva de Lagarto	Used as an anti-inflammatory, analgesic, antibiotic and in anti-cancer treatments [4]	Cerrado sensu stricto/ Dry season/ Inceptisol
**Sapindaceae**					
Cupania vernalis Cambess.	(UB) 3695	L (h, e), SW (h, e), SB (h, e), RW (h, e), RB (h, e), R (e)	Camboatã vermelho, olho de cotia	Treatment of fever and inflammation or as a tonic [4]	Mata Ciliar de Galeria/ Rainy season/ Oxisol

Magonia pubescens A.St. -Hil.	(UB) 3702	L (h, e), SB (h, e), RW (h, e), RB (h, e), R (h, e), F (h), FS (e)	Tingui	Insecticide and used to kill lice [29]	Cerrado sensu stricto/ Dry season/ Oxisol
Matayba guianensis Aubl.	(UB) 3697	SW (h, e), SB (h, e), RW (h, d, hs), RB (h, e), R (al)	Camboatá, assa-leitão	Edible fruit	Cerrado sensu stricto/ Rainy season/ Inceptisol
Serjania lethalis A. St. -Hil.	(UB) 3716	L (h, e), SW (h, e), SB (e), RB (h, e)	Timbó do cerrado	Used as an analgesic, anti-inflamatory and piscicide; Ichtyotoxic properties [4]	Cerrado sensu stricto/ Rainy season/ Inceptisol
**Sapotaceae**					
Chrysophyllum soboliferum Rizzini	(UB) 3733	L (h, e)	Fruta de tatu	Edible fruit	Cerrado sensu stricto/ Dry season/ Oxisol
Pouteria gardneri (Mart. & Miq.) Baehni	(UB) 3672	L (h, e), SW (e), RW (h, e), RB (h, e), R (h, e)	Sapotinha	Edible fruit	Mata Ciliar/ Rainy season/ Inceptisol
Pouteria ramiflora (Mart.) Radlk.	(UB) 3671	L (h, e), SW (h, e), SB (h, e), RW (h, e), RB (h, e)	Abiu do cerrado, pitomba de leite	Used on bruises [41]	Cerrado sensu stricto/ Dry season/ Oxisol
Pouteria torta (Mart.) Radlk.	(UB) 3674	L (h, d, e), SW (e), RW (h, e), RB (h)	Curiola; Grao de galo	Edible fruit and the stem used in the treatment of dysentery	Cerrado sensu stricto/ Rainy season/ Inceptisol
**Simaroubaceae**					
Simaba suffruticosa Engl.	(UB) 3790	L (a)	Calunga	Abortive [42]	Cerrado sensu stricto/ Rainy season/ Oxisol

Simarouba versicolor A. St. -Hil.	(UB) 3724	L (h, d, e), SB (h, e), S (e), RB (h, e), F (h, e)	Mata-cachorro	Used as a tonic to treat weakness; anti-helminthic, anti-hemorrhagic, anti-diarrhea, anti-dysenteric, anti-dyspepsia, anti-syphilitic, febrifuge and snake venom neutralizer [43]	Cerrado sensu stricto/ Dry season/ Oxisol
**Siparunaceae**					
Siparuna cujabana (Mart. ex Tul.) A. DC.	(UB) 3737	L (e), S (h, e), R (h, e), F (h, e)	Pau-limão	Anti-spasmodic [8]	Mata Ciliar/ Rainy season/ Oxisol
Siparuna guianensis Aubl.	(UB) 3106	L (h, e), SW (h, e), SB (h, e)	Negramina, capitiú	Used to relieve fever and body pain [44]	Mata Ciliar/ Rainy season/ Oxisol
**Solanaceae**					
Solanum lycocarpum A. St.-Hil.	(UB) 3720	L (h)	Lobeira	Used as tonic, emollient and antirheumatic; used to treat asthma, flu and colds [10]	Cerrado sensu stricto/ Rainy season/ Oxisol
**Styracaceae**					
Styrax ferrugineus Nees & Mart.	(UB) 2621	SW (h)	Laranjeira do campo	Used to treat fevers, burns and coughs; depurative [10]	Cerrado sensu stricto/ Dry season/ Oxisol
**Symplocaceae**					
Symplocos rhamnifolia A. DC.	(UB) 3331	SW (a), SB (a), RW (a), RB (a)	Congonha	Used when experiencing kidney problems; taken as a coffee or tea-like infusion [45]	Cerrado sensu stricto/ Dry season/ Inceptisol
**Verbenaceae**					

Lippia rotundifolia Cham.	(UB) 3796	L (h, a, e), SW (h, a), SB (a), R (h), FL[n] (h, a)	Rosmaninho, cha de pedestre	Used for relaxing baths and feet scaling [46]	Cerrado sensu stricto/ Dry season/ Inceptisol
**Vochysiaceae**					
Qualea grandiflora Mart.	(UB) 3746	L (h, d, hs), SW (h, d, e, hs), SB (h, d, hs), S (d), RB (d, hs), R (e, hs), FS (h, d, hs)	Pau-terra-do-cerrado	Antiseptic; indicated to treat diarrhea with blood, intestinal colic and amoebae [10, 29]	Cerrado sensu stricto/ Dry season/ Oxisol
Qualea parviflora Mart.	(UB) 3742	SW (h, d, hs), SB (d, hs), RW (h, d, hs, al), RB (hs), F (h)	Pau-terra-mirim	Used as an antiseptic and antiulcerogenic, and in the treatment of gastrointestinal disorders [47]	Cerrado sensu stricto/ Dry season/ Oxisol
Salvertia convallariodora A.St.-Hil.	(UB) 3777	L (h, a, e), SW (h, a, e)	Colher de vaqueiro	Gastro-protectant [48]	Cerrado sensu stricto/ Dry season/ Oxisol
**Zingiberaceae**					
Renealmia alpinia (Rottb.) Maas	(UB) 3719	L (h, e), Rz (h, d, hs), P[o] (h), LP[p] (h)	Paco-seroca	Used as febrifuge and to treat snake bites [49]	Mata Ciliar/ Rainy season/ Oxisol

All individuals were adults.
***The Cerrado climate is highly seasonal marked with dry (May-September) and rainy (October-April) seasons.**

Solvent: d[1]: dichloromethane, hs[2]: 90% hydroethanol, h[3]: hexane, a[4]: ethyl acetate, e[5]: ethanol, w[6]: water, al[7]: alkaloid extract, ch[8]: cyclohexane

Part of plant: [a]L: Leaf , [b]SW: Stem Wood, [c]SB: Stem Bark, [d]RW: Root Wood, [e]RB: Root Bark, [f]R: Root (Wood+ Bark), [g]S: Stem (Bark + Wood), [h]C: Capitulum, [i]F: Fruit, [j]FS: Fruit+Seed, [k]Rz: Rhizome, [l]AP: Aerial Parts, [m]M: Mucilage, [n]FL: Flower, [o]P: Pseudostem, [p]LP: Leaf + pseudostem

Table S2. Z-factors for plates used in initial screening and secondary dose response testing [50]**.**

Cell line	Primary screening	Secondary D/R screening*
COLO205 (colon)	0.35, 0.50	0.41, 0.58, 0.58, 0.50
KM12 (colon)	0.54, 0.40	0.87, 0.68, 0.46
UO31 (renal)	0.51, 0.42	0.42, 0.43, 0.44, 0.74
A498 (renal)	0.39, 0.41	0.39, 0.57, 0.42, 0.41
MG63 (osteosarcoma)	0.73, 0.78	0.62, 0.59, 0.72
MG63.3 (osteosarcoma)	0.58, 0.66	0.51, 0.58, 0.80
HEP3B (hepatocellular)	0.56, 0.67	Not tested
SKHEP (hepatocellular)	0.64, 0.72	Not tested

***The number of plates varied according to the number of active samples with that cell line.**

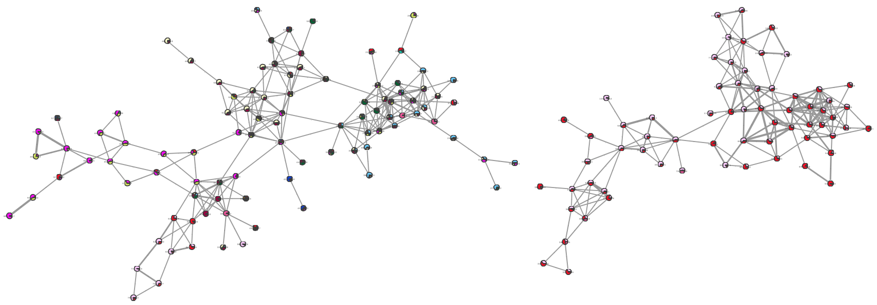

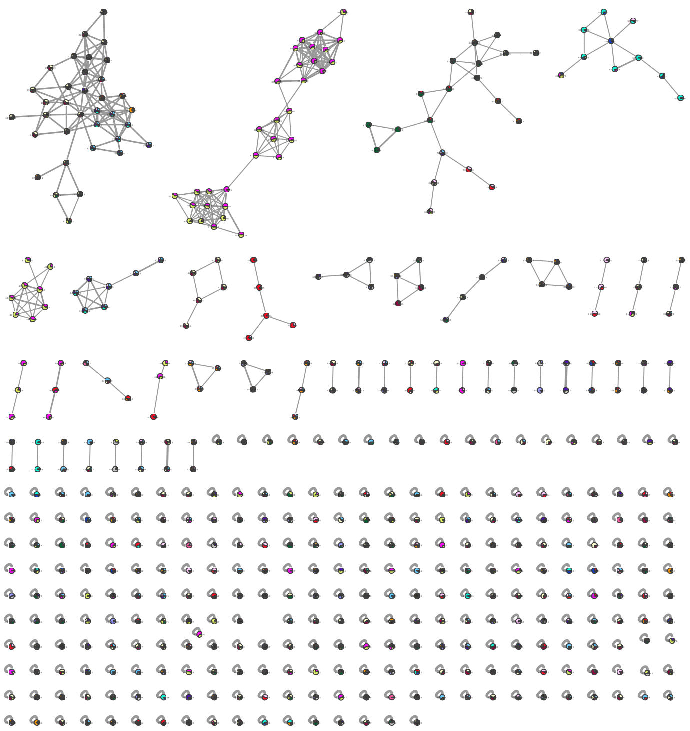

Figure S1. Complete molecular networking (MN) of 17 more active Cerrado plants.

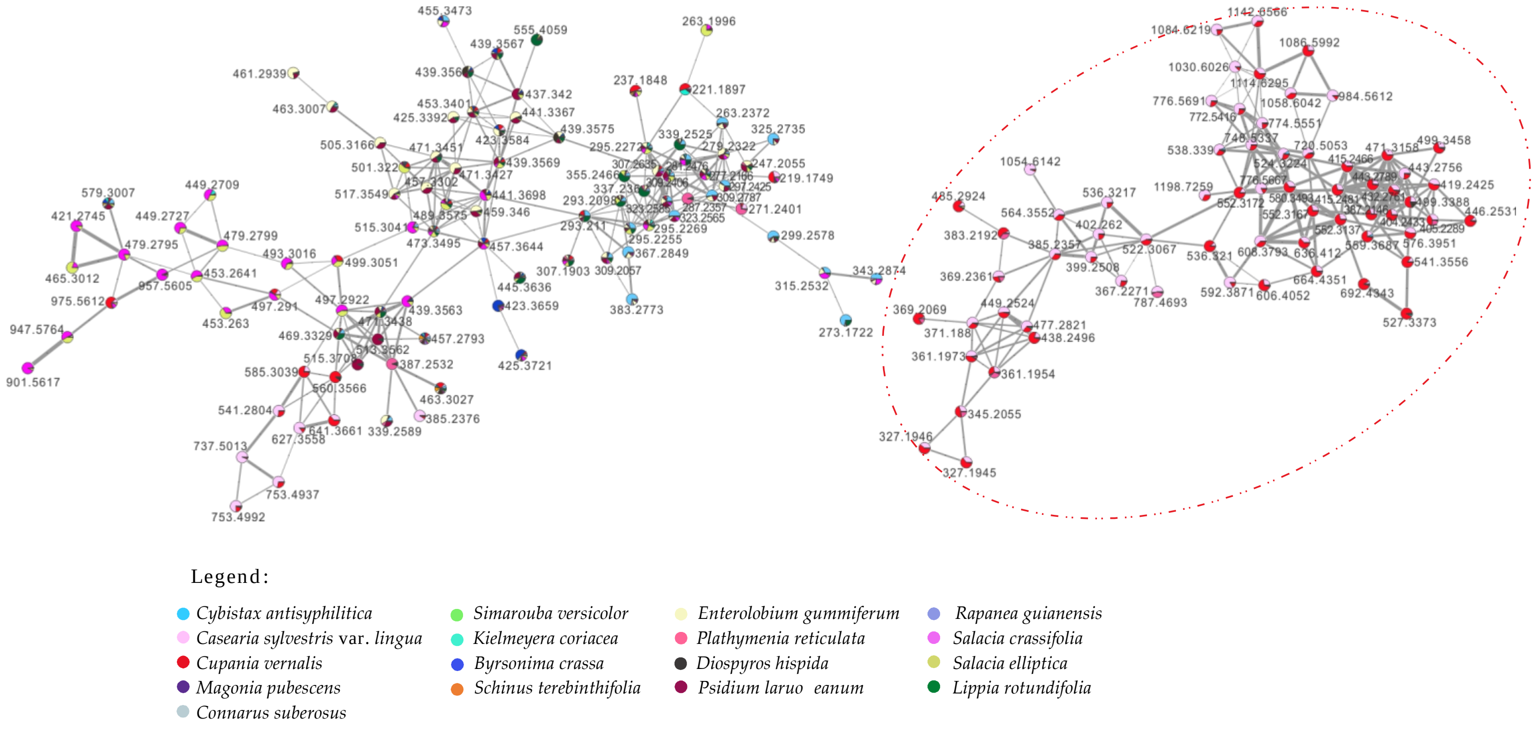

Figure S2. Expansion of the network, top portion.

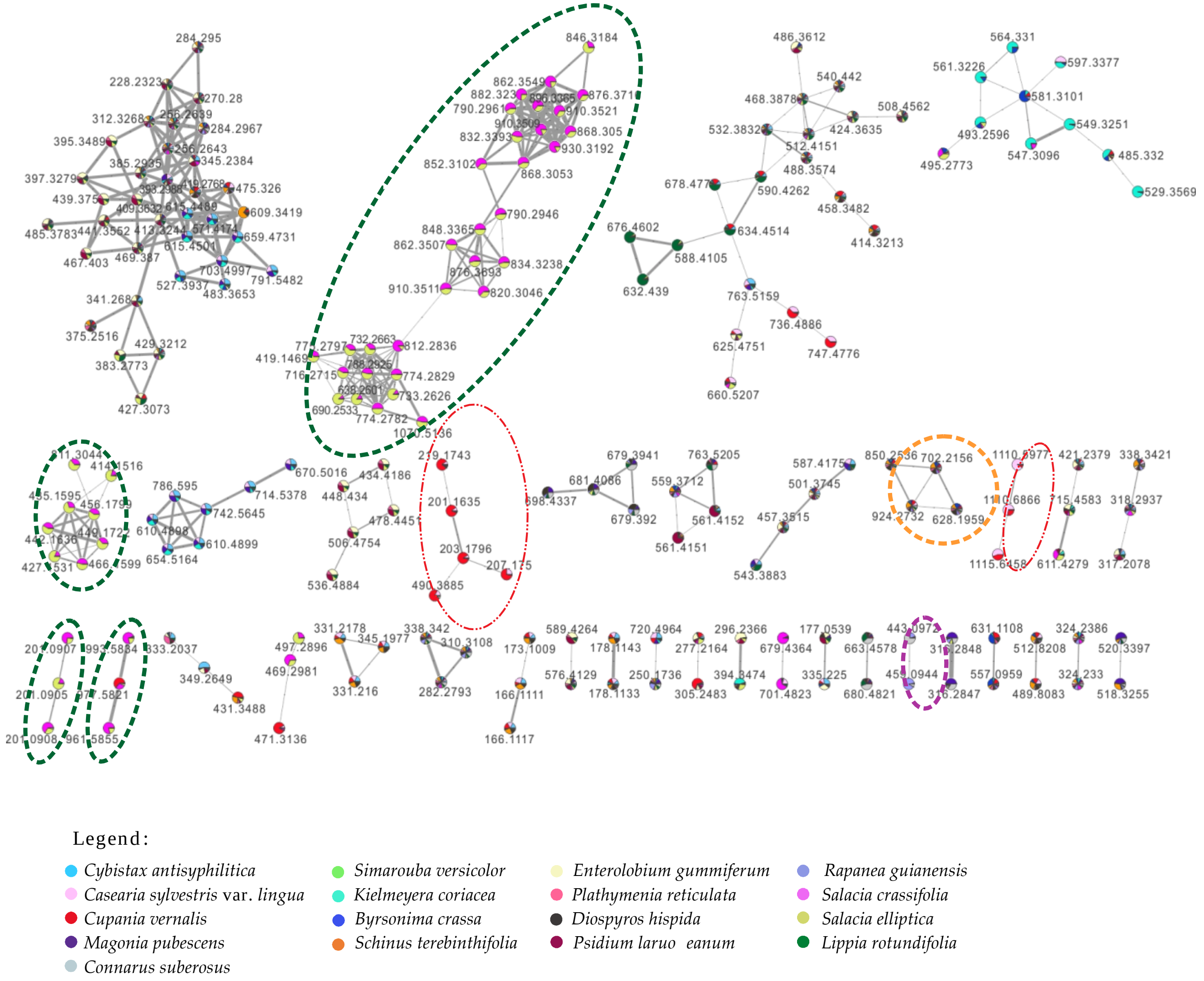

Figure S3. Expansion of the network, central portion.

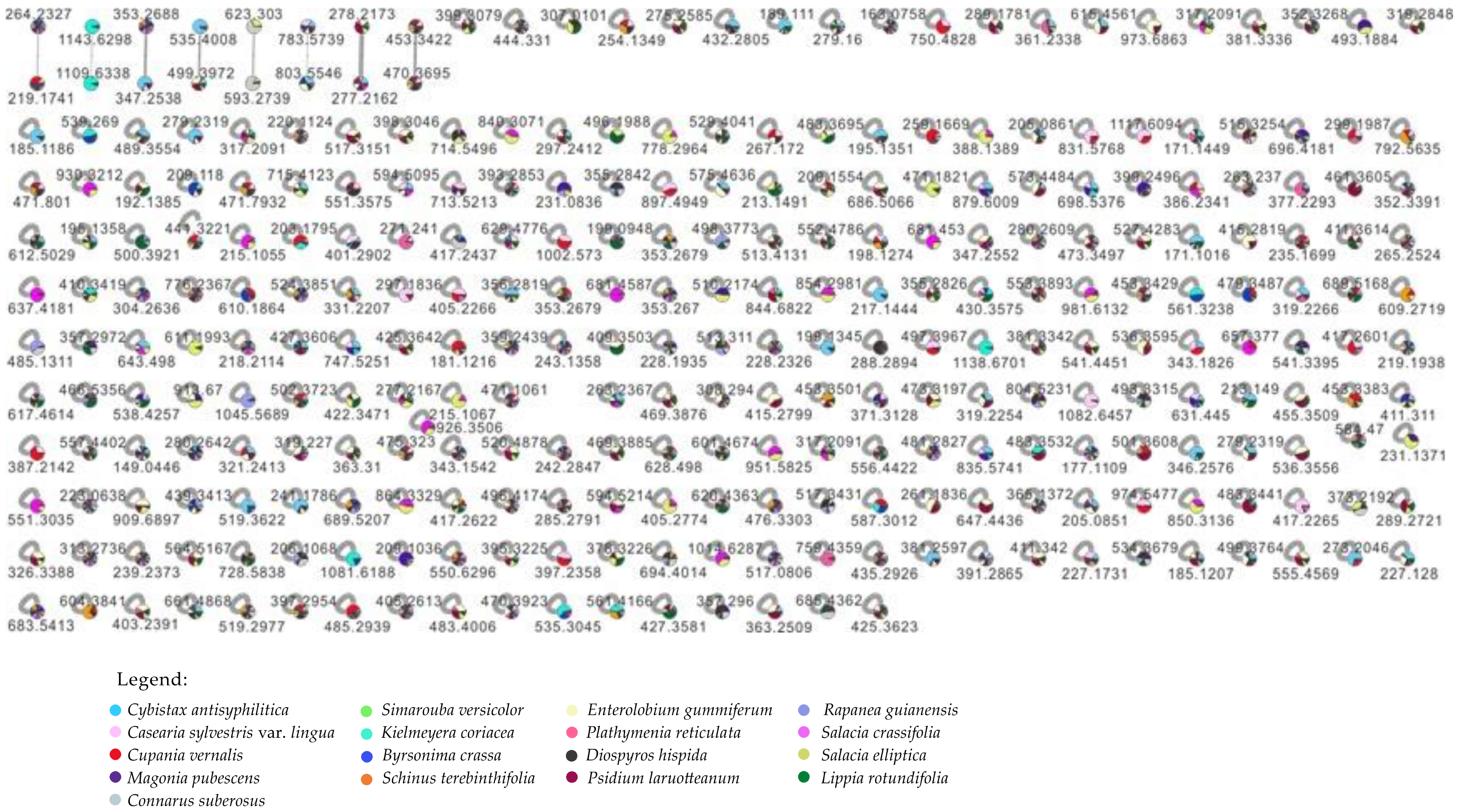

Figure S4. Expansion of the network, bottom portion.

Results graphs of NCI-60 screening S5 to S55

A. *Cybistax antisyphilitica*

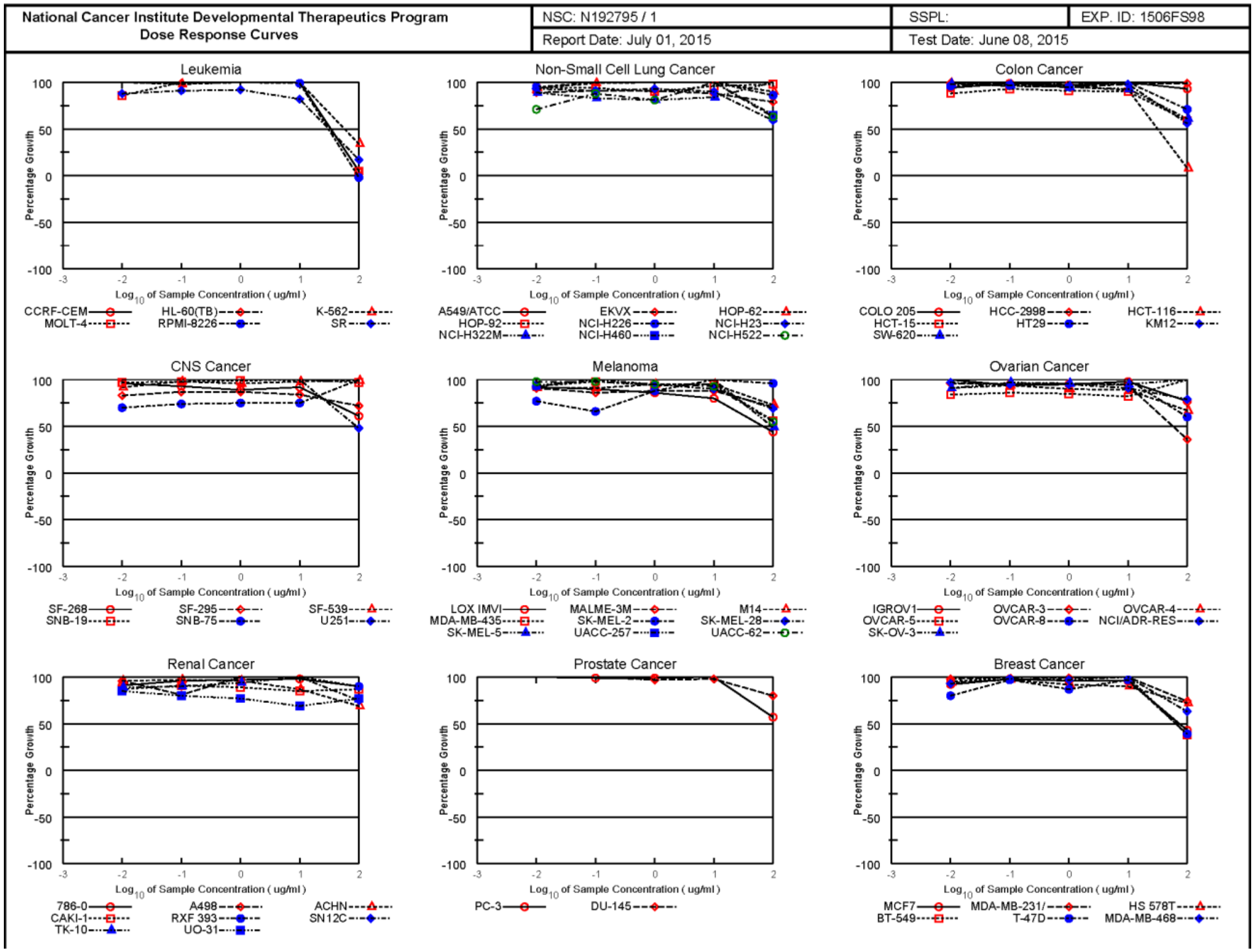

Figure S5. Dose response curves of the *Cybistax antisyphilitica* stem bark hexane extract (BR 125/N192795) against 9 cell panels with different susceptibility.

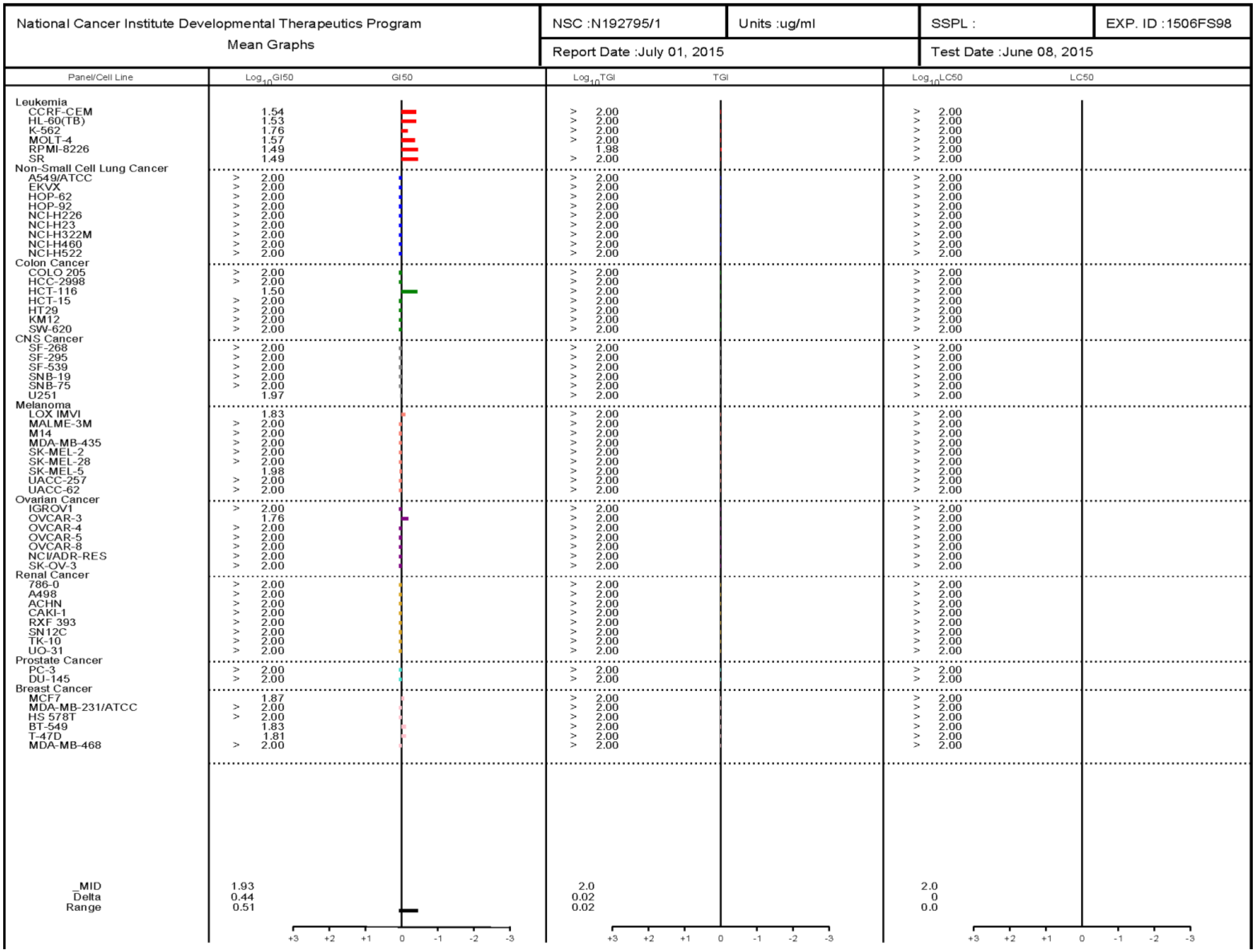

Figure S6. Mean bar graph of the *Cybistax antisyphilitica* stem bark hexane extract (BR 125/N192795) in the NCI-60 cell five-dose screen.

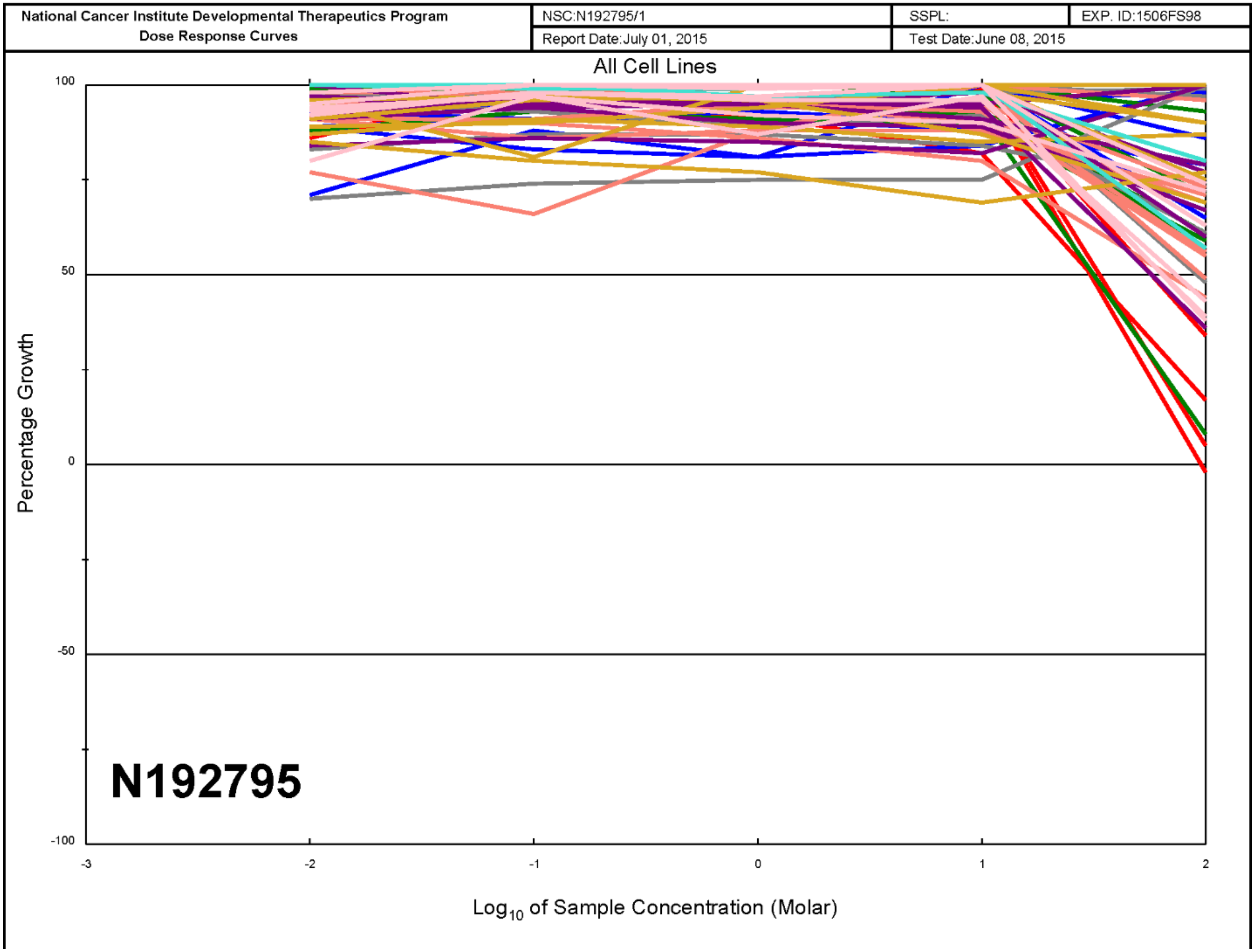

Figure S7. Composite of the NCI-60 dose response curves for the *Cybistax antisyphilitica* stem bark hexane extract (BR 125/N192795).

B. *Magonia pubescens*

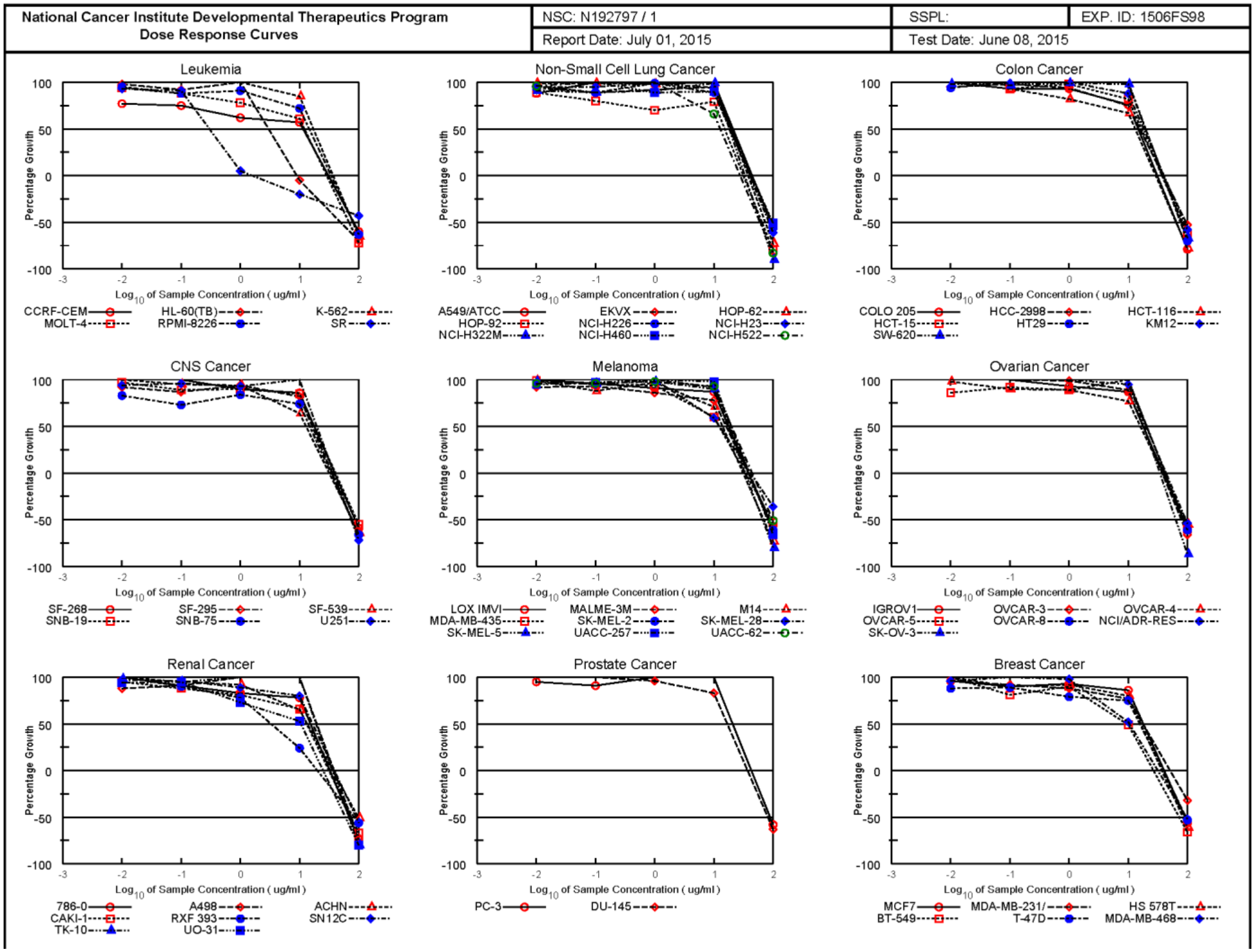

Figure S8. Dose response curves of the *Magonia pubescens* root wood ethanol extract (BR 204/N192797) against 9 cell panels with the highest activity against the leukemia SR cell line.

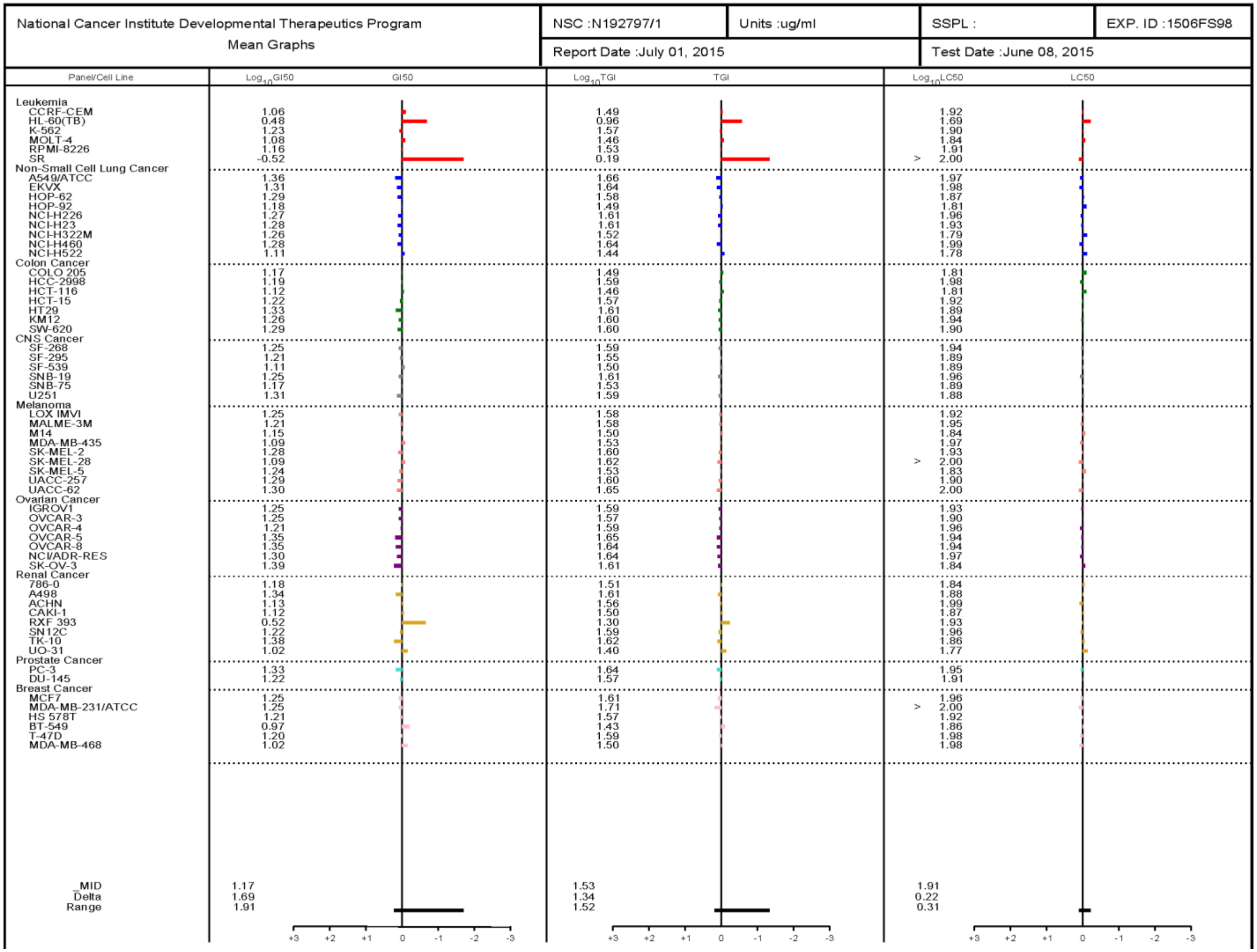

Figure S9. Mean bar graph of the *Magonia pubescens* root wood ethanol extract (BR 204/N192797) in the NCI-60 cell five-dose screen.

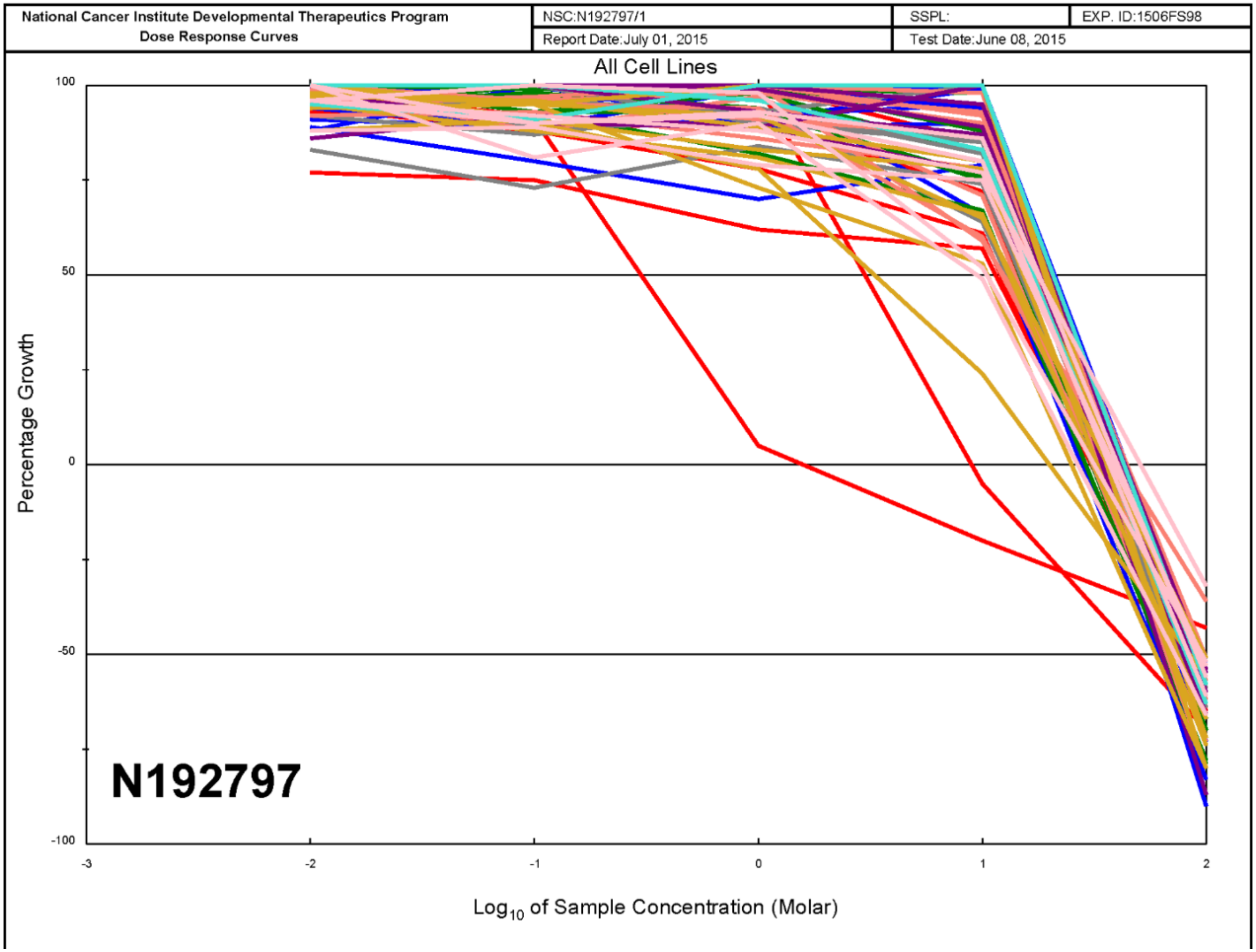

Figure S10. Composite of the NCI-60 dose response curve of the *Magonia pubescens* root wood ethanol extract (BR 204/N192797) with higher activity against the leukemia SR cell line.

C. *Diospyros hispida*

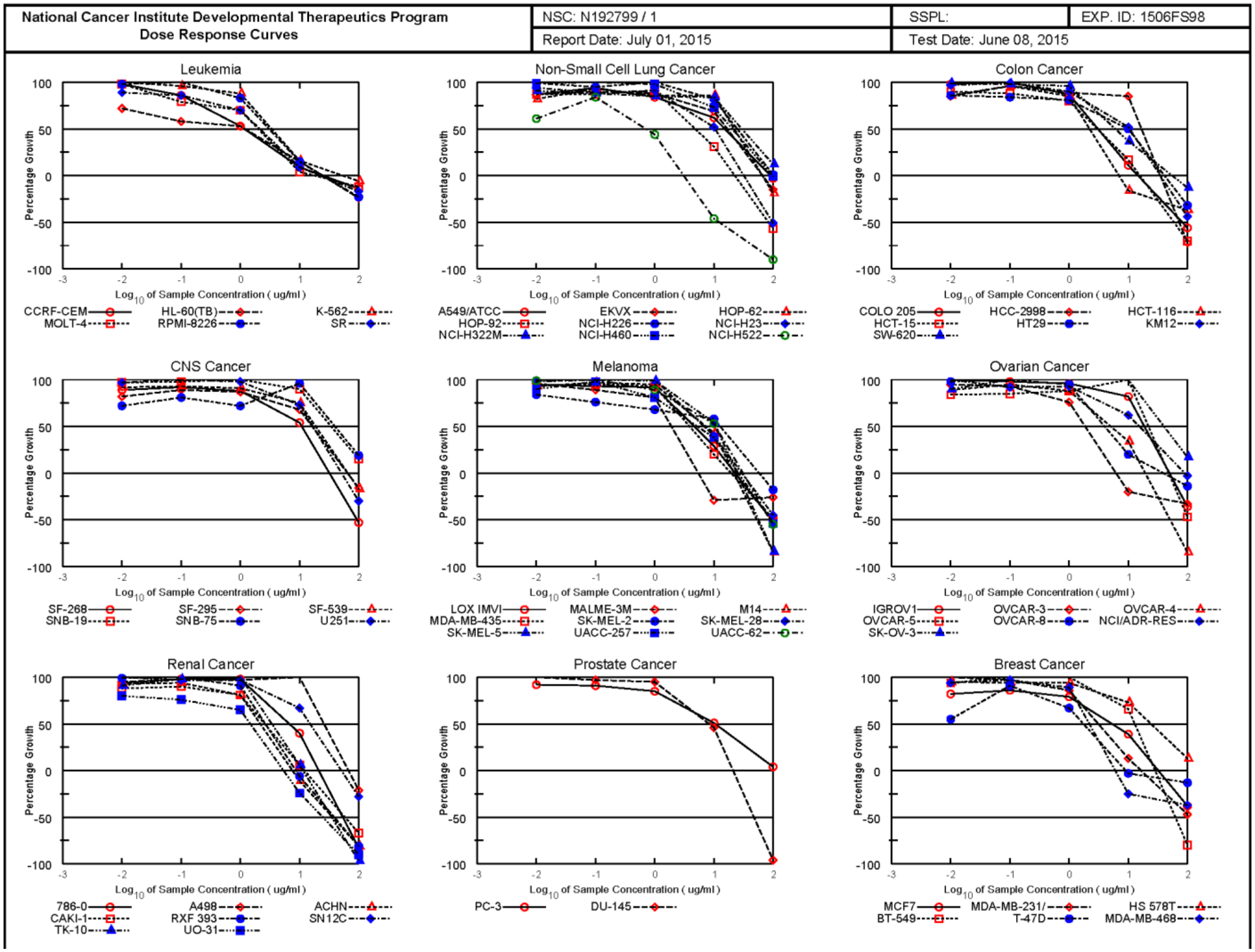

Figure S11. Dose response curves of the *Diospyros hispida* root (wood + bark) ethyl acetate extract (BR 501/N192799) against NCI-60 panels, with the highest activity against the non-small cell lung cancer NCI-H522.

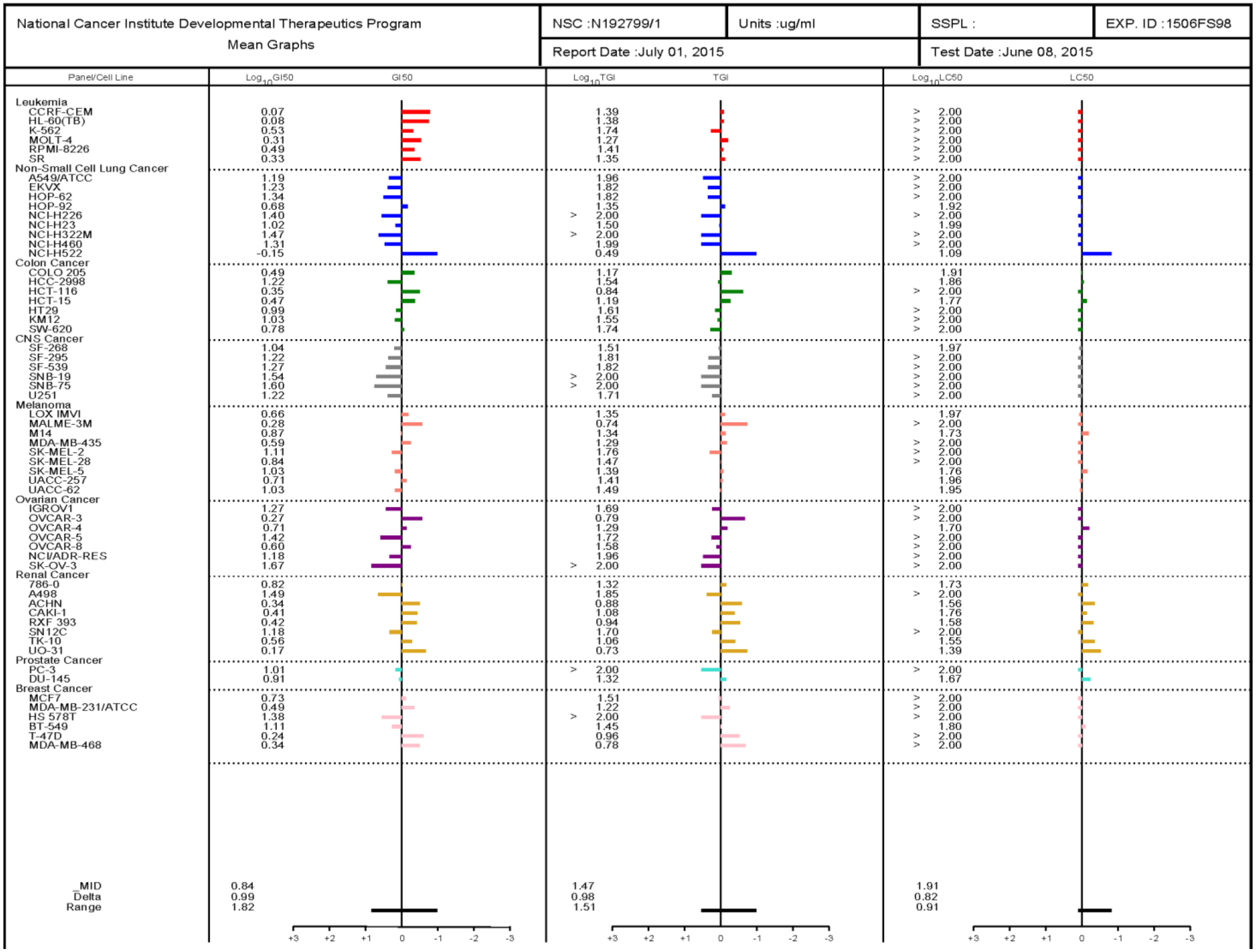

Figure S12. Mean bar graph of the *Diospyros hispida* root (wood + bark) ethyl acetate extract (BR 501/N192799) in the NCI-60 cell five-dose screen. Sensitive cell lines project to the right of the mean and resistant cell lines project to the left.

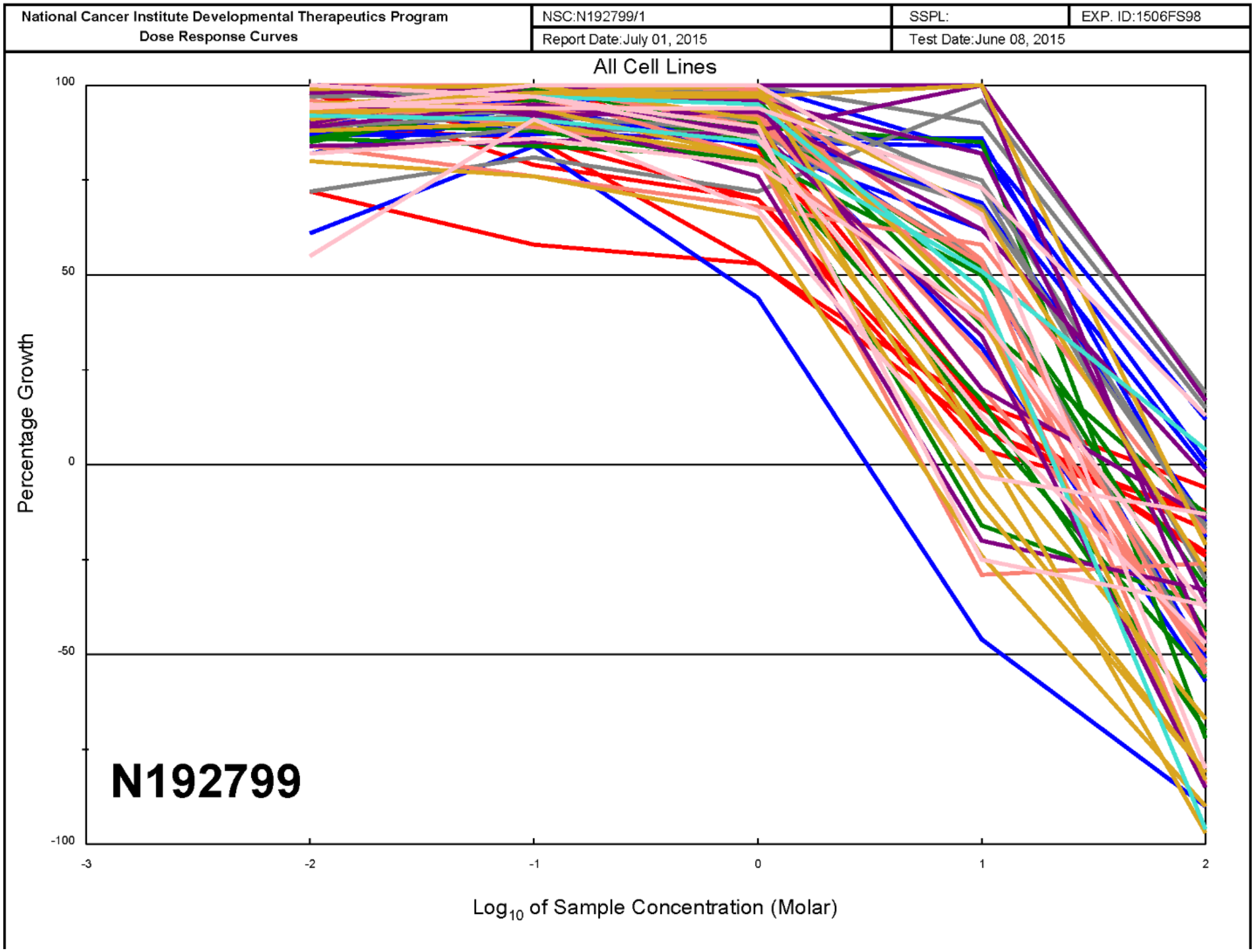

Figure S13. Composite of the NCI-60 dose response curves of the *Diospyros hispida* root (wood + bark) ethyl acetate extract (BR 501/N192799) with higher activity against the non-small cell lung cancer NCI-H522.

D. *Rapanea guianensis*

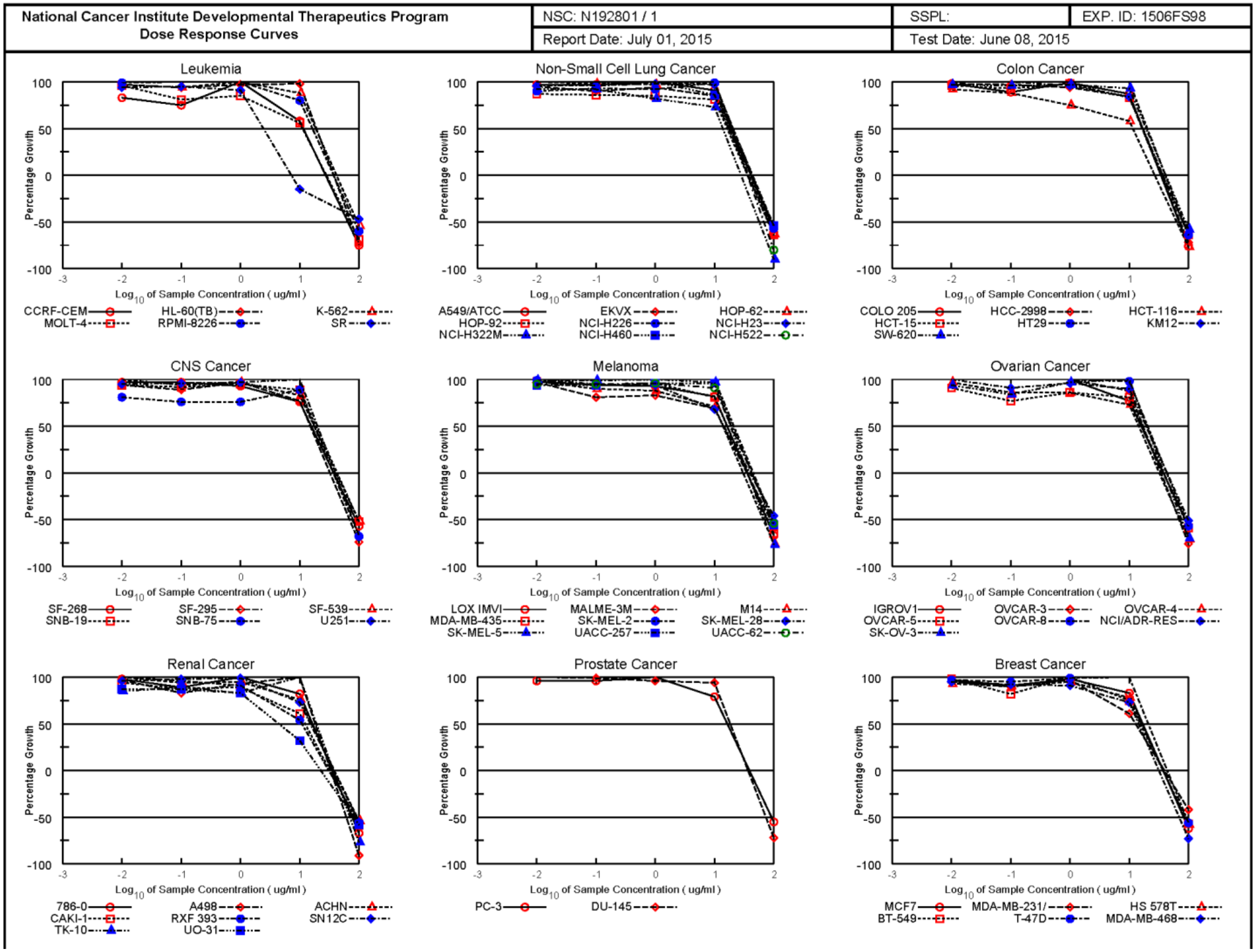

Figure S14. Dose response curves of the *Rapanea guianensis* root wood ethanol extract (BR 627/N192801) against NCI-60 panels, with the highest activity against the leukemia SR cell line.

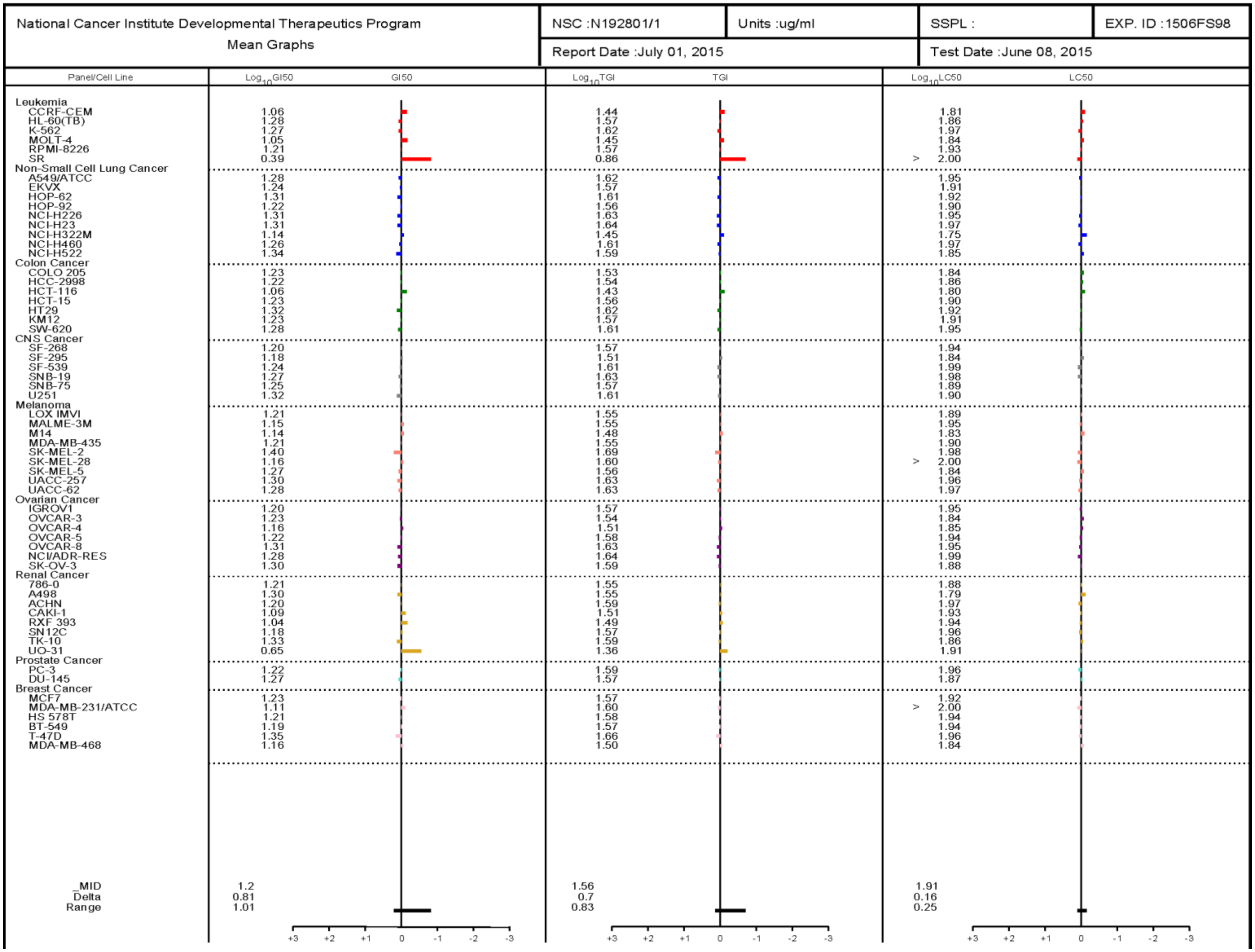

Figure S15. Mean bar graph of the *Rapanea guianensis* root wood ethanol extract (BR 627/N192801) in the NCI-60 cell five-dose screen.

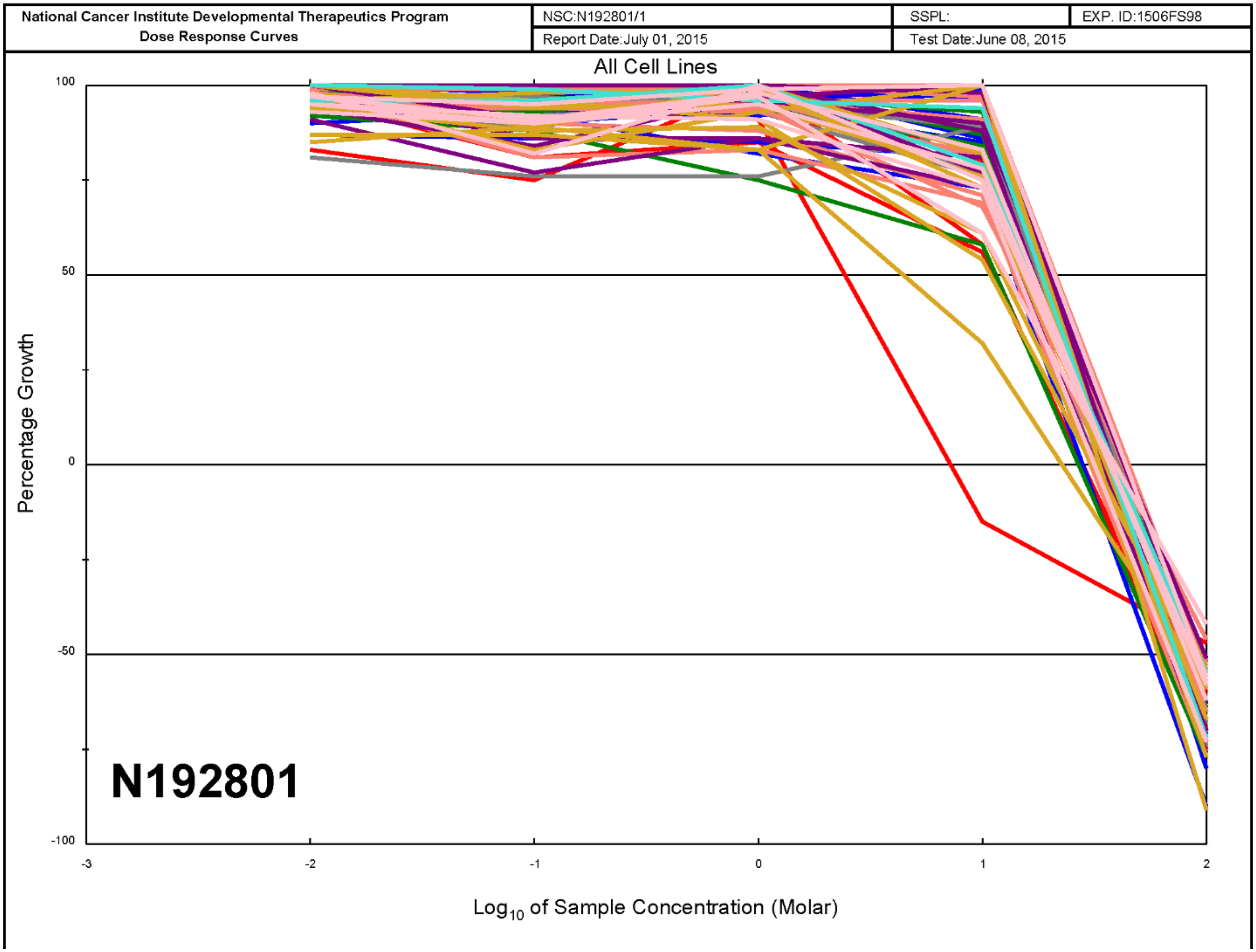

Figure S16. Composite of the NCI-60 dose response curves of the *Rapanea guianensis* root wood ethanol extract (BR 627/N192801) with higher activity against the leukemia SR cell line.

E. *Salacia crassifolia*

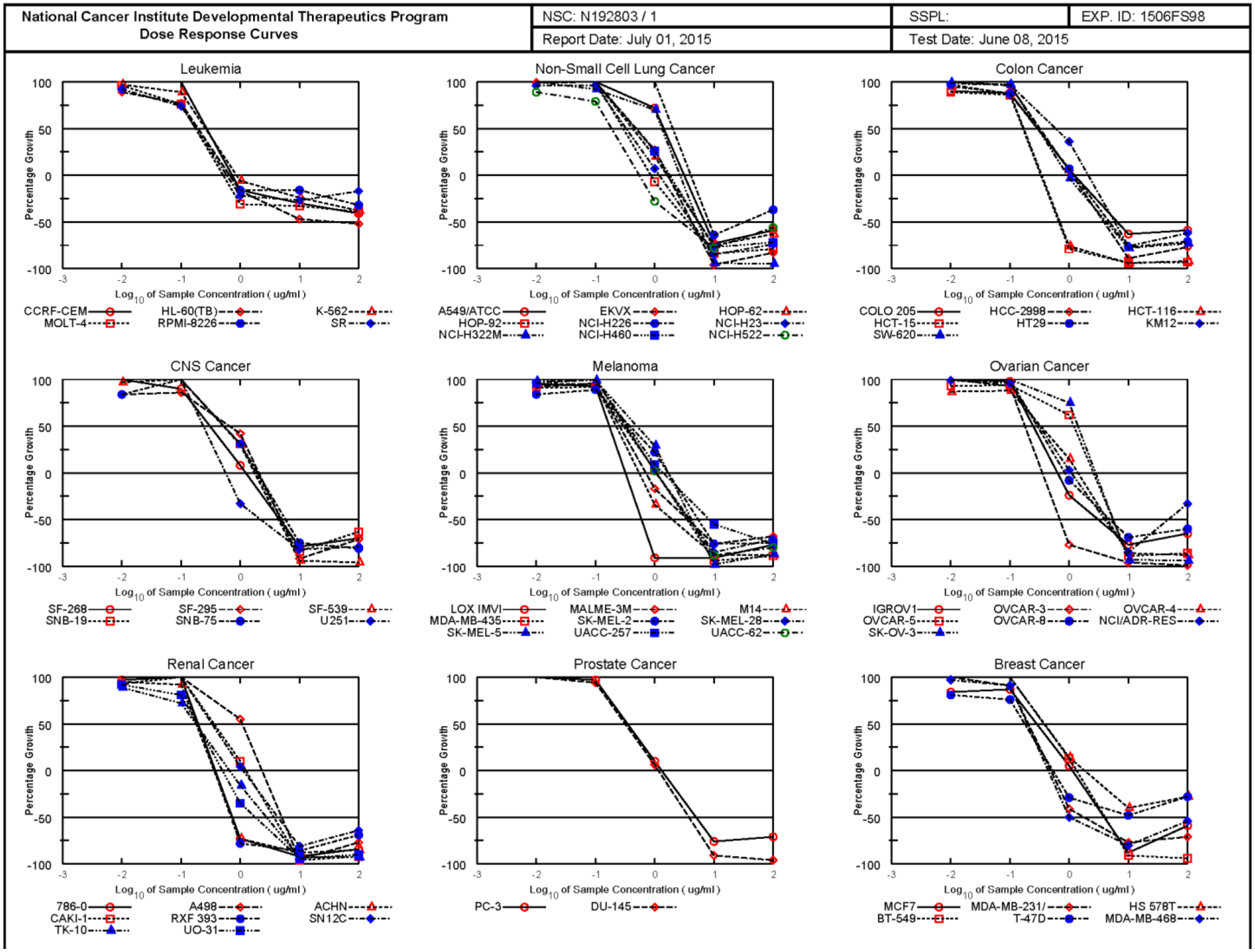

Figure S17. Dose response curves of the *Salacia crassifolia* root wood hexane extract (BR 640/N192803) against NCI-60 panels, with the highest activity against the colon cancer HCT-15 cell line.

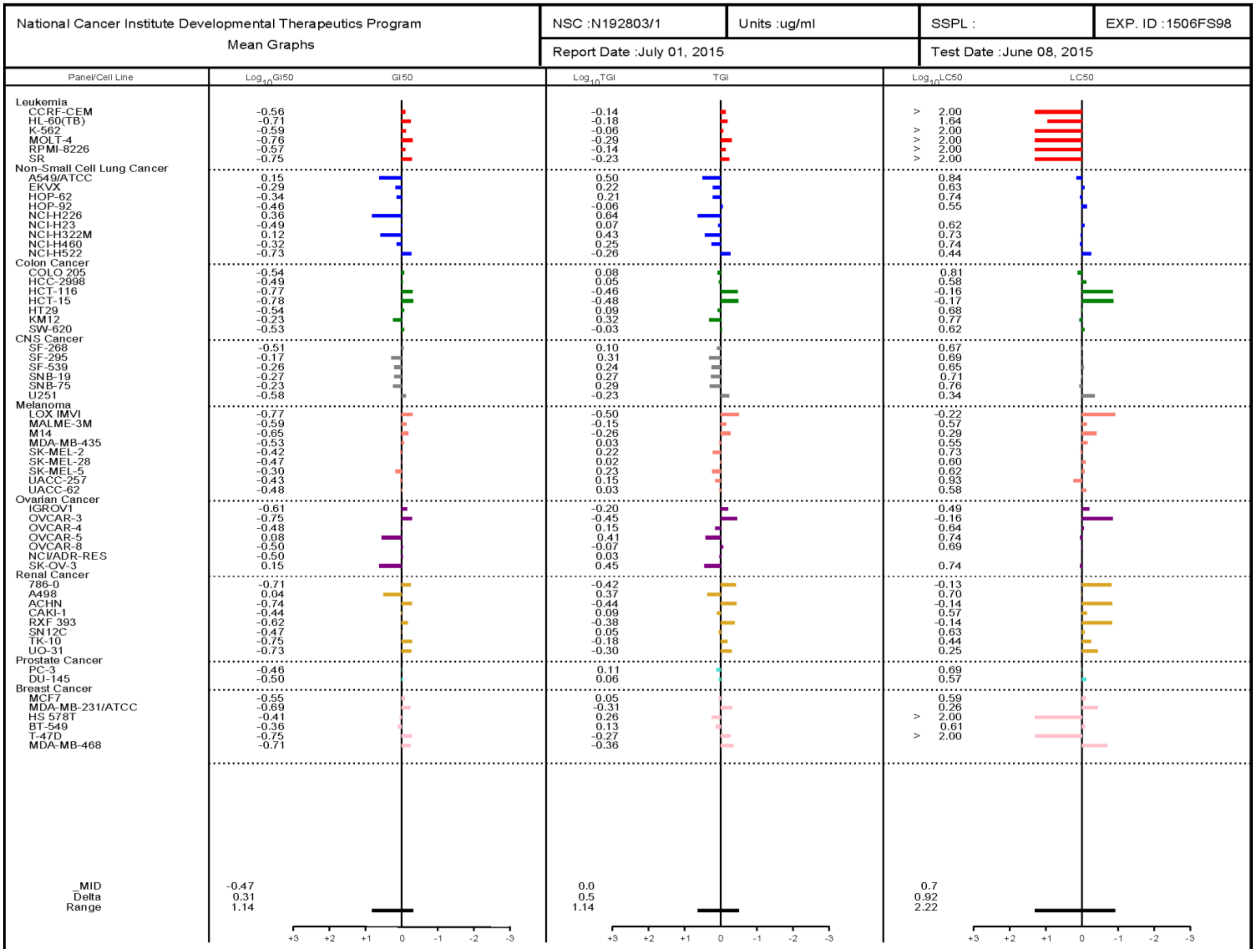

Figure S18. Cytotoxicity of the *Salacia crassifolia* root wood hexane extract (BR 640/N192803) in the NCI-60 cell five-dose screen.

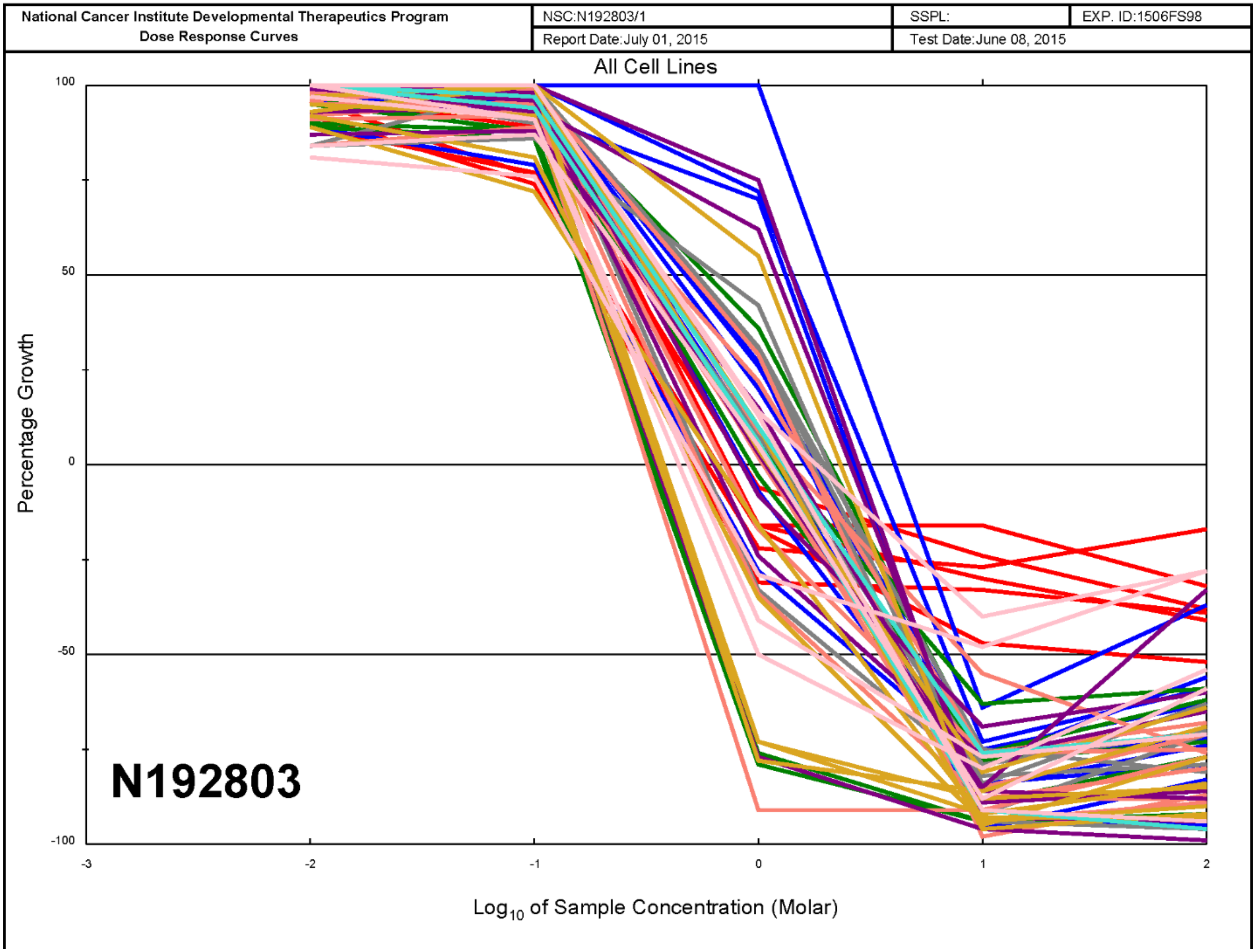

Figure S19. Composite of the NCI-60 dose response curves of the *Salacia crassifolia* root wood hexane extract (BR 640/N192803) with higher activity against the colon cancer HCT-15 cell line.

F. *Salacia elliptica*

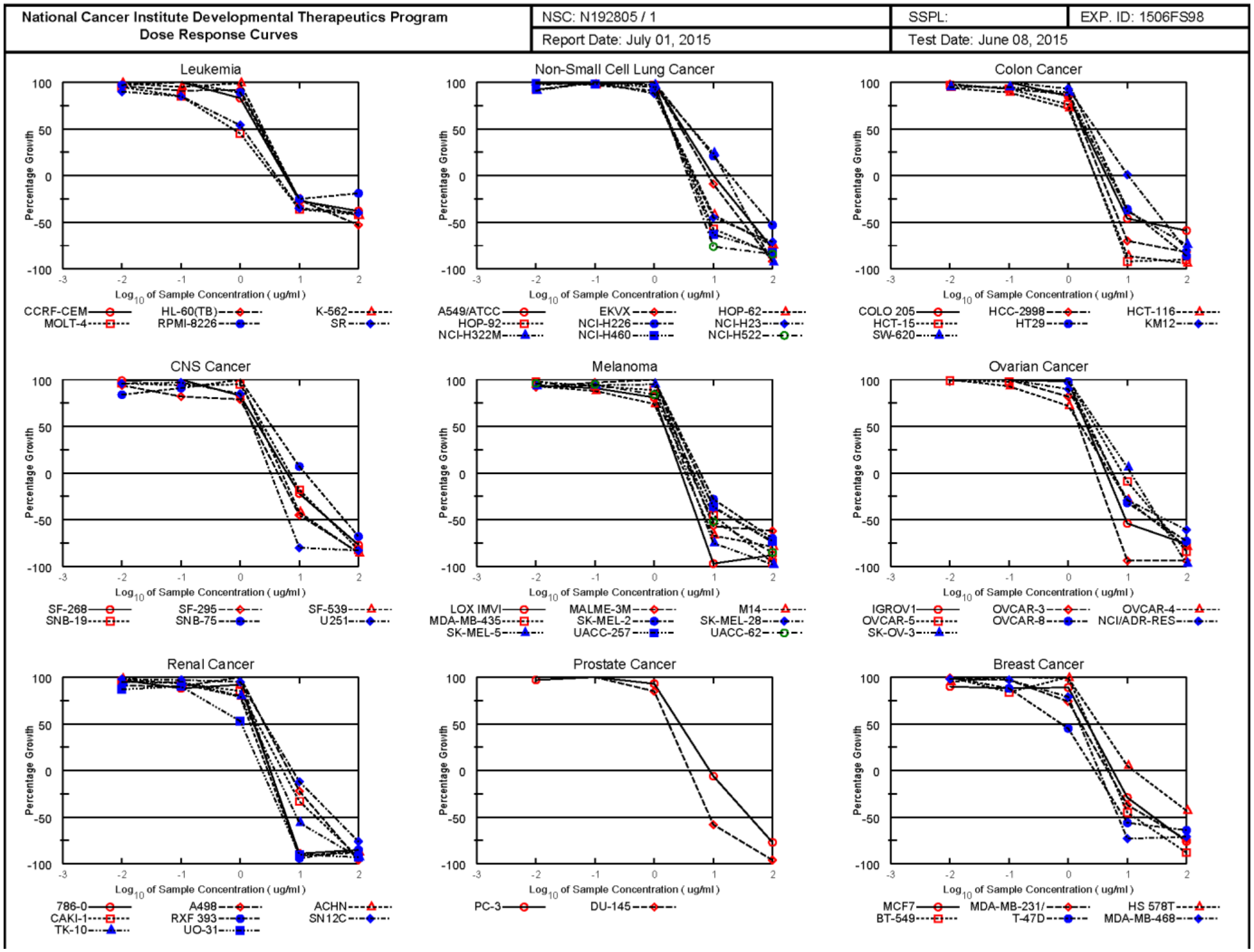

Figure S20. Dose response curves of the *Salacia elliptica* root wood ethyl acetate extract (BR 652/N192805) against NCI-60 panels, with the highest activity against the leukemia MOLT-4 cell line.

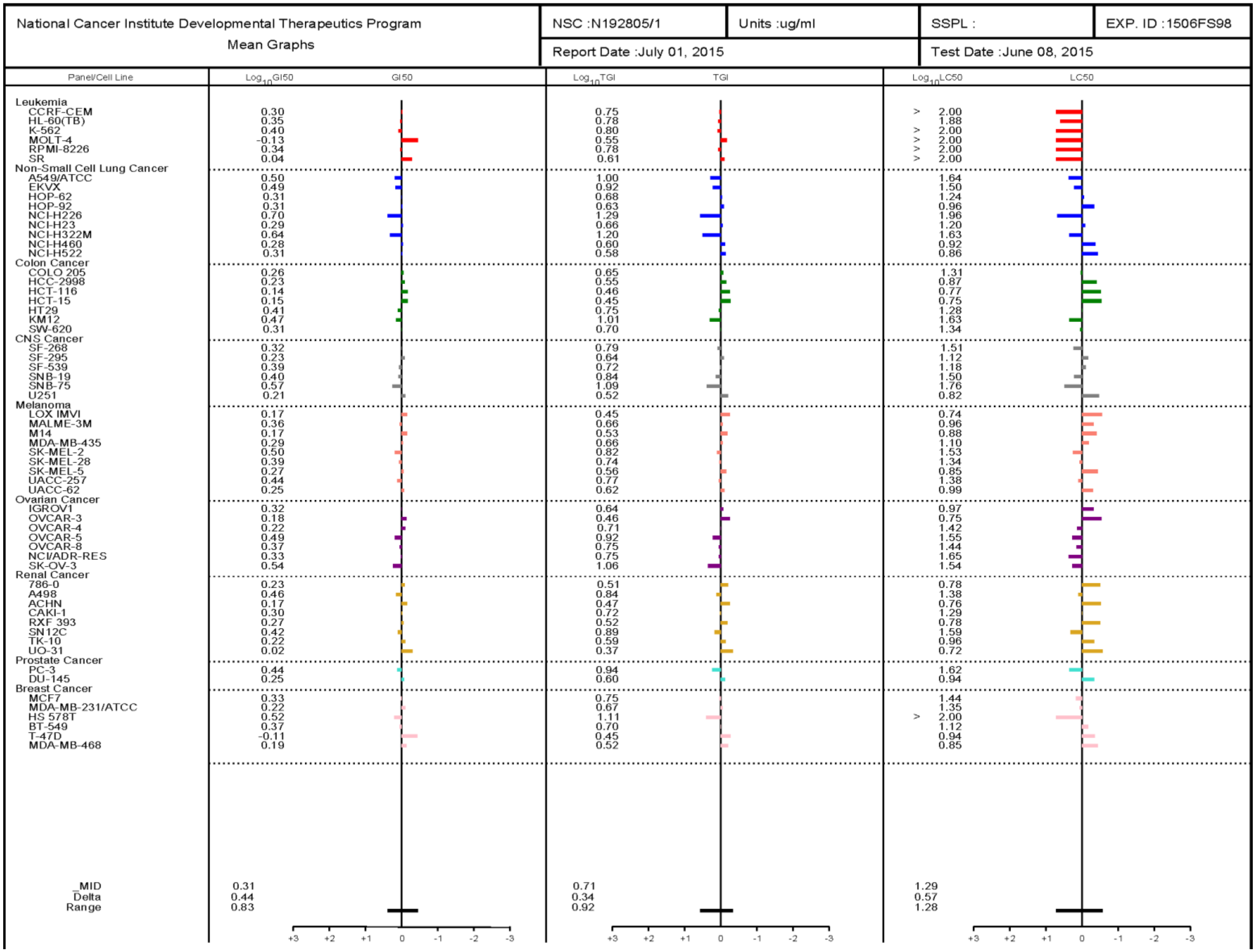

Figure S21. Mean bar graphs of the *Salacia elliptica* root wood ethyl acetate extract (BR 652/N192805) in the NCI-60 cell five-dose screen.

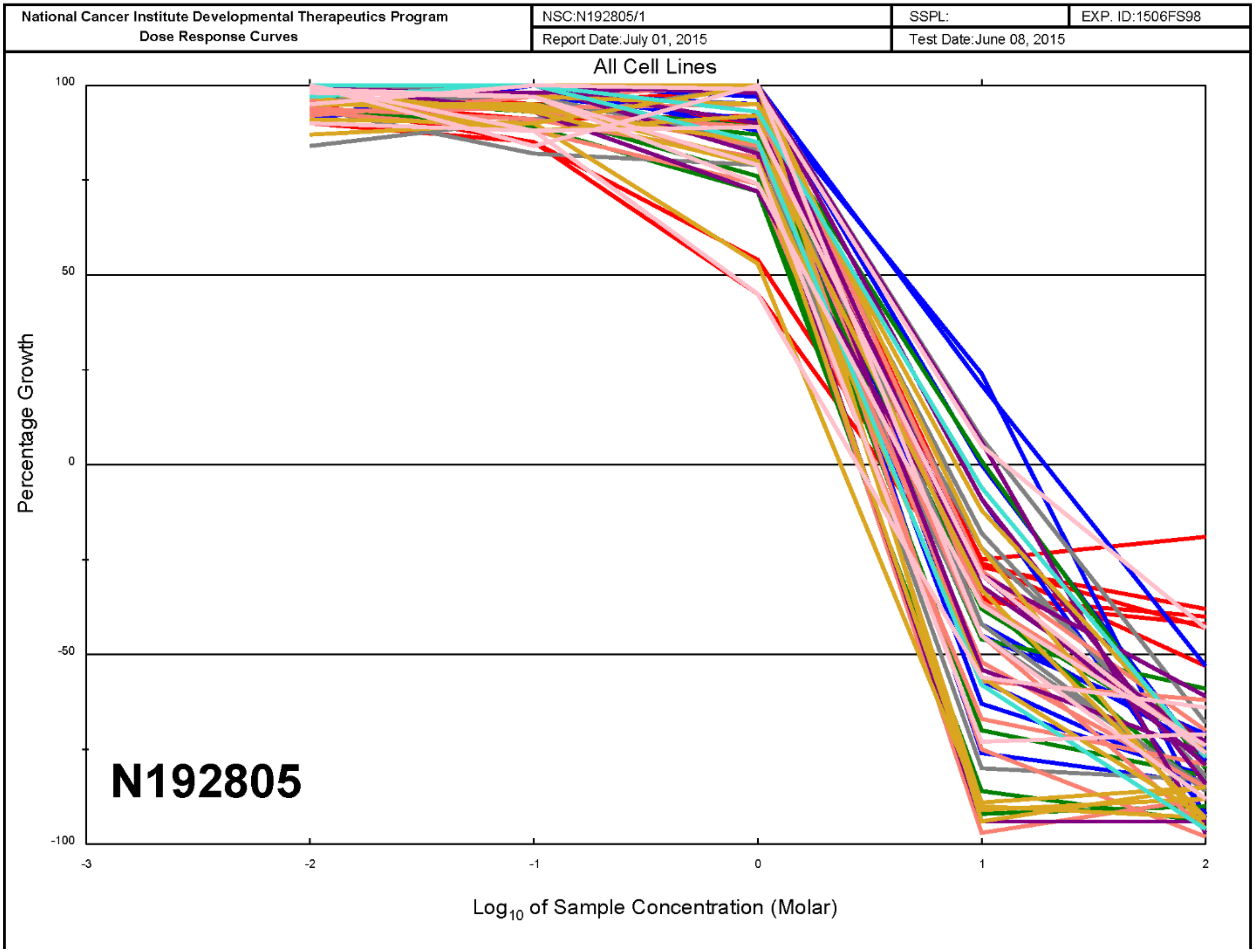

Figure S22. Composite of the NCI-60 dose response curves of the *Salacia elliptica* root wood ethyl acetate extract (BR 652/N192805) with higher activity against the leukemia MOLT-4 cell line.

G. *Casearia sylvestris var. lingua*

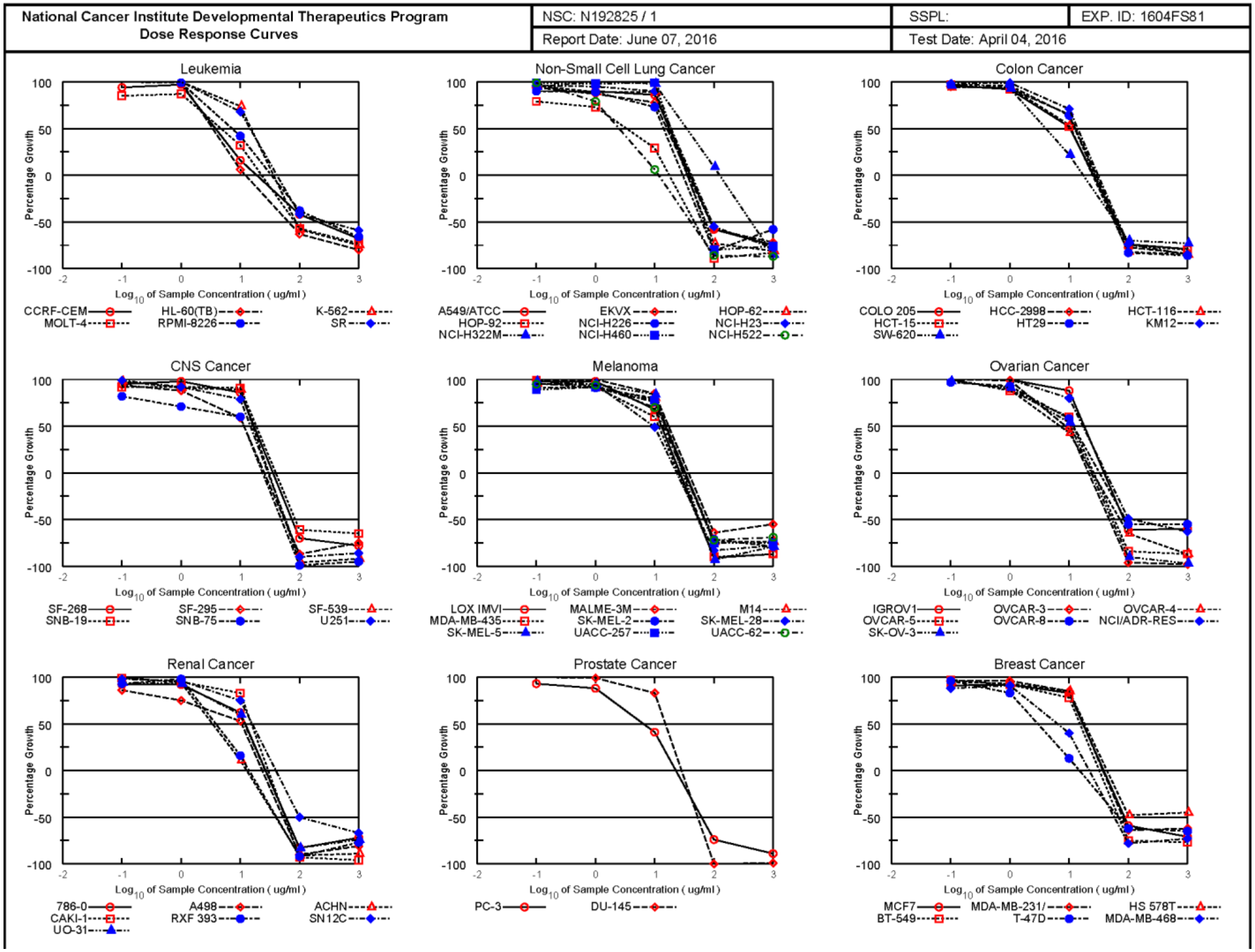

Figure S23. Dose response curves of the *Casearia sylvestris var. lingua* stem wood hexane extract (BR 177/N192825) against NCI-60 panels, with the highest activity against the non-small cell lung cancer NCI-H522.

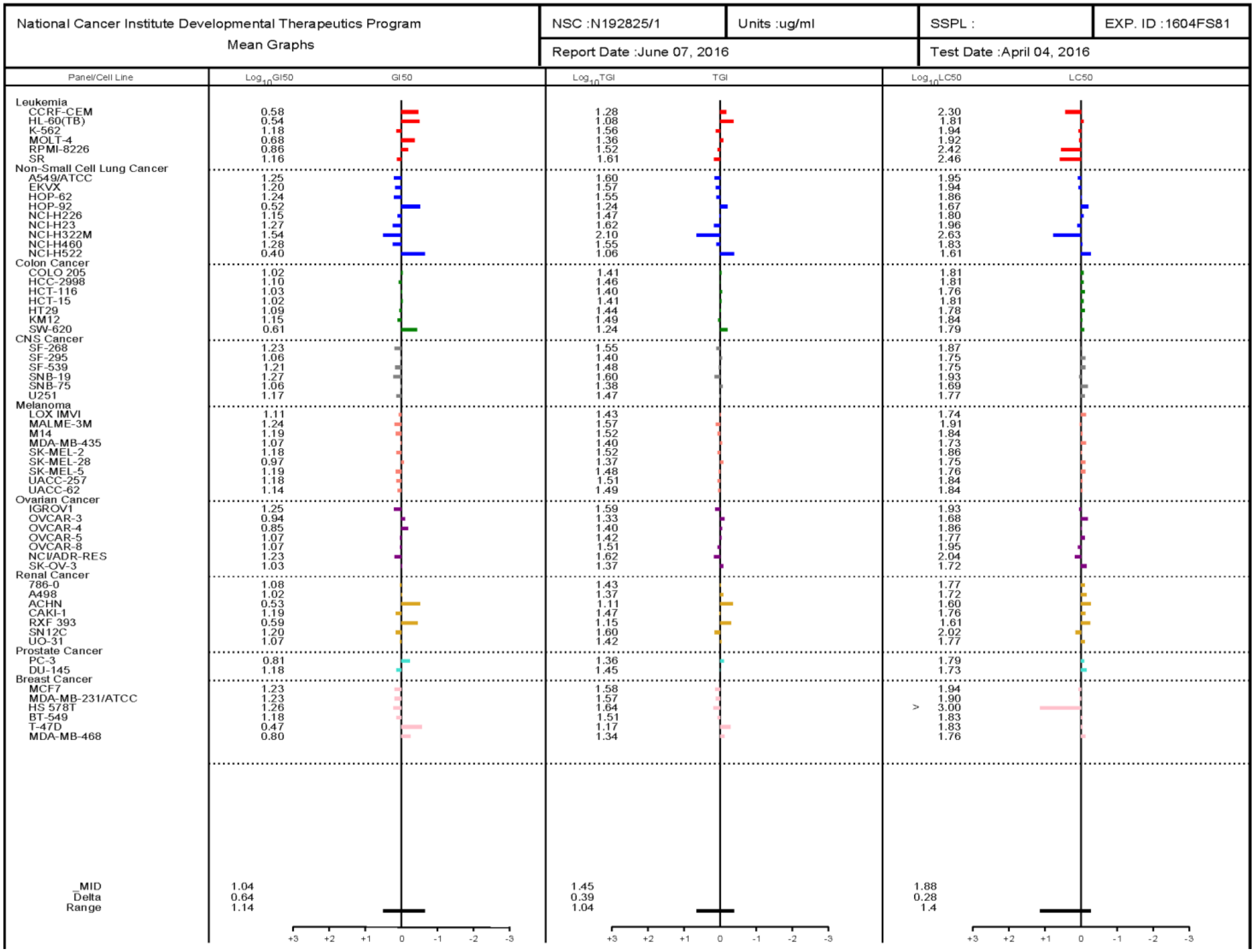

Figure S24. Mean bar graph of the *Casearia sylvestris var. lingua* stem wood hexane extract (BR 177/N192825) in the NCI-60 cell five-dose screen.

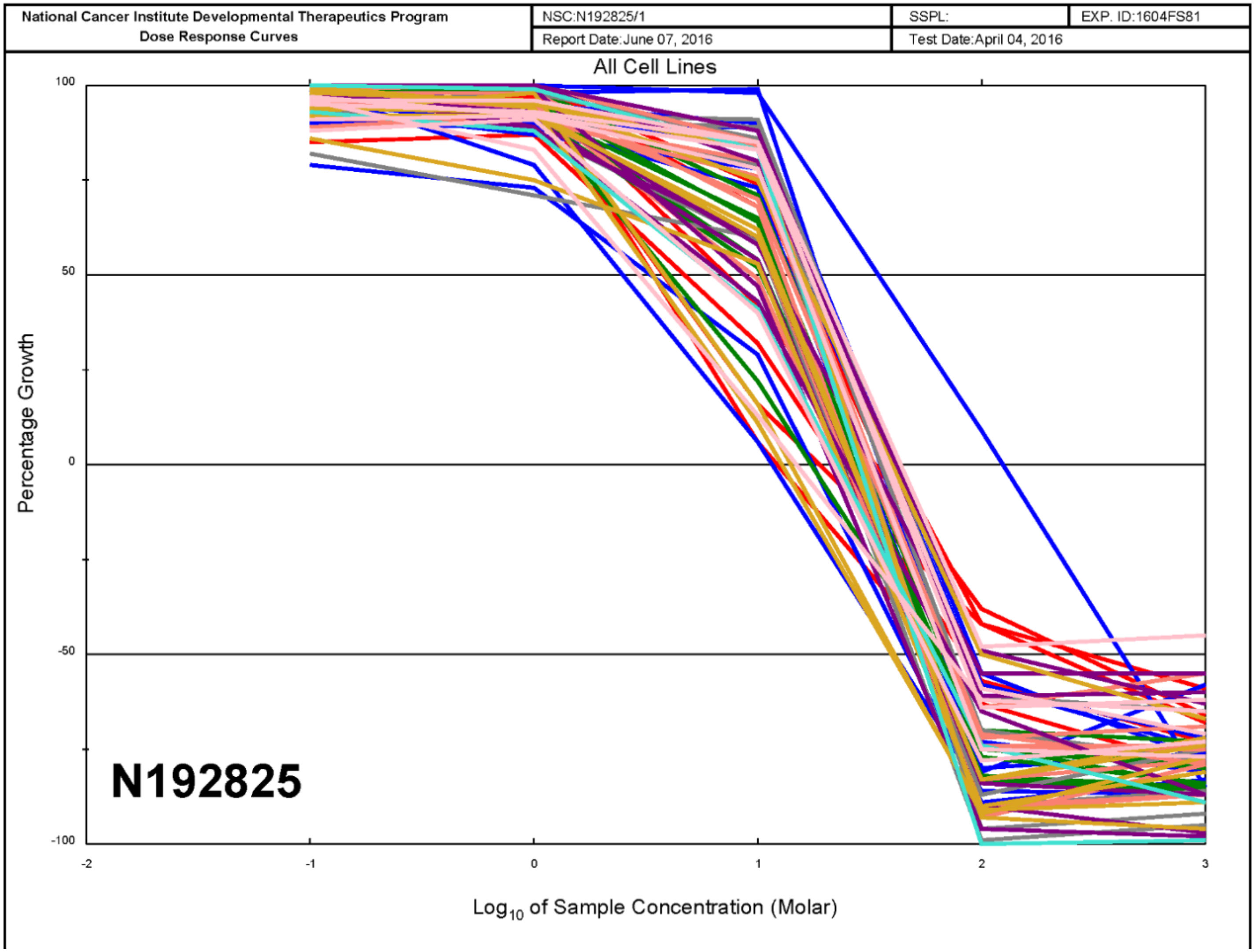

Figure S25. Composite of the NCI-60 dose response curves of the *Casearia sylvestris var. lingua* stem wood hexane extract (BR 177/N192825) with higher activity against the non-small cell lung cancer NCI-H522.

H. Cupania vernalis

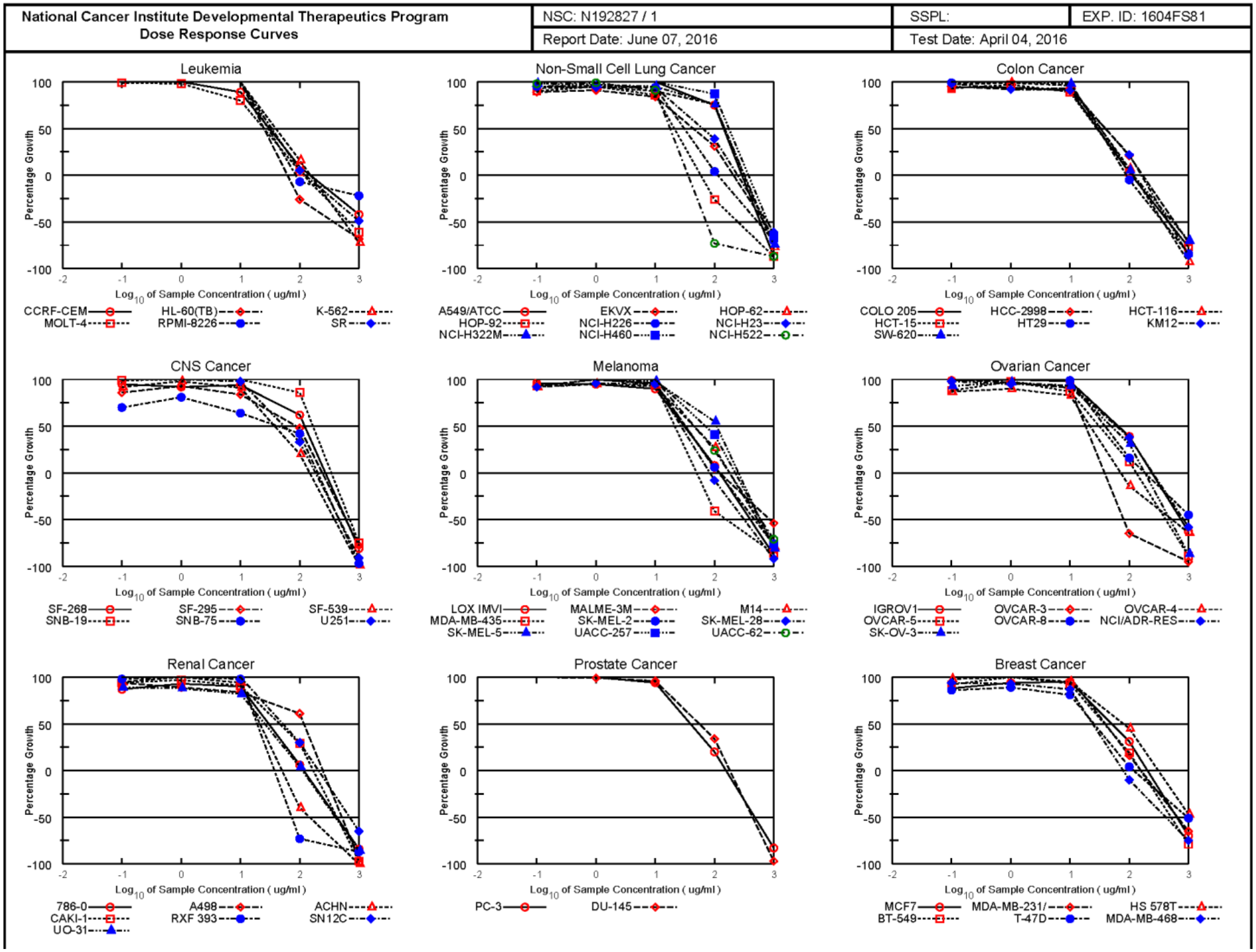

Figure S26. Dose response curves of the *Cupania vernalis* leaf hexane extract (BR 193/N192827) against NCI-60 panels, with different susceptibility.

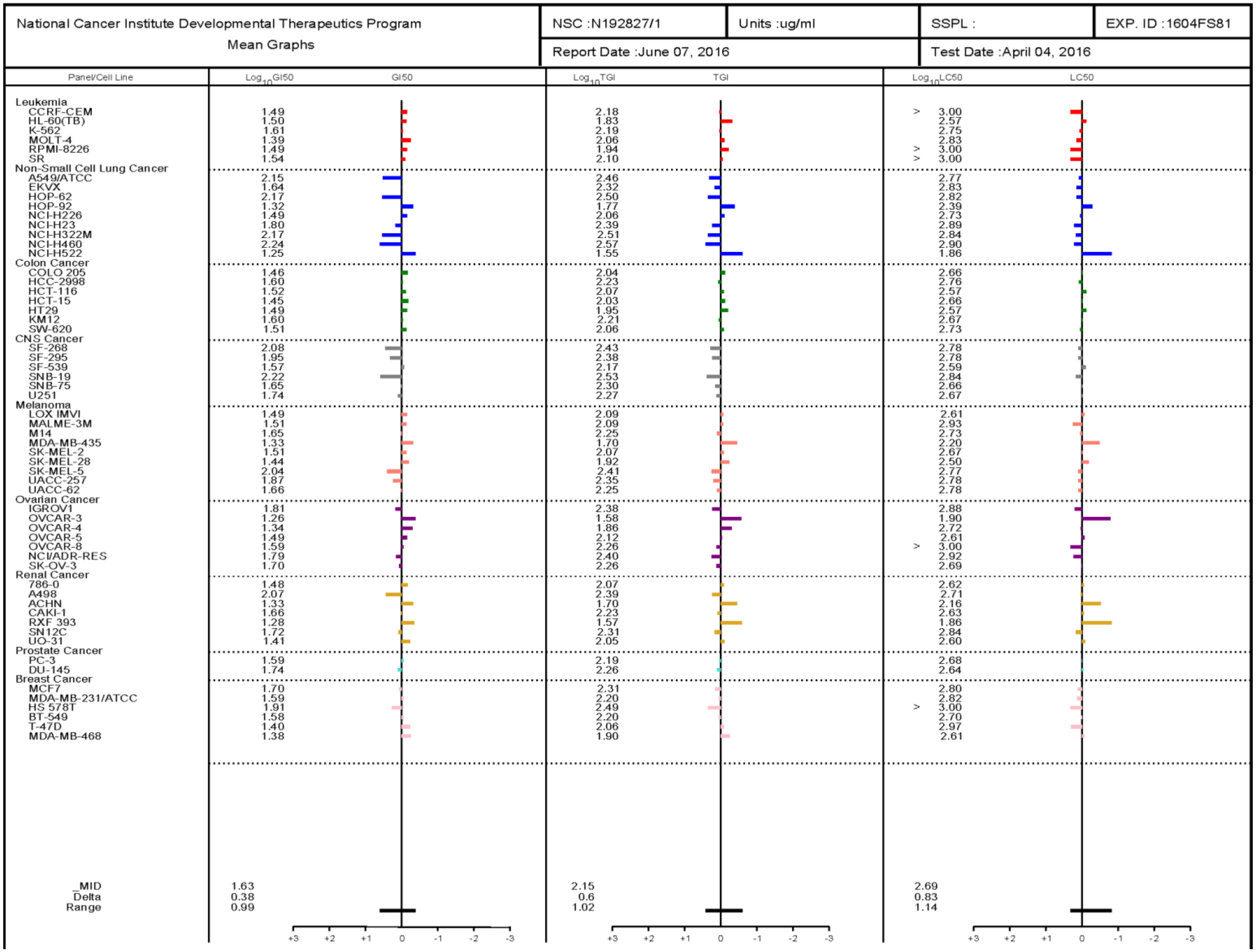

Figure S27. Mean bar graph of the *Cupania vernalis* leaf hexane extract (BR 193/N192827) in the NCI-60 cell five-dose screen.

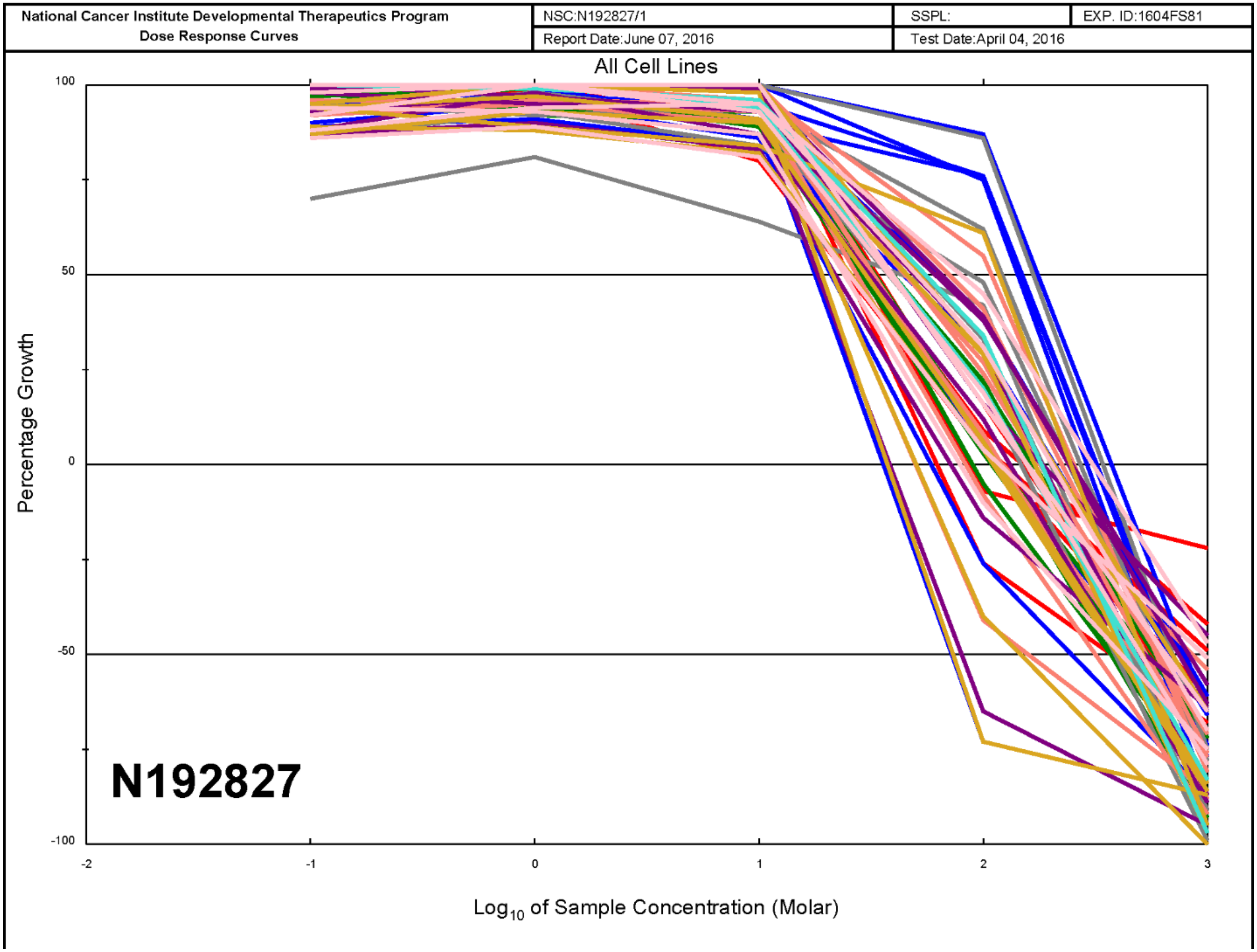

Figure S28. Composite of the NCI-60 dose response curves of the *Cupania vernalis* leaf hexane extract (BR 193/N192827).

I. Simarouba versicolor

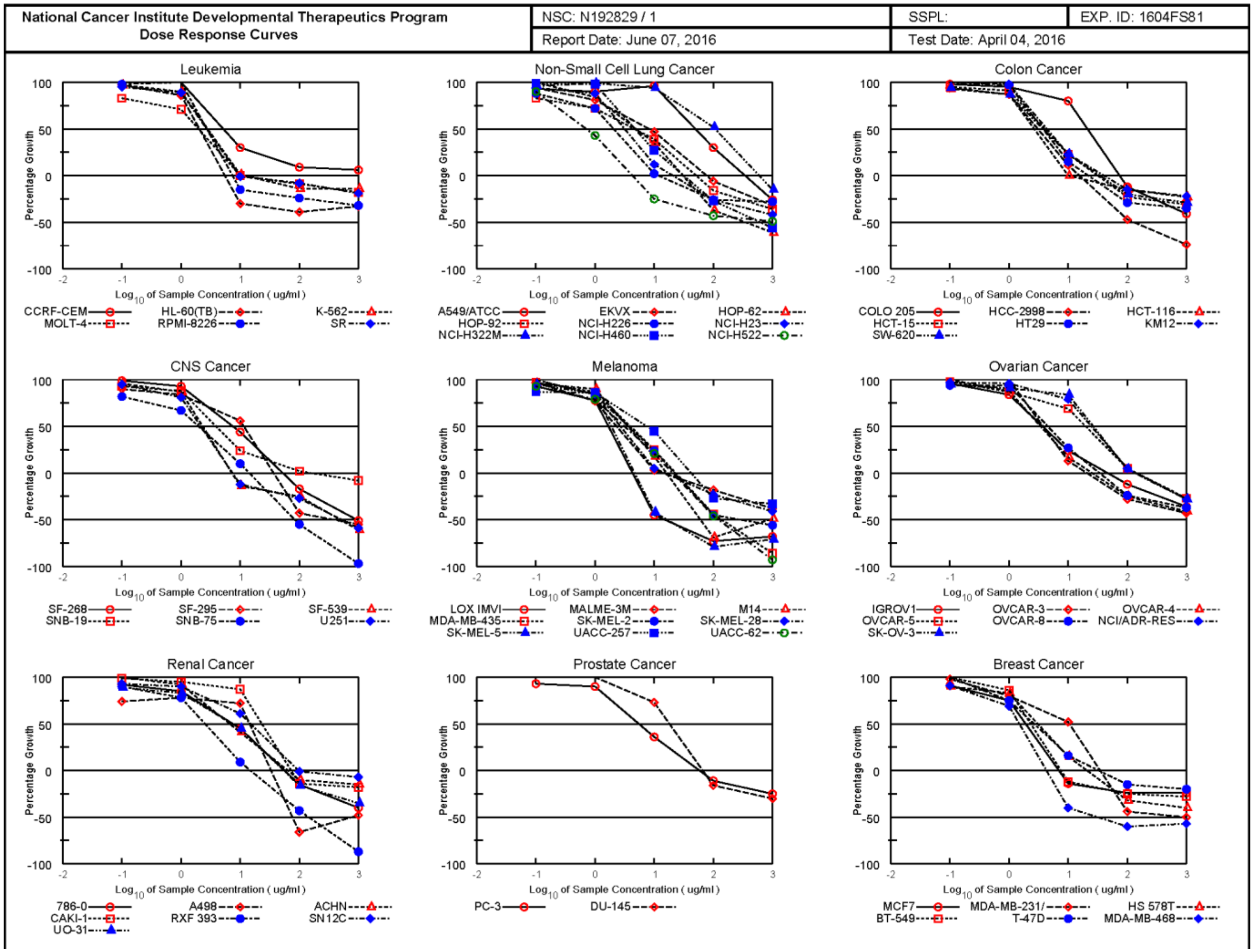

Figure S29. Dose response curves of the *Simarouba versicolor* root bark ethanol extract (BR 254/N192829) against NCI-60 panels, with the highest activity against the non-small cell lung cancer NCI-H522.

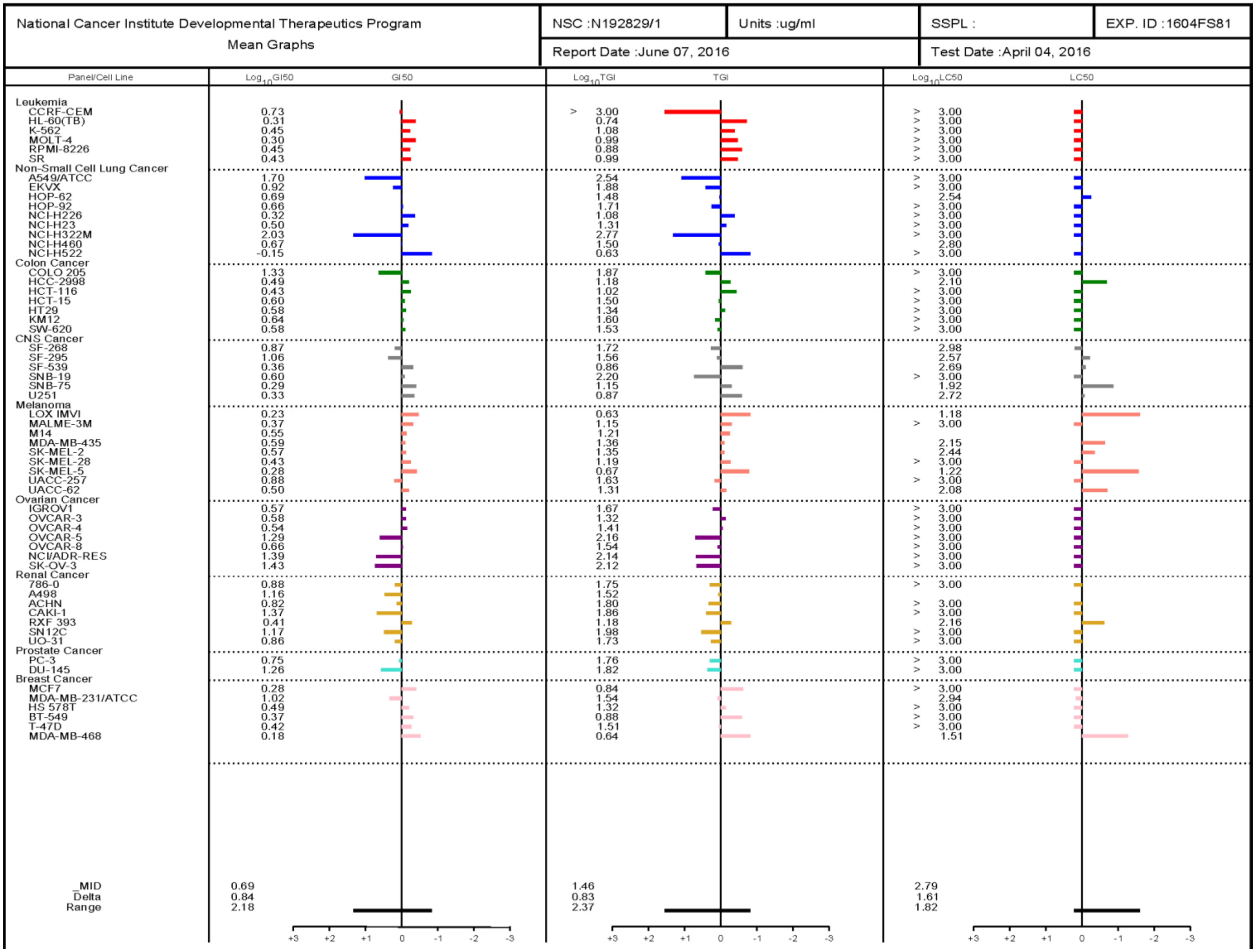

Figure S30. Mean bar graph of the *Simarouba versicolor* root bark ethanol extract (BR 254/N192829) in the NCI-60 cell five-dose screen.

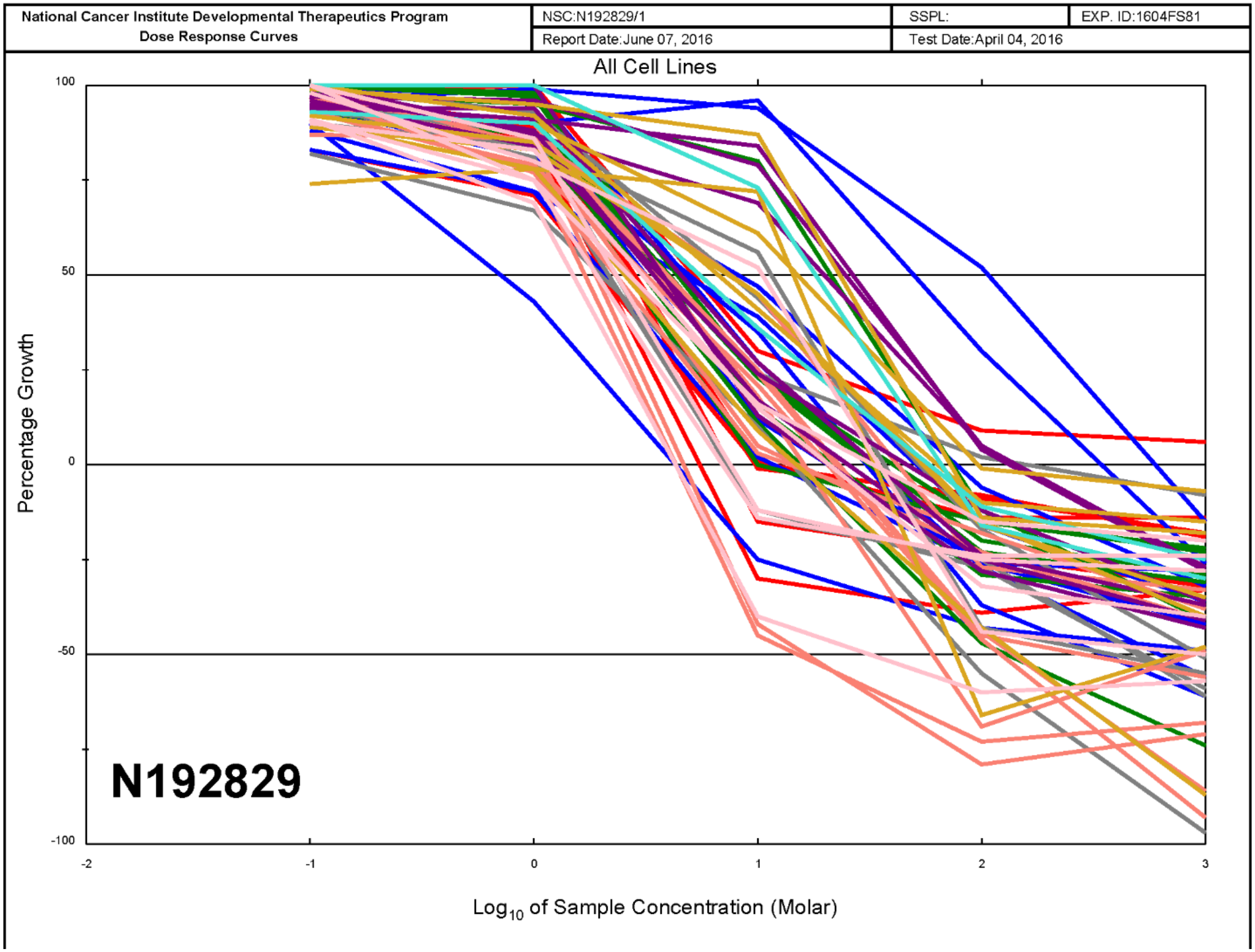

Figure S31. NCI-60 dose response curves of the *Simarouba versicolor* root bark ethanol extract (BR 254/N192829) with higher activity against the non-small cell lung cancer NCI-H522.

J. *Kielmeyera coriacea*

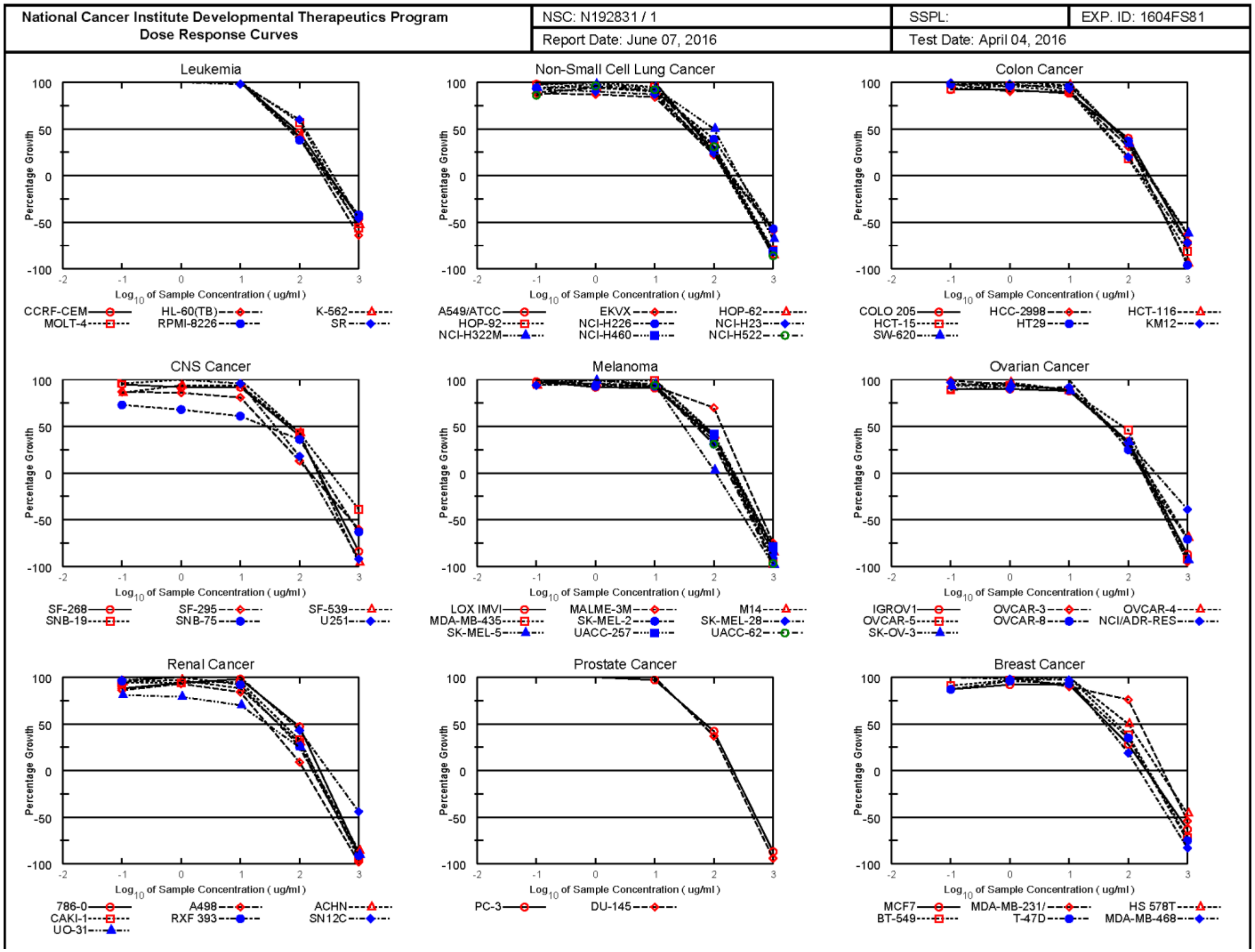

Figure S32. Dose response curves of the *Kielmeyera coriacea* stem wood hexane extract (BR 331/N192831) against NCI-60 panels, with different susceptibility.

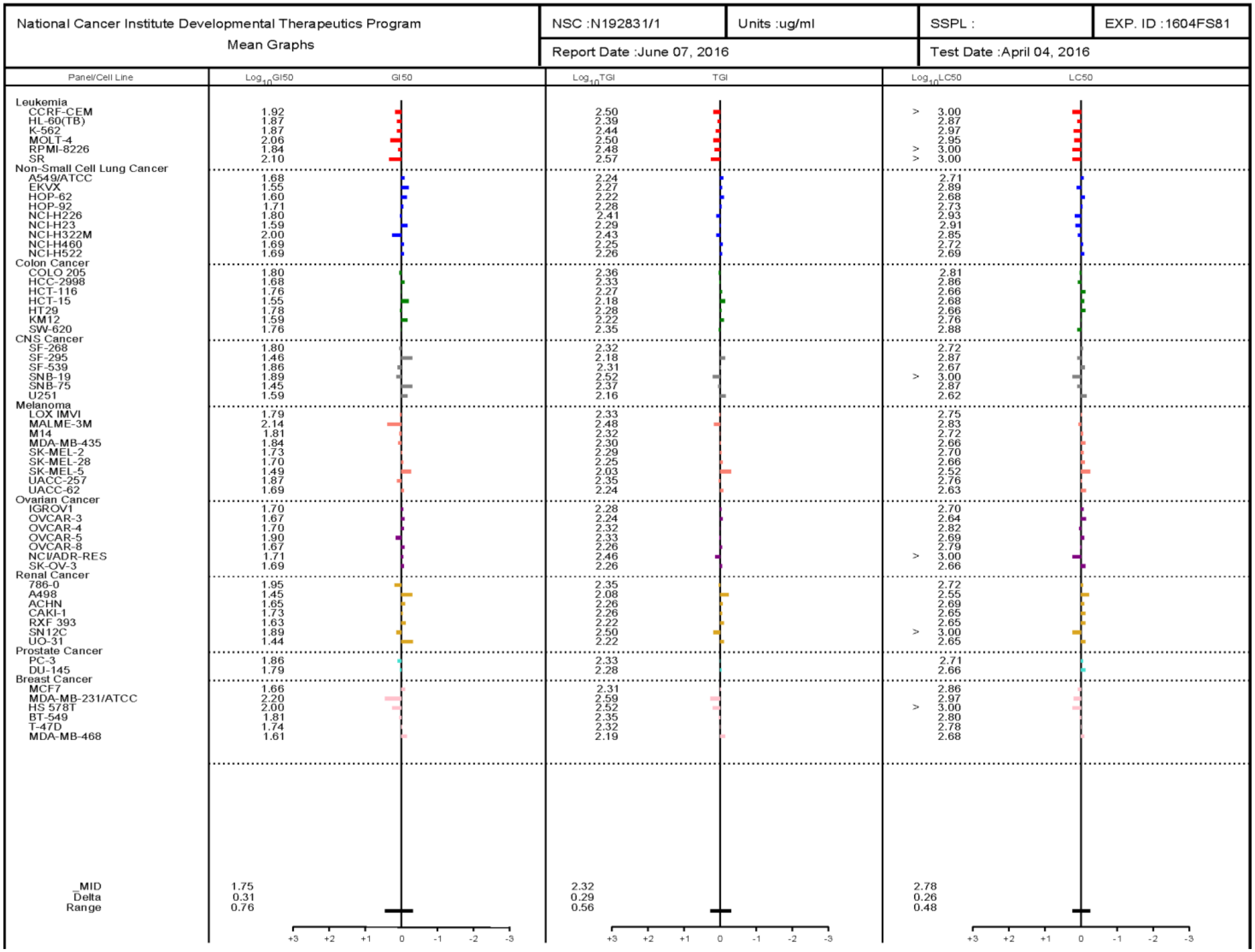

Figure S33. Mean bar graph of the *Kielmeyera coriacea* stem wood hexane extract (BR 331/N192831) in the NCI-60 cell five-dose screen.

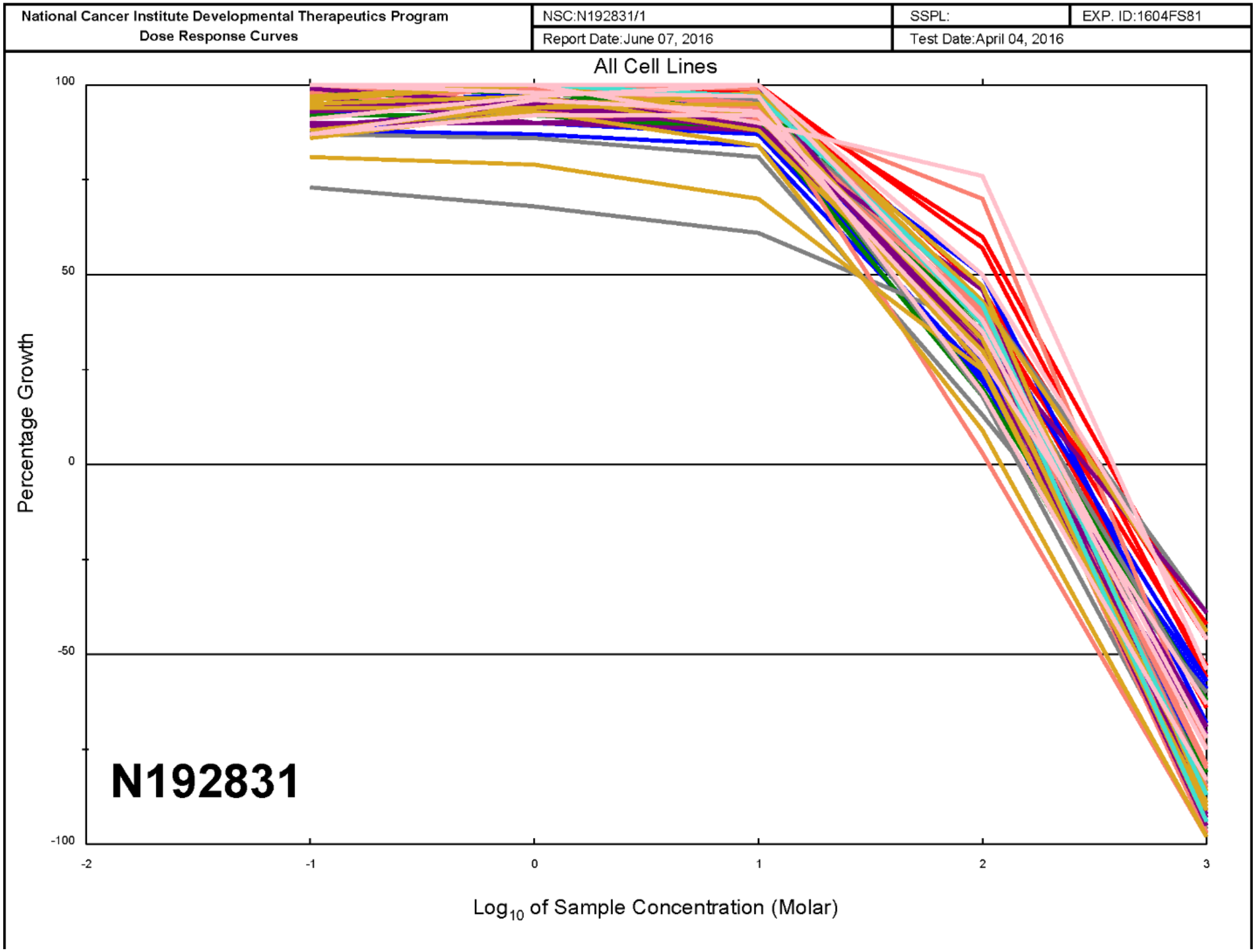

Figure S34. NCI-60 dose response curves of the *Kielmeyera coriacea* stem wood hexane extract (BR 331/N192831).

K. *Byrsonima crassa*

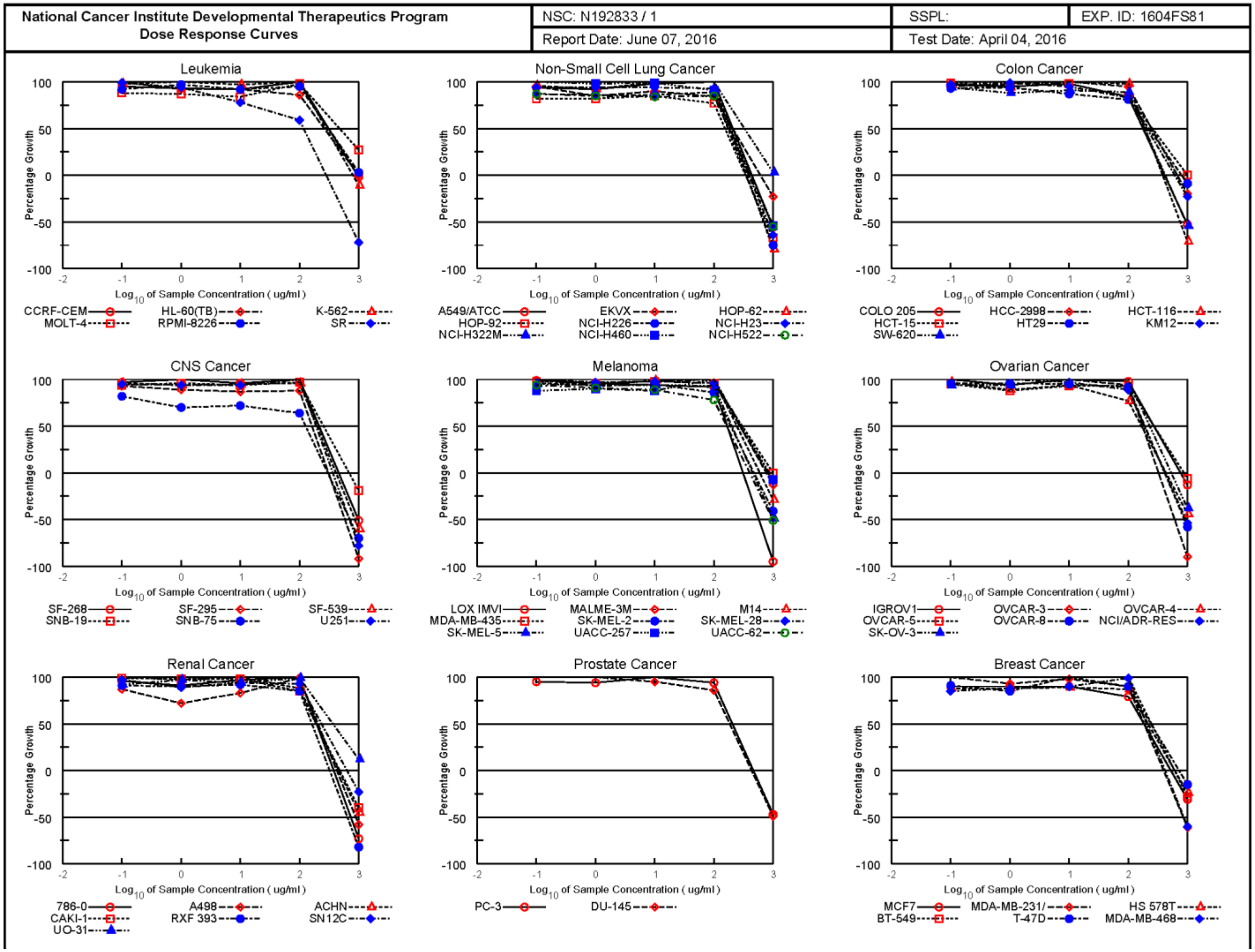

Figure S35. Dose response curves of the *Byrsonima crassa* root bark hexane extract (BR 411/N192833) against NCI-60 panels, with different susceptibility.

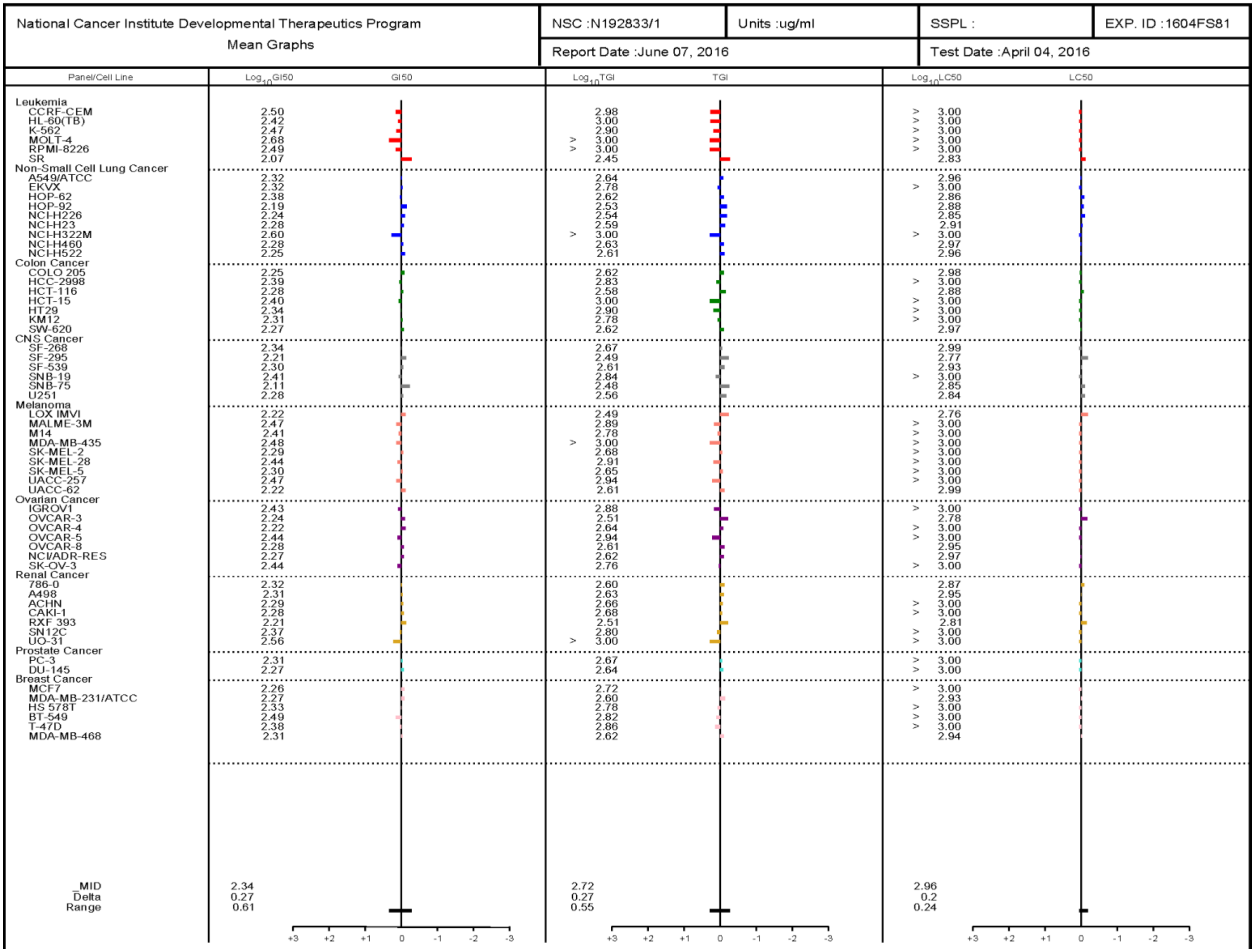

Figure S36. Mean bar graph of the *Byrsonima crassa* root bark hexane extract (BR 411/N192833) in the NCI-60 cell five-dose screen.

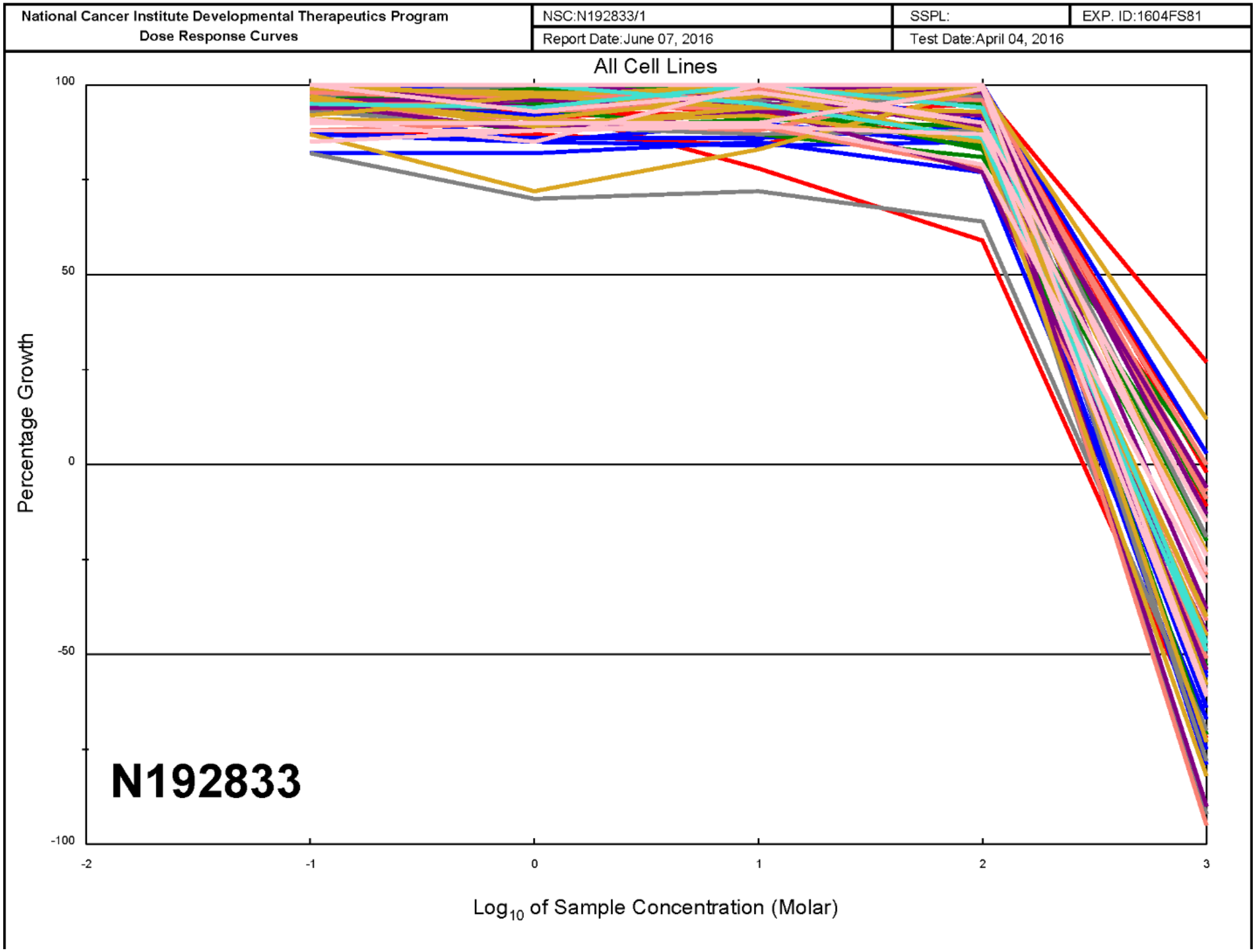

Figure S37. Composite of the NCI-60 dose response curves of the *Byrsonima crassa* root bark hexane extract (BR 411/N192833).

L. *Schinus terebinthifolia*

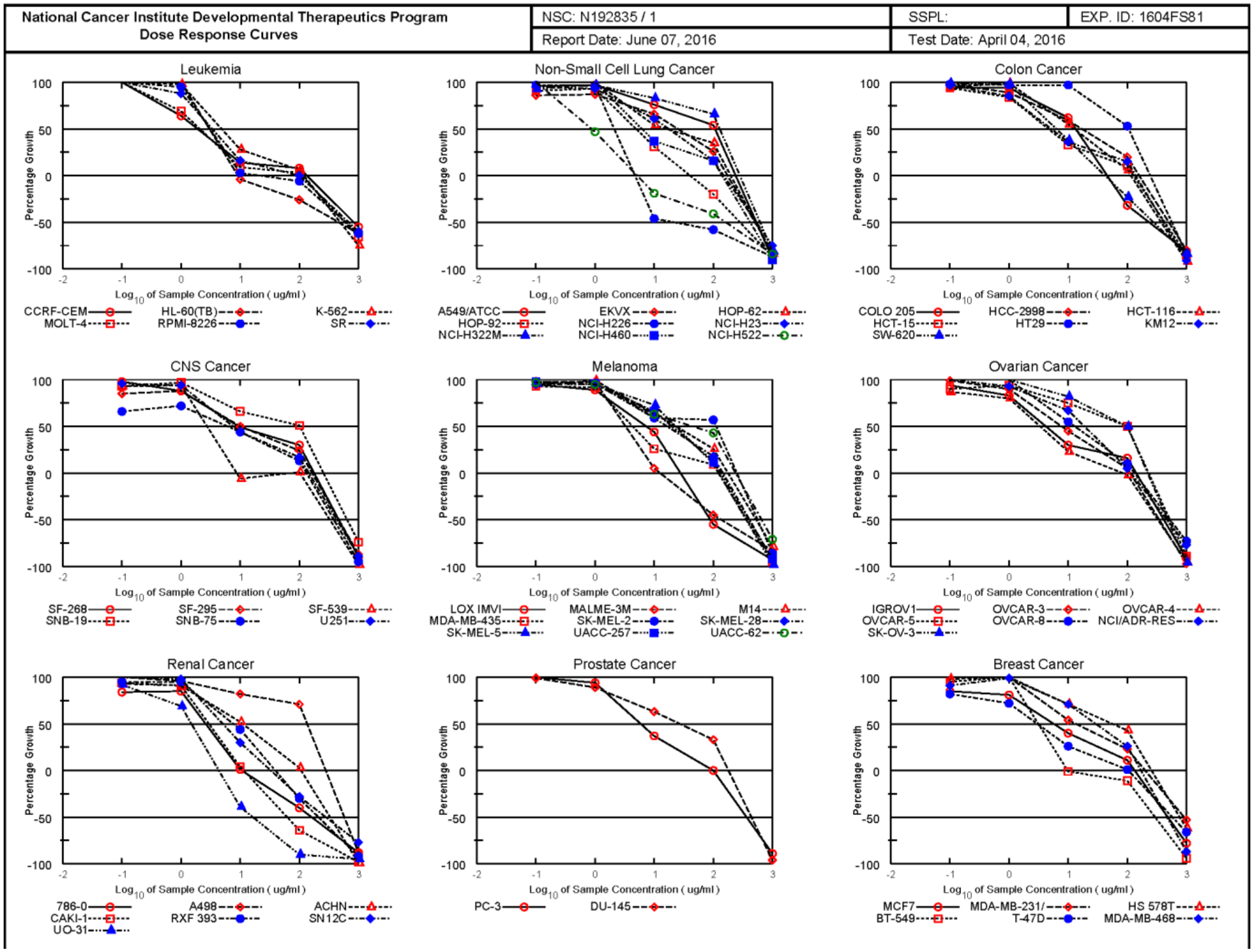

Figure S38. Dose response curves of the *Schinus terebinthifolia* leaf dichloromethane extract (BR 436/N192835) against NCI-60 panels, with the highest activity against the non-small cell lung cancer NCI-H522.

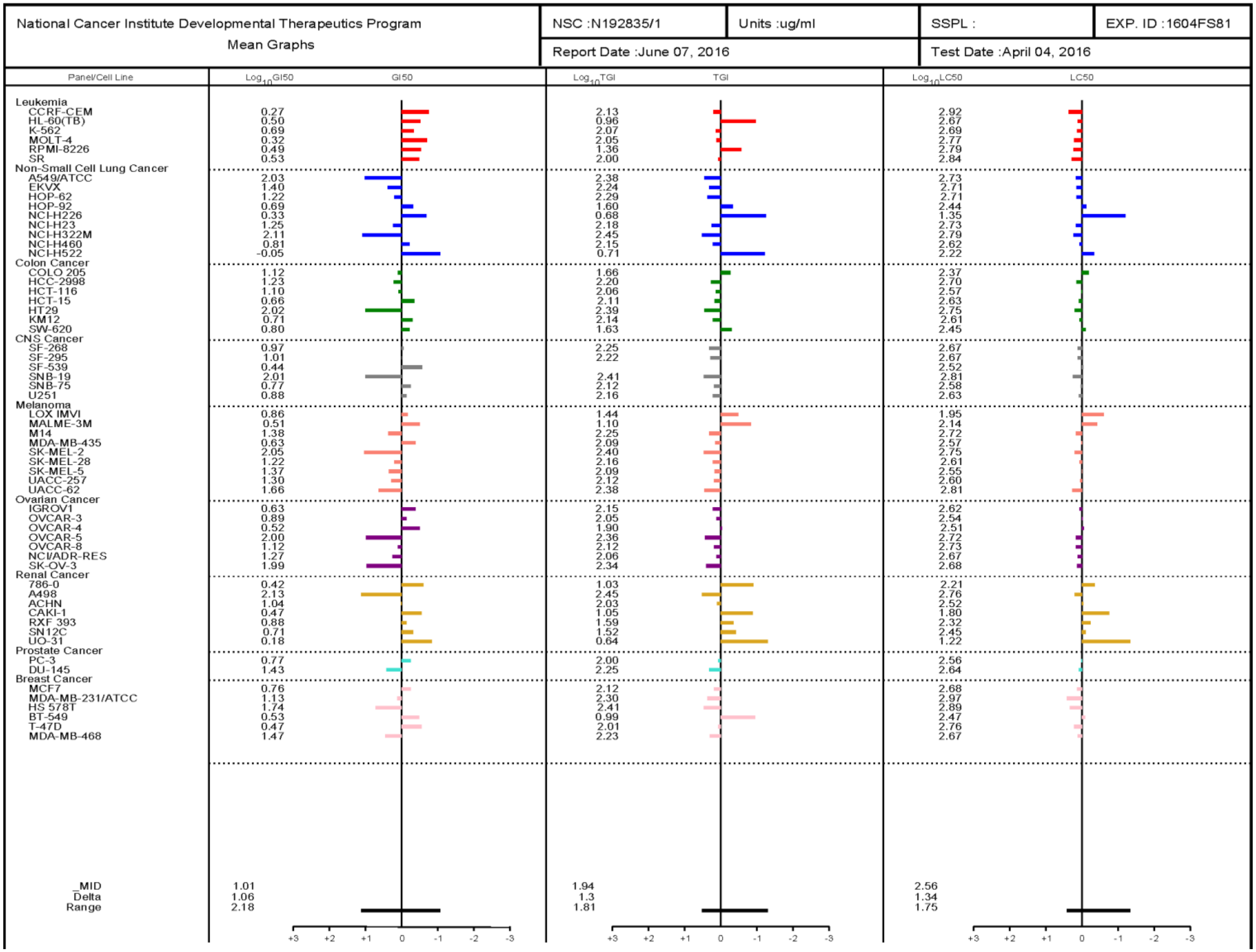

Figure S39. Mean bar graph of the *Schinus terebinthifolia* leaf dichloromethane extract (BR 436/N192835) in the NCI-60 cell five-dose screen.

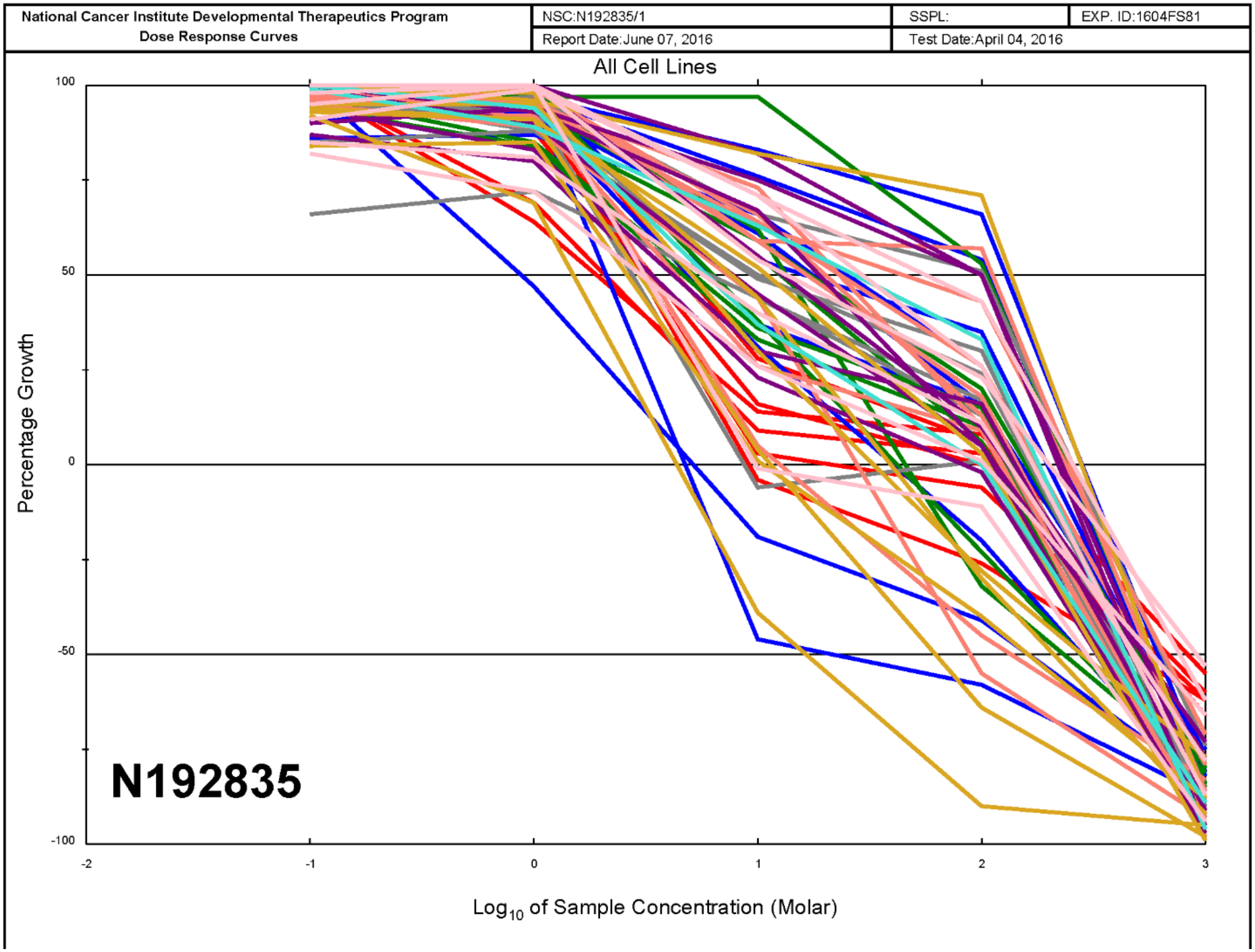

Figure S40. Composite of the NCI-60 dose response curves of the *Schinus terebinthifolia* leaf dichloromethane extract (BR 436/N192835) with higher activity against the non-small cell lung cancer NCI-H522.

M. *Enterolobium gummiferum*

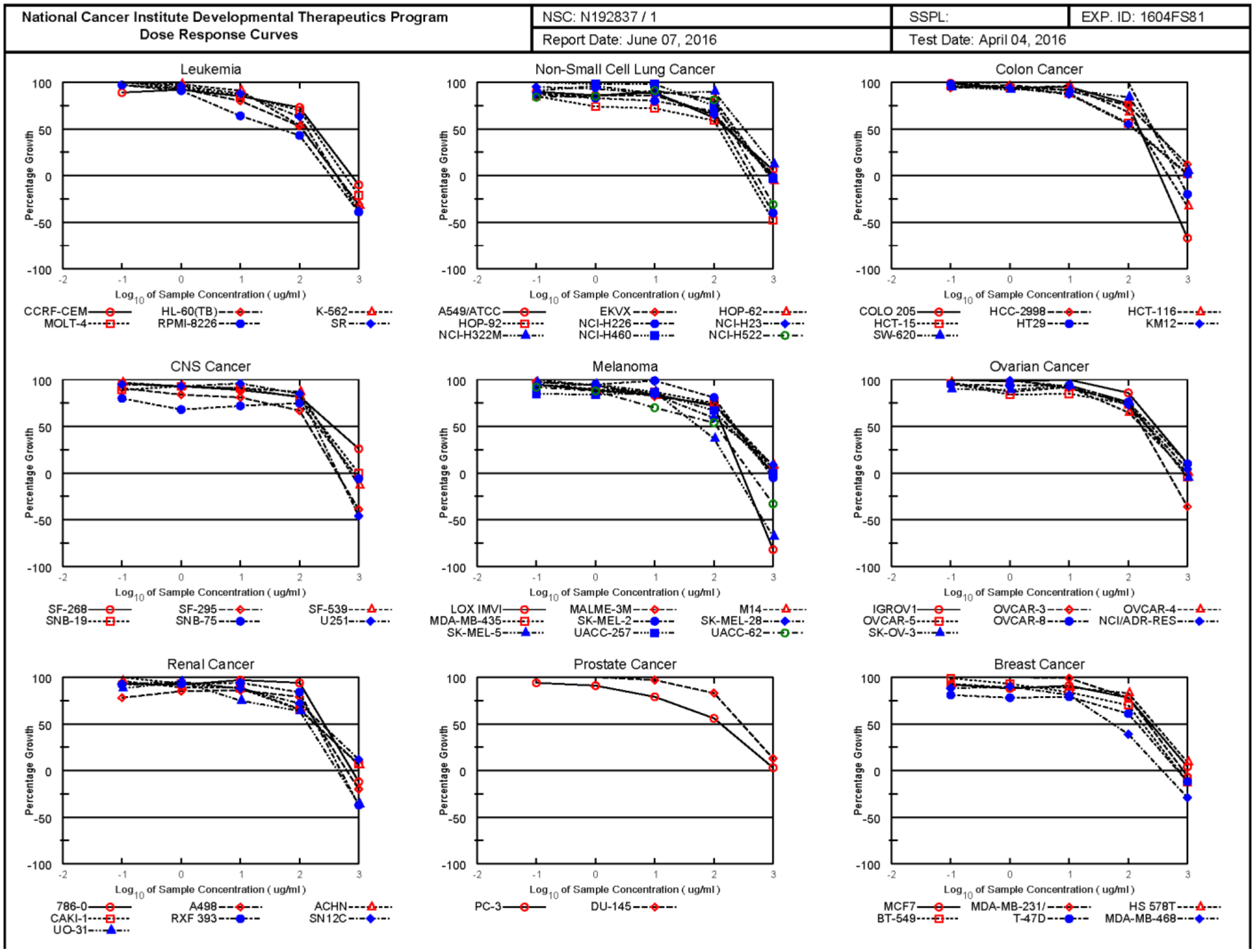

Figure S41. Dose response curves of the *Enterolobium gummiferum* stem bark hexane extract (BR 469/N192837) against 9 cell panels with different susceptibility.

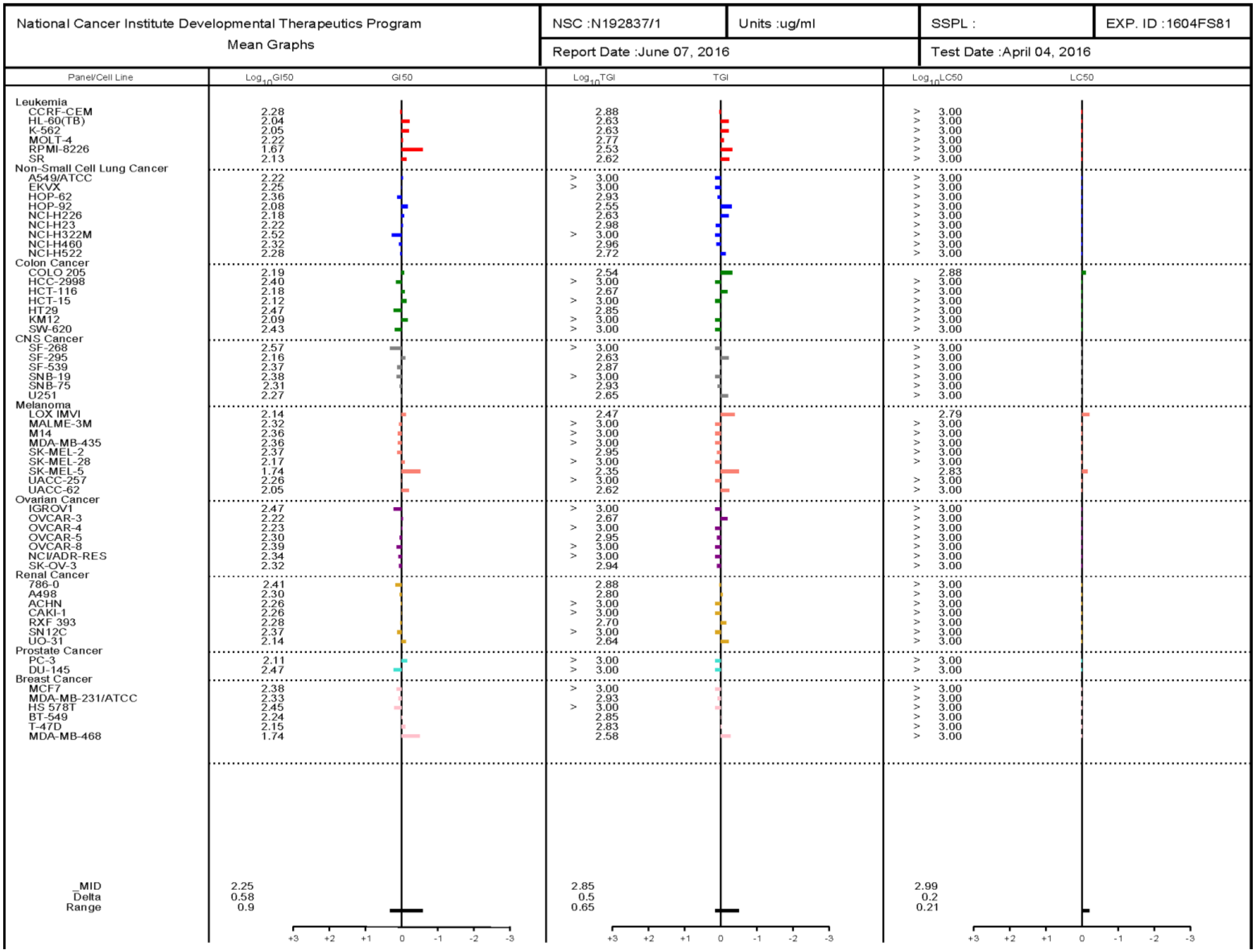

Figure S42. Mean bar graph of the *Enterolobium gummiferum* stem bark hexane extract (BR 469/N192837) in the NCI-60 cell five-dose screen.

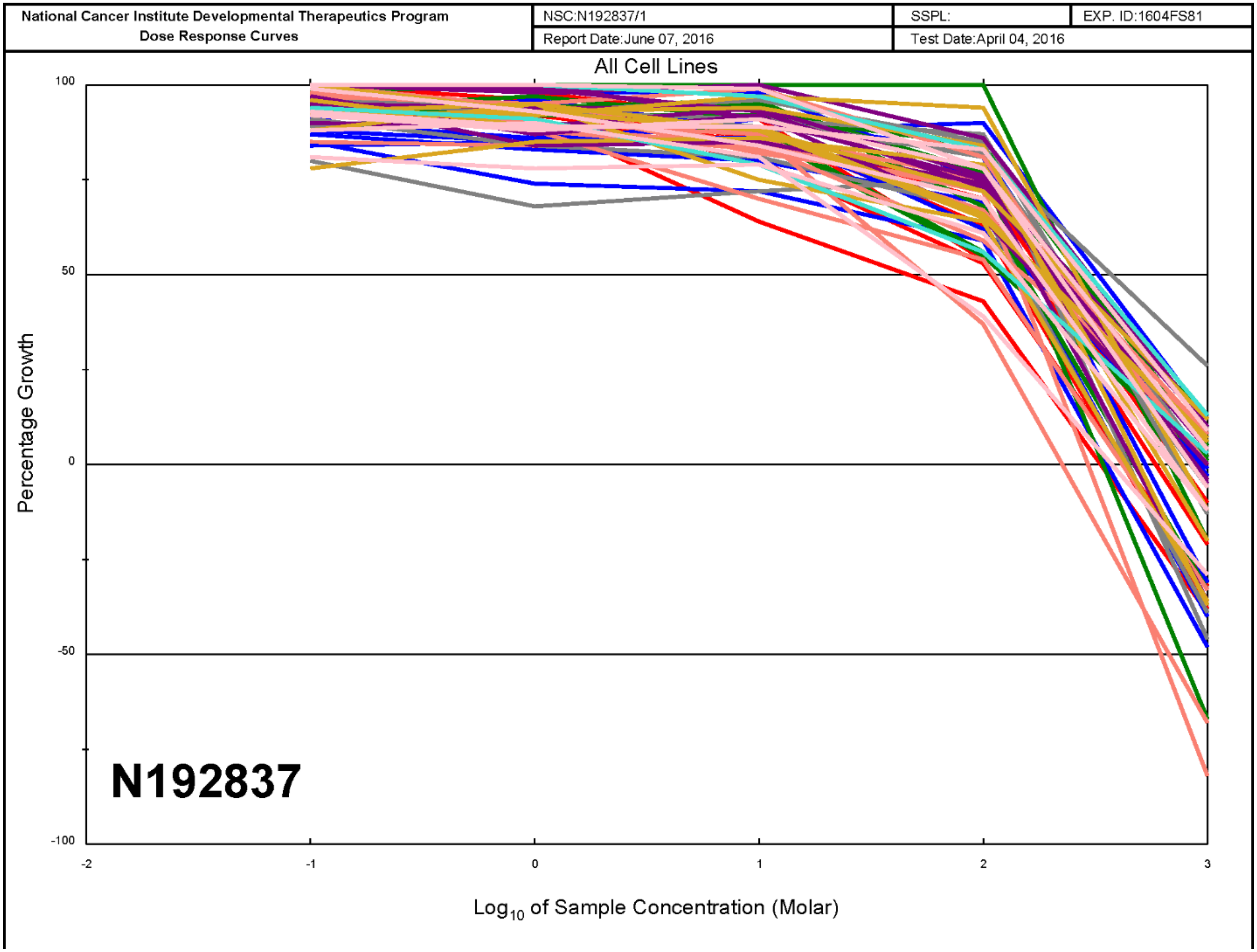

Figure S43. Composite of the NCI-60 dose response curves of the *Enterolobium gummiferum* stem bark hexane extract (BR 469/N192837).

N. *Plathymenia reticulata*

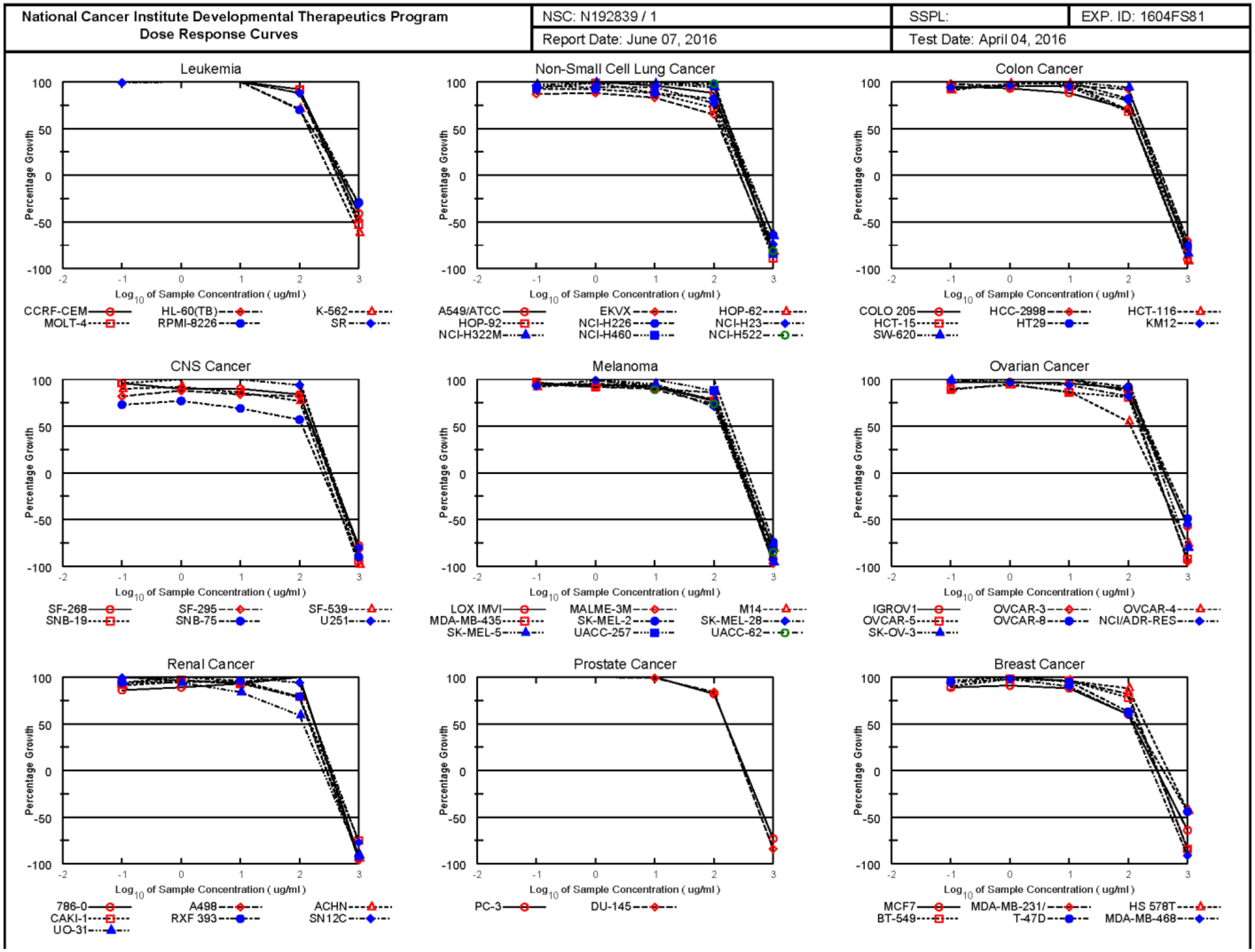

Figure S44. Dose response curves of the *Plathymenia reticulata* root wood hexan extract (BR 489/N192839) against 9 cell panels with different susceptibility.

National Cancer Institute Developmental Therapeutics Program Mean Graphs	NSC :N192839/1	Units :ug/ml	SSPL :	EXP. ID :1604FS81
	Report Date :June 07, 2016		Test Date :April 04, 2016	

Panel/Cell Line	Log_{10}GI50	Log_{10}TGI	Log_{10}LC50
**Leukemia**			
CCRF-CEM	2.32	2.69	> 3.00
HL-60(TB)	2.28	2.64	> 3.00
K-562	2.16	2.53	2.91
MOLT-4	2.29	2.64	2.98
RPMI-8226	2.20	2.71	> 3.00
SR	2.32	2.74	> 3.00
**Non-Small Cell Lung Cancer**			
A549/ATCC	2.23	2.52	2.82
EKVX	2.10	2.44	2.77
HOP-62	2.28	2.55	2.83
HOP-92	2.14	2.45	2.76
NCI-H226	2.22	2.56	2.90
NCI-H23	2.18	2.51	2.84
NCI-H322M	2.27	2.59	2.90
NCI-H460	2.25	2.53	2.81
NCI-H522	2.27	2.55	2.83
**Colon Cancer**			
COLO 205	2.13	2.44	2.75
HCC-2998	2.26	2.57	2.88
HCT-116	2.12	2.43	2.74
HCT-15	2.13	2.47	2.82
HT29	2.21	2.52	2.84
KM12	2.19	2.51	2.83
SW-620	2.25	2.53	2.81
**CNS Cancer**			
SF-268	2.21	2.52	2.83
SF-295	2.20	2.50	2.80
SF-539	2.15	2.44	2.72
SNB-19	2.29	2.54	2.78
SNB-75	2.05	2.39	2.73
U251	2.25	2.54	2.83
**Melanoma**			
LOX IMVI	2.16	2.46	2.75
MALME-3M	2.20	2.47	2.74
M14	2.18	2.49	2.80
MDA-MB-435	2.17	2.49	2.81
SK-MEL-2	2.33	2.60	2.87
SK-MEL-28	2.13	2.44	2.74
SK-MEL-5	2.16	2.44	2.73
UACC-257	2.22	2.52	2.82
UACC-62	2.15	2.46	2.78
**Ovarian Cancer**			
IGROV1	2.27	2.61	2.95
OVCAR-3	2.20	2.48	2.75
OVCAR-4	2.04	2.42	2.80
OVCAR-5	2.18	2.47	2.76
OVCAR-8	2.30	2.65	> 3.00
NCI/ADR-RES	2.23	2.60	2.96
SK-OV-3	2.29	2.56	2.84
**Renal Cancer**			
786-0	2.27	2.53	2.78
A498	2.28	2.53	2.77
ACHN	2.16	2.45	2.74
CAKI-1	2.19	2.51	2.84
RXF 393	2.17	2.46	2.76
SN12C	2.26	2.55	2.84
UO-31	2.06	2.39	2.73
**Prostate Cancer**			
PC-3	2.21	2.53	2.85
DU-145	2.20	2.50	2.80
**Breast Cancer**			
MCF7	2.08	2.49	2.89
MDA-MB-231/ATCC	2.19	2.49	2.80
HS 578T	2.29	2.67	> 3.00
BT-549	2.17	2.48	2.79
T-47D	2.12	2.59	> 3.00
MDA-MB-468	2.07	2.40	2.73
_MID	2.2	2.52	2.83
Delta	0.16	0.13	0.11
Range	0.29	0.35	0.28

Axis (GI50, TGI, LC50): +3 +2 +1 0 -1 -2 -3

Figure S45. Mean bar graph of the *Plathymenia reticulata* root wood hexane extract (BR 489/N192839) in the NCI-60 cell five-dose screen.

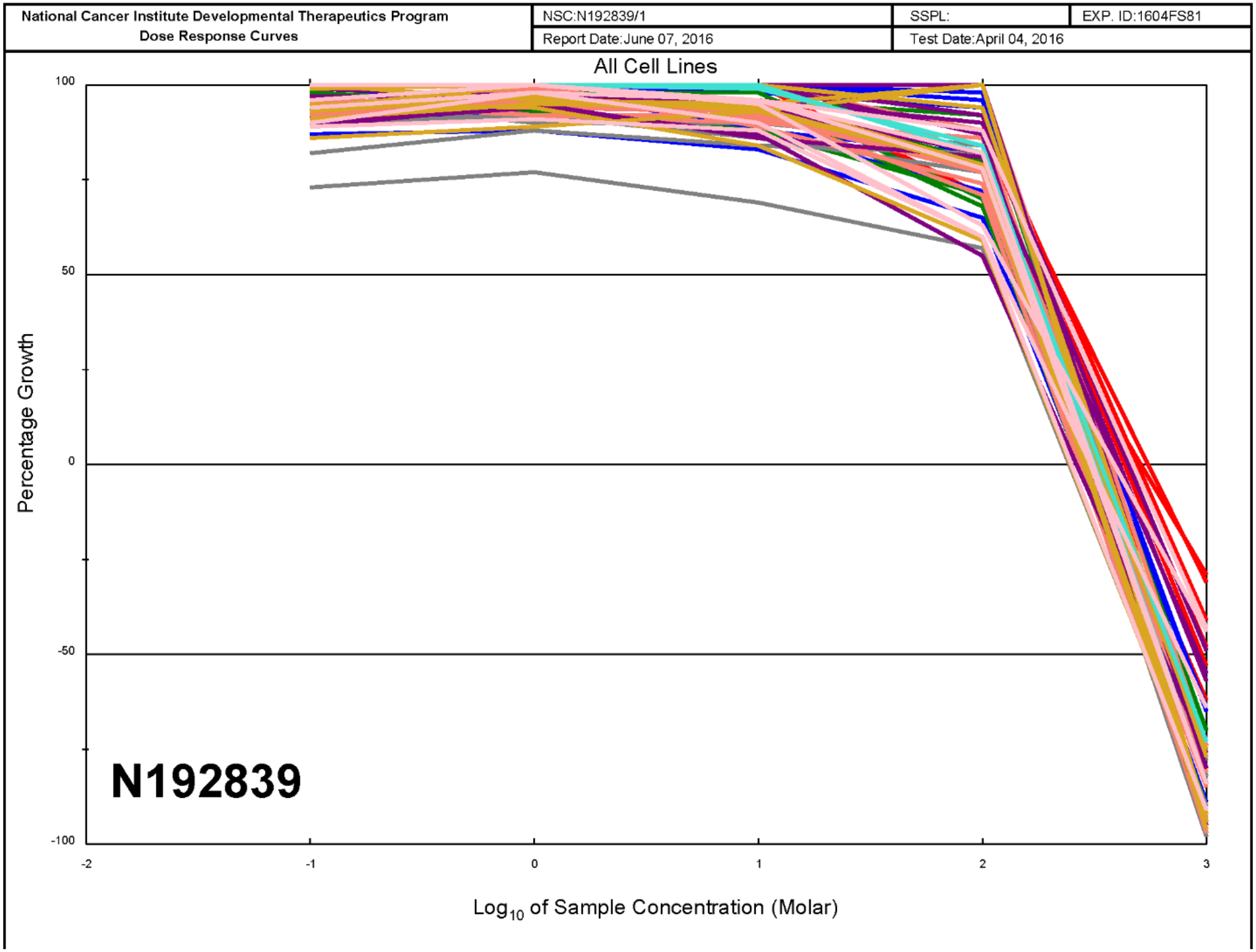

Figure S46. Composite of the NCI-60 dose response curves of the *Plathymenia reticulata* root wood hexane extract (BR 489/N192839).

O. *Psidium laruotteanum*

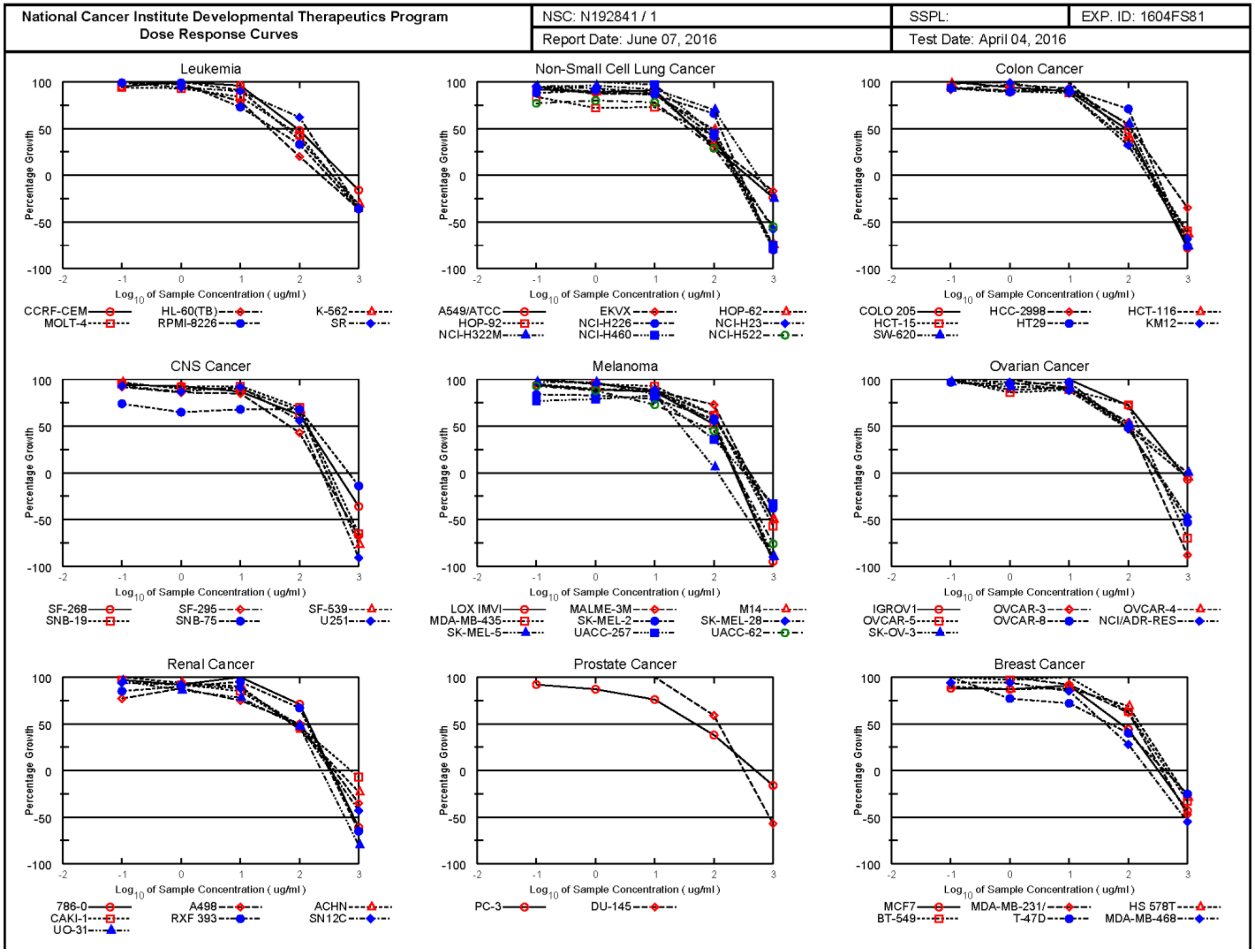

Figure S47. Dose response curves of the *Psidium laruotteanum* stem bark hexane extract (BR 549/N192841) against 9 cell panels with different susceptibility.

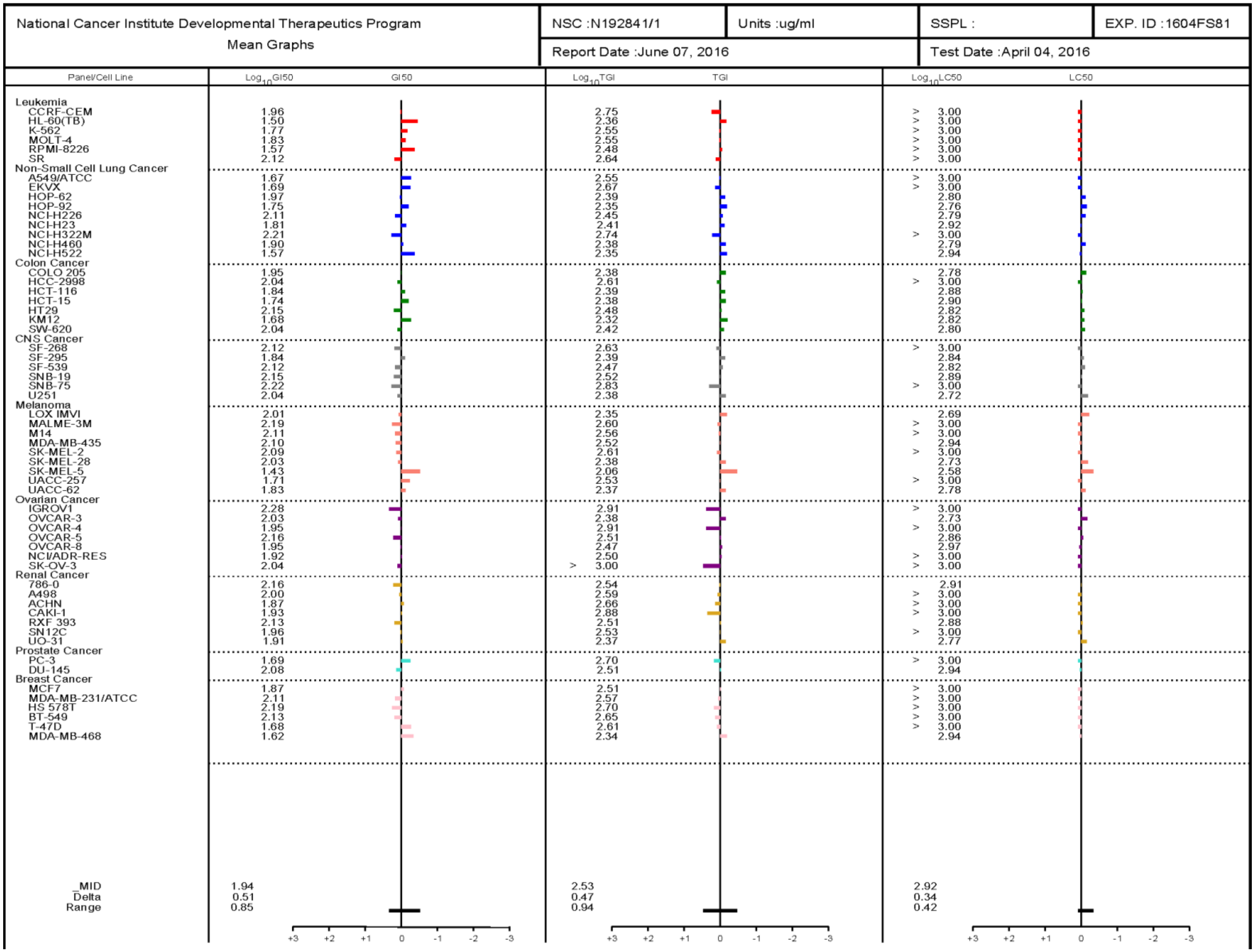

Figure S48. Mean bar graph of the *Psidium laruotteanum* stem bark hexane extract (BR 549/N192841) in the NCI-60 cell five-dose screen.

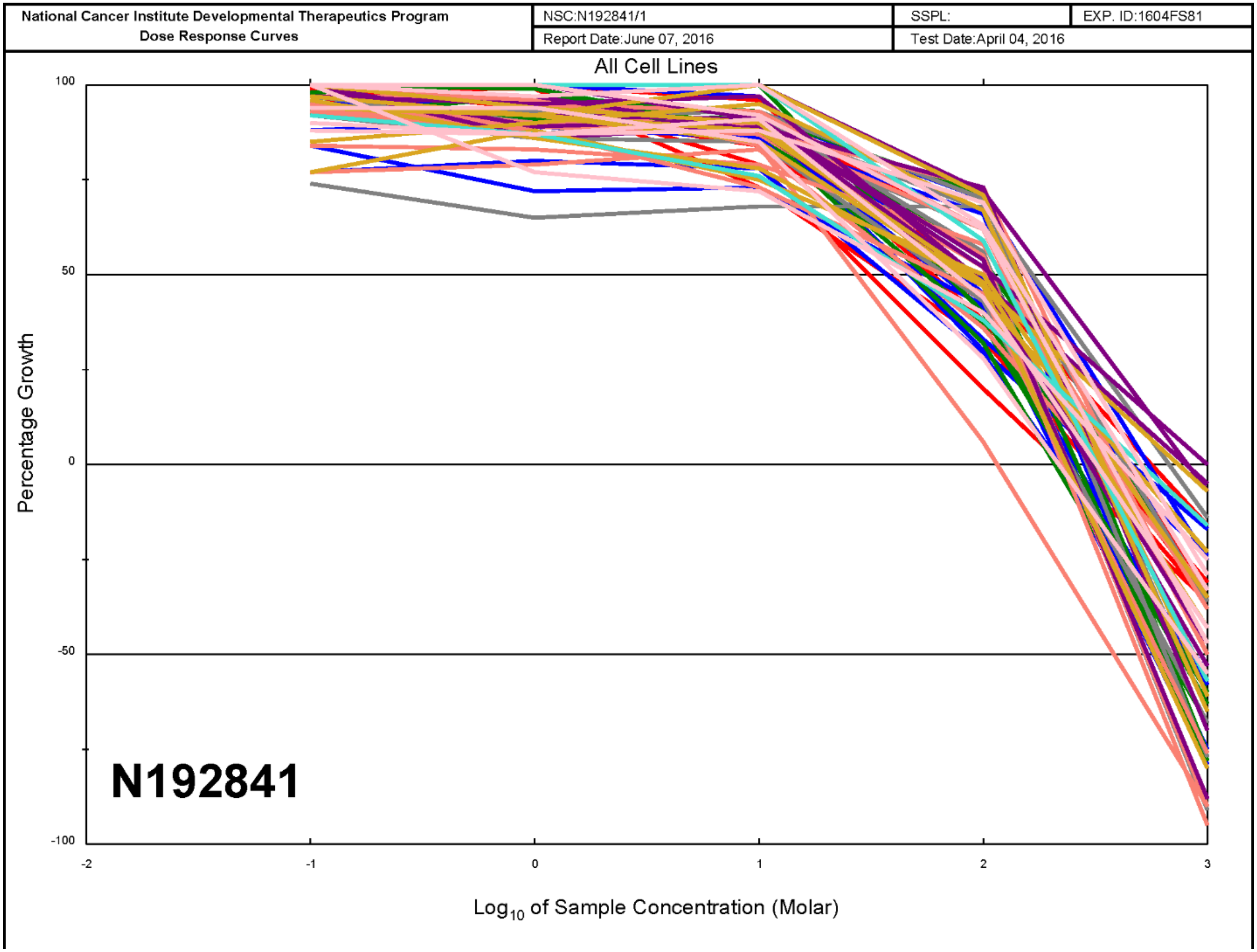

Figure S49. Composite of the NCI-60 dose response curves of the *Psidium laruotteanum* stem bark hexane extract (BR 549/N192841).

P. *Lippia rotundifolia*

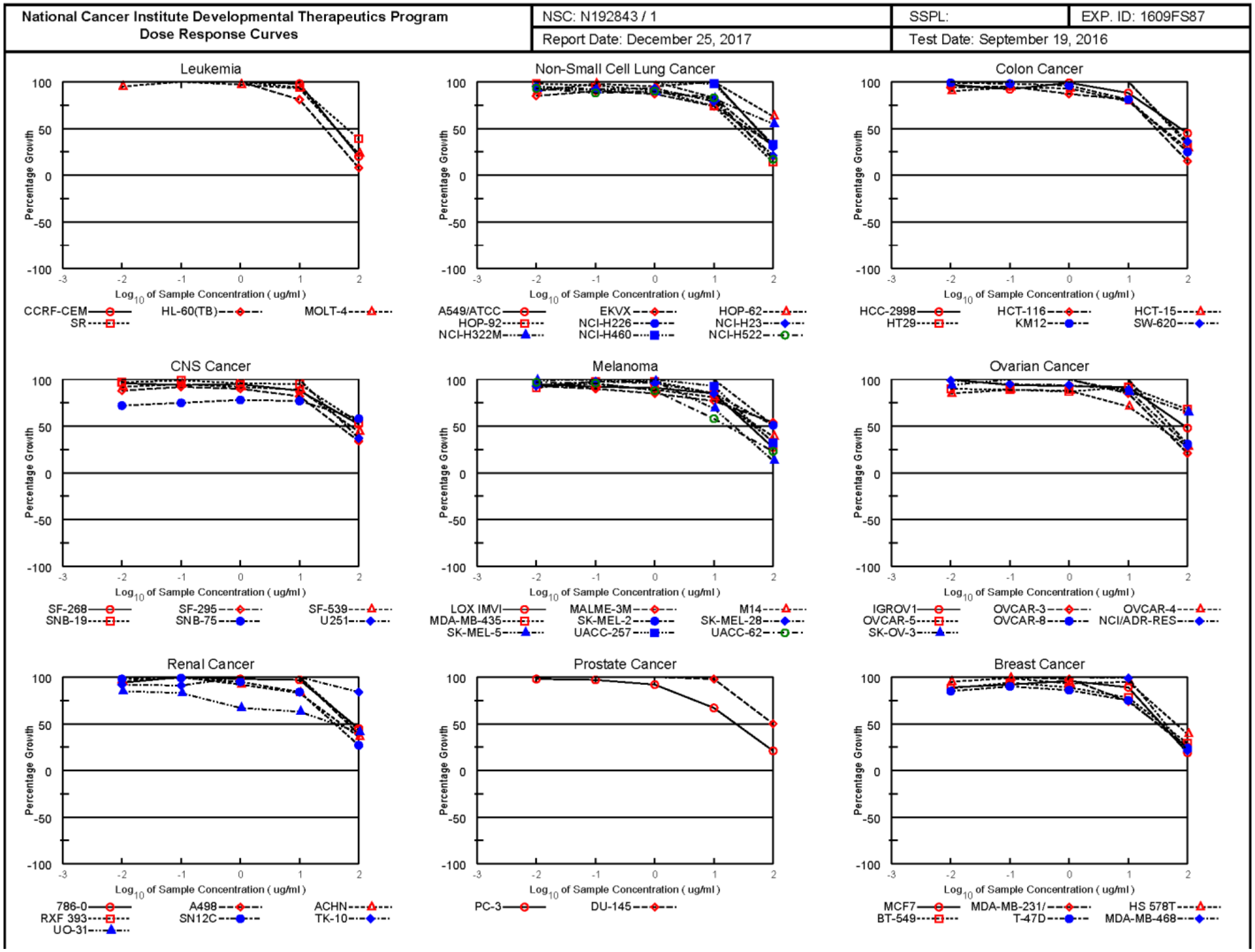

Figure S50. Dose response curves of the *Lippia rotundifolia* stem wood ethyl acetate extract (BR 660/N192843) against 9 cell panels with different susceptibility.

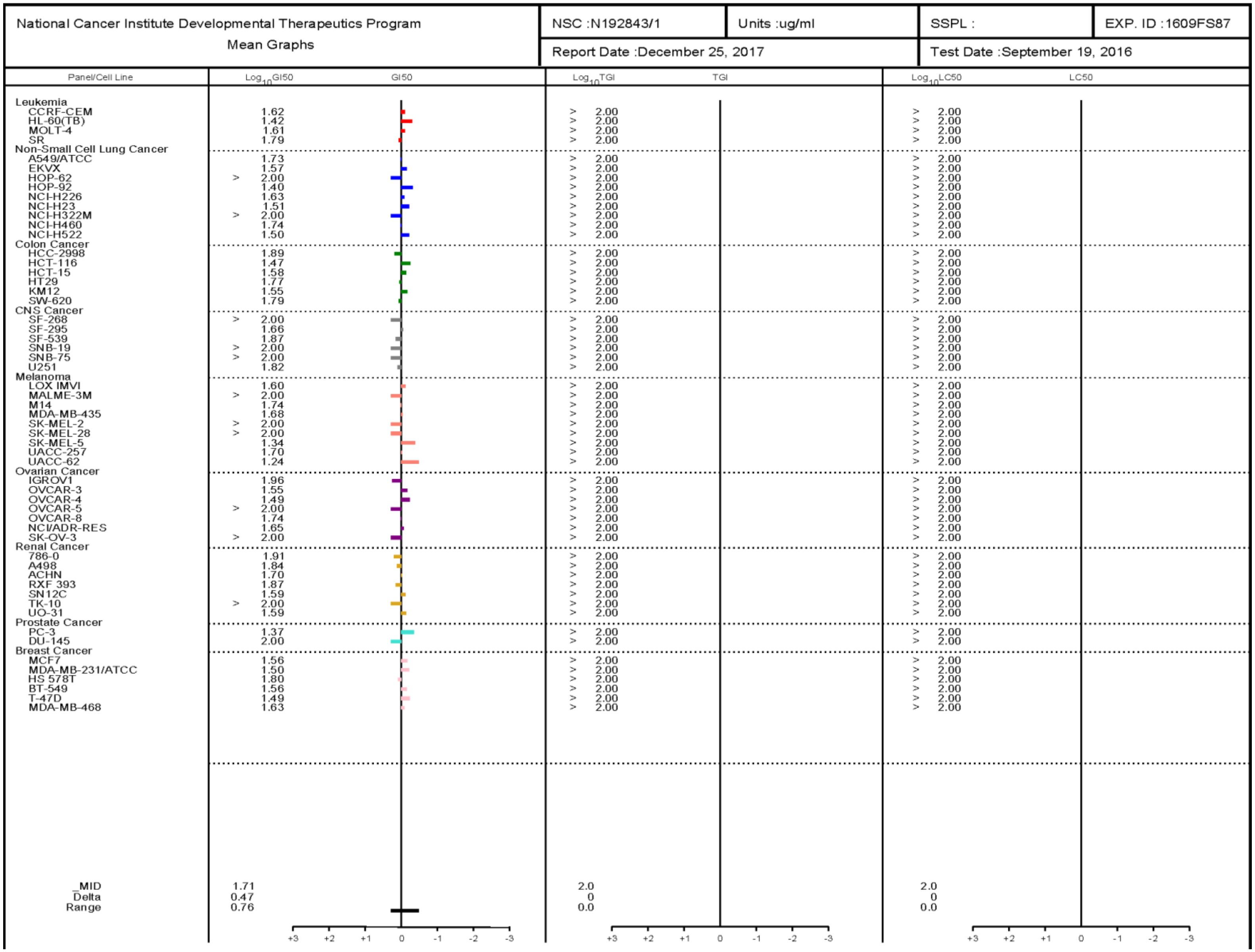

Figure S51. Mean bar graph of the *Lippia rotundifolia* stem wood ethyl acetate extract (BR 660/N192843) in the NCI-60 cell five-dose screen.

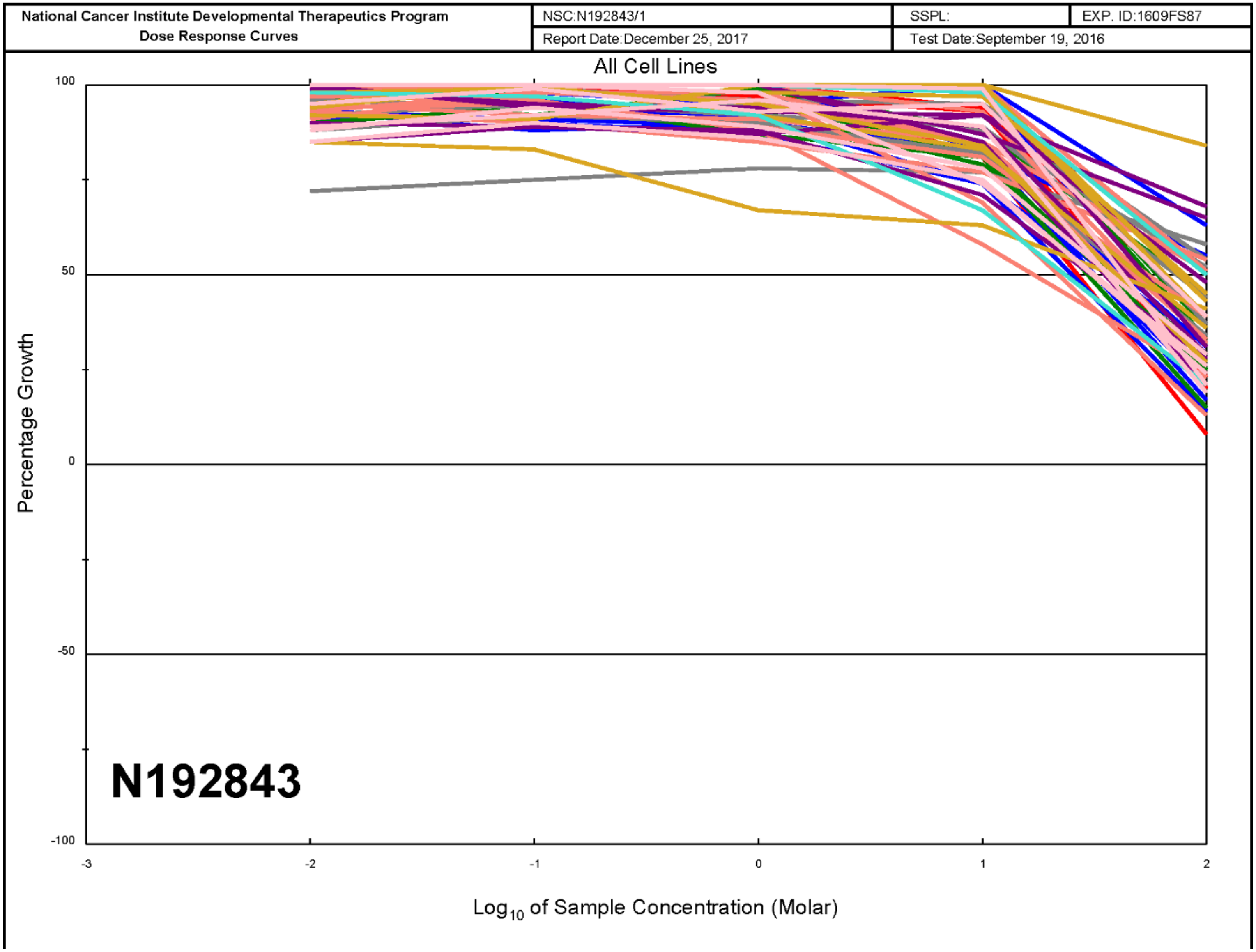

Figure S52. Composite of the NCI-60 dose response curves of the *Lippia rotundifolia* stem wood ethyl acetate extract (BR 660/N192843).

Q. *Connarus suberosus*

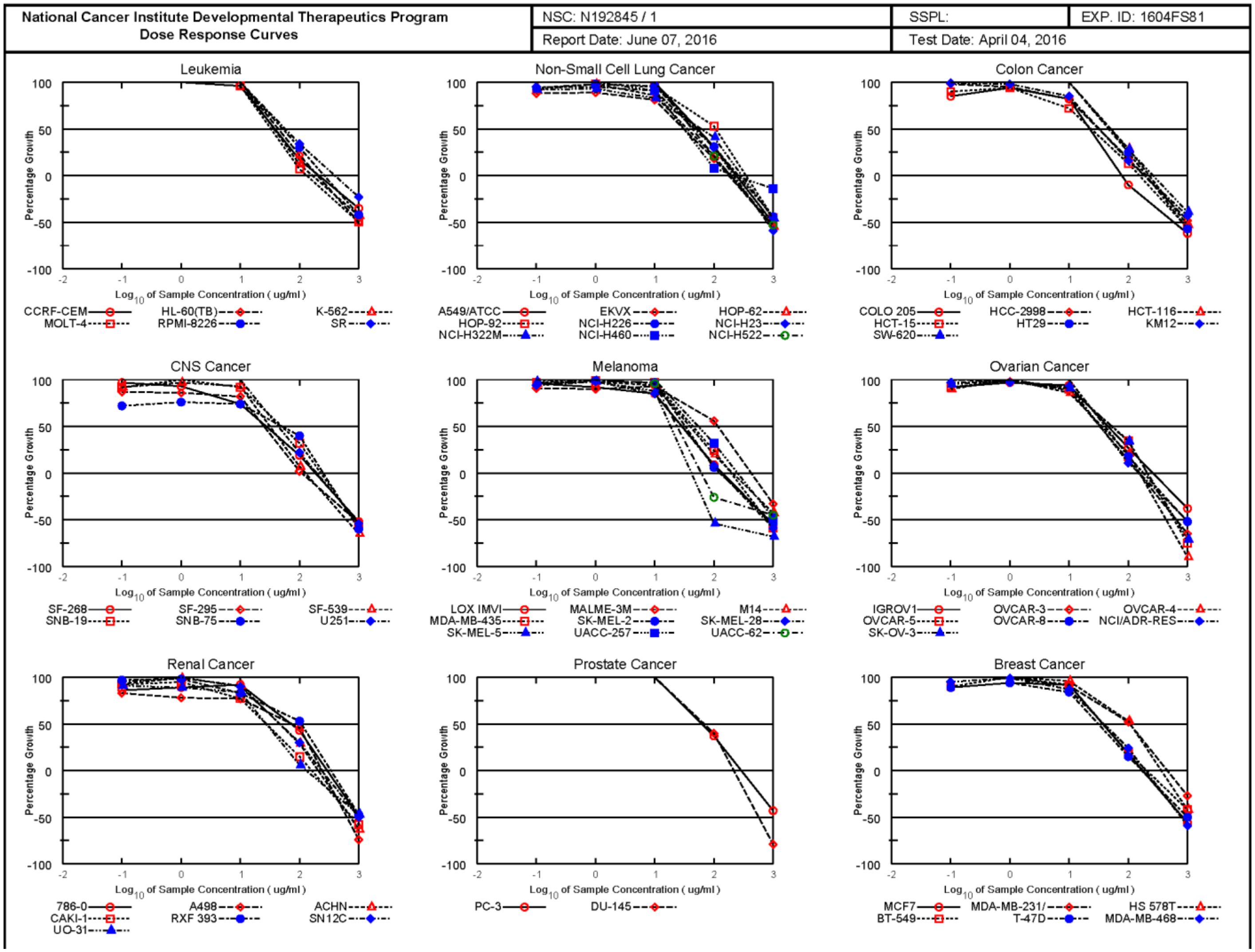

Figure S53. Dose response curves of the *Connarus suberosus* root wood ethyl acetate extract (BR 693/N192845) against 9 cell panels with different susceptibility.

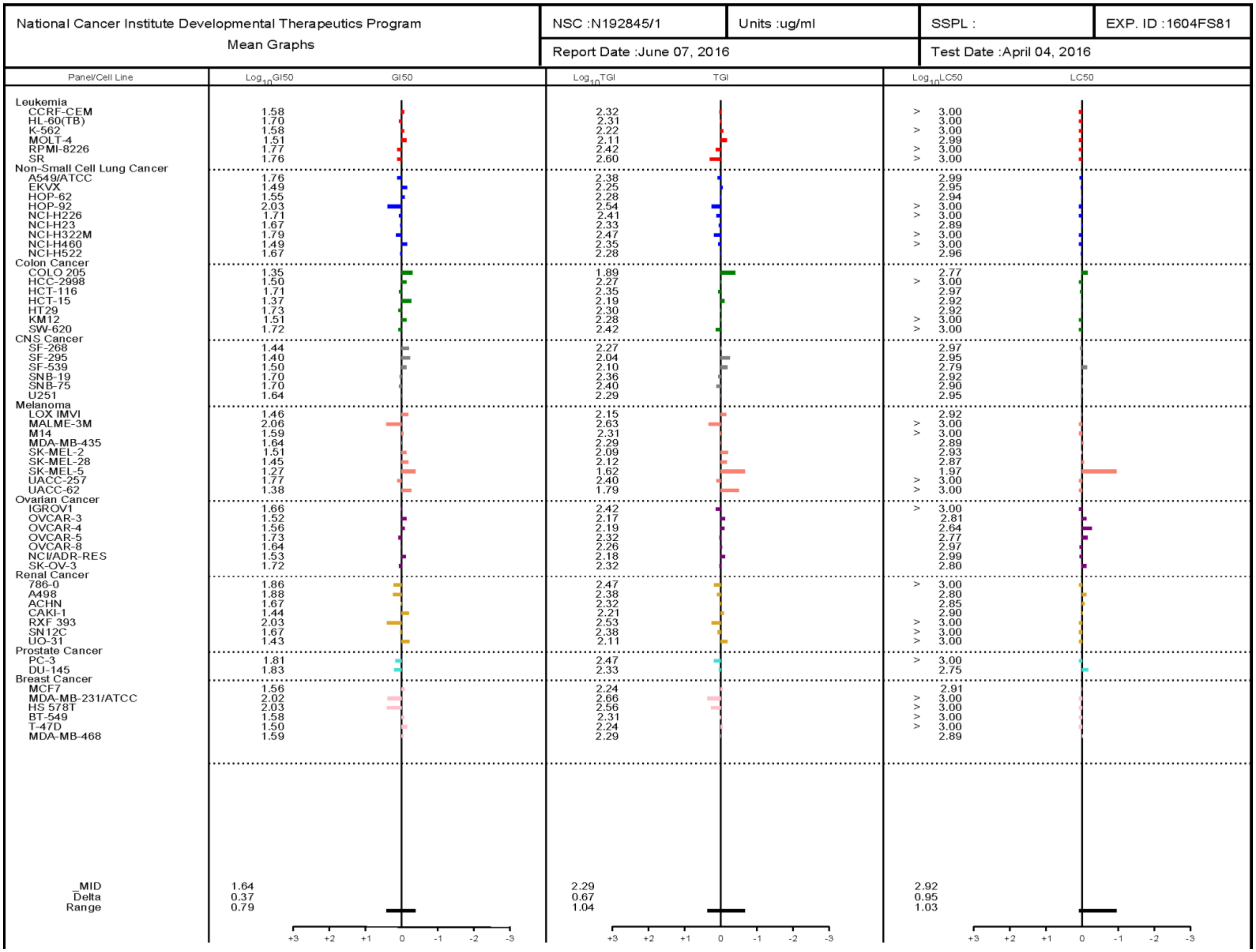

Figure S54. Mean bar graph of the *Connarus suberosus* root wood ethyl acetate extract (BR 693/N192845) in the NCI-60 cell five-dose screen.

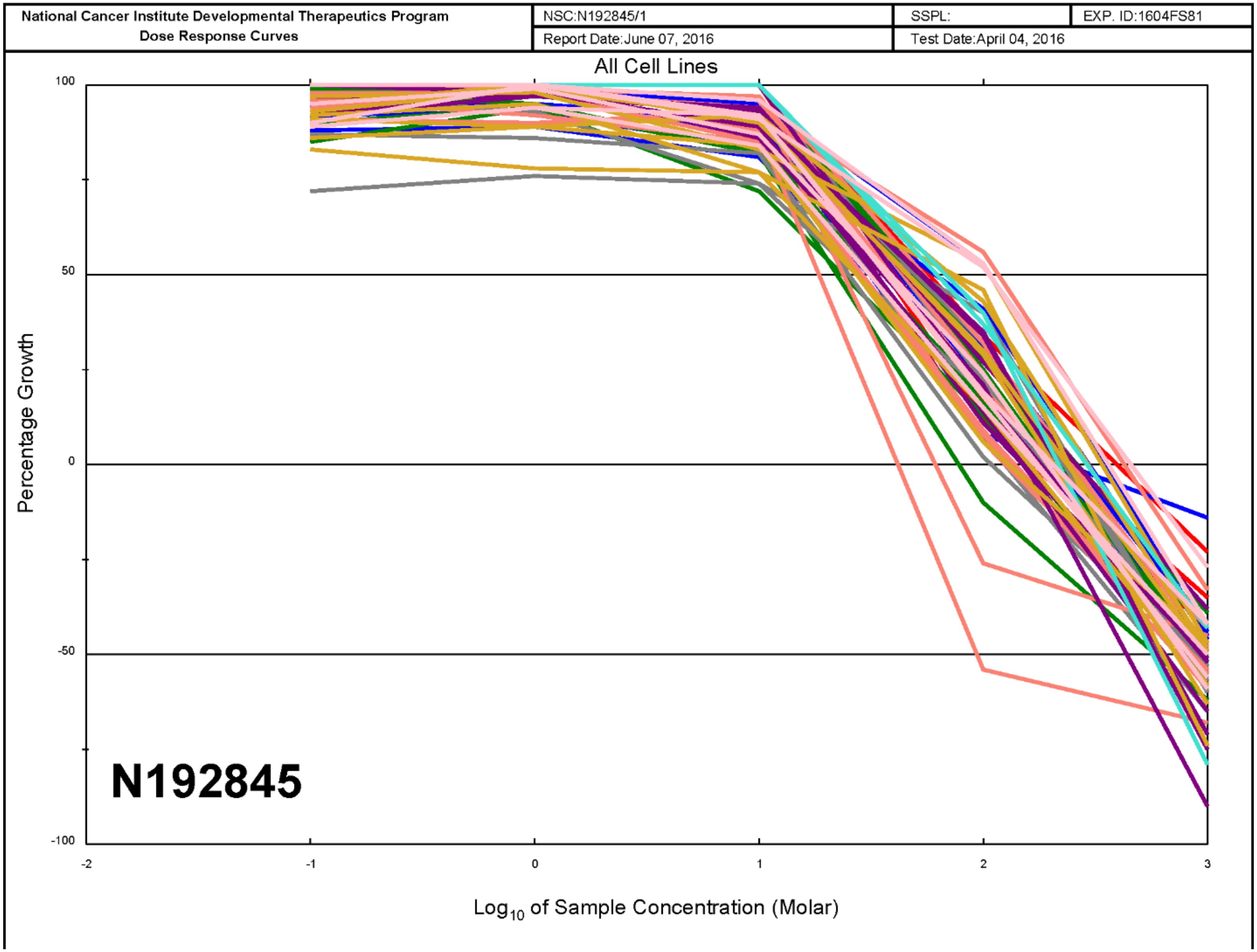

Figure S55. Composite of the NCI-60 dose response curves of the *Connarus suberosus* root wood ethyl acetate extract (BR 693/N192845).

References

[1] Silva TM, Dias MD, Pereira MT, Takahashi JA, Ferraz VP, Piló-Veloso D, Alcântra AFC. Effect of the γ-radiation on phenol fractions obtained from the leaves of *Echinodorus macrophyllus* Mich. Radiat Phys Chem 2012a; 81: 22–26

[2] Lorenzi H. Árvores brasileiras: Manual de identificação e cultivo de plantas arbóreas nativas do Brasil. Nova Odessa/SP: Instituto Plantarum; 2016

[3] Schmourlo G, Mendonça-Filho RR, Alviano CS, Costa SS. Screening of antifungal agents using ethanol precipitation and bioautography of medicinal and food plants. J Ethnopharmacol 2005; 96 (3): 563–568

[4] De Mesquita ML, Desrivot J, Bories C, Fournet A, De Paula JE, Grellier P, Espindola LS. Antileishmanial and trypanocidal activity of Brazilian Cerrado plants. Mem Inst Oswaldo Cruz 2005; 100 (7): 783–787

[5] Silva CR, Vieira PM, Santos SC, Chen-Chen L. Assessment of *Duguetia furfuracea* genotoxic and cytotoxic activity in bacteria and mice. An. Acad. Bras. Cienc. 2012b; 84 (1): 149–156

[6] Coelho AAM, de Paula JE, Espindola LS. Insecticidal activity of Cerrado plant extracts on *Rhodnius milesi* Carcavallo, Rocha, Galvão & Jurberg (Hemiptera: Reduviidae), under laboratory conditions. Neotrop Entomol. 2006a; 35 (1): 133–138

[7] Di Stasi LC, Oliveira GP, Carvalhaes MA, Queiroz-Junior M, Tien OS, Kakinami SH, Reis MS. Medicinal plants popularly used in the Brazilian Tropical Atlantic Forest. Fitoterapia 2002; 73: 69–91

[8] Corrêa MP, Penna LA. Dicionário das plantas úteis do Brasil e das exóticas cultivadas. Rio de Janeiro: Ministério da Agricultura, Instituto Brasileiro de Desenvolvimento Florestal; 1984

[9] Santos AKL. Estudo Fitoquímico de *Peschiera affinis* (Müell. Arg.) Miers [dissertation]. Fortaleza: Universidade Federal do Ceará; 2008

[10] Rodrigues VEG, Carvalho DA. Plantas medicinais no domínio dos cerrados. Lavras: Editora UFLA; 2001: 180p

[11] Brandão M, Laca-Buendía JP, Macedo JF. Árvores nativas e exóticas do Estado de Minas Gerais. Belo Horizonte: EPAMIG; 2002: 528p

[12] Silva AAR, Bezerra MM, Chaves HV, Pinto VPT, Franco ES, Vieira AM, Araújo EB, Cunha LR, Leite ACR, Maia MBS. Protective effect of *Chresta martii* extract on ethanol-induced gastropathy depends on alpha-2 adrenoceptors pathways but not on nitric oxide, prostaglandins or opioids. J Ethnopharmacol 2012c; 142: 206–212

[13] Lorenzi H, Matos FJA. Plantas medicinais do Brasil: nativas e exóticas. Nova Odessa/SP: Instituto Plantarum; 2008

[14] Felicio JD, Lins AP, Simoni IC, Gonçalves CR. Constituents of *Cybistax antisiphylitica*. Fitoterapia 1994; 65: 281–282

[15] Nunes GP, Silva MF, Resende UM, Siqueira JM. Plantas medicinais comercializadas por raizeiros no Centro de Campo Grande, Mato Grosso do Sul. Rev Bras Farmacogn 2003; 13: 83–95

[16] Mors WB, Rizzini CT, Pereira NA. Medicinal plants of Brazil. Algonac/Michigan: Reference Publications INC; 2000: 501p

[17] Souza FM. A contemporaneidade dos valores sociais, econômicos e culturais de produtos florestais não madeireiros para os produtores de Pirenópolis-GO [dissertation]. Brasília: Universidade de Brasília; 2016

[18] Audi EA, Otobone F, Martins JVC, Cortez DAG. Preliminary evaluation of *Kielmeyera coriacea* leaves extract on the central nervous system. Fitoterapia 2002; 73: 517–519

[19] Costa EA, Santos LR, Pontes IS, Matos LG, Silva GA, Lião LM. Analgesic and anti-inflammatory effects of *Cheiloclinium cognatum* root barks. Rev Bras Farmacogn 2007; 17 (4): 508–513

[20] Attuch IM. Conhecimentos tradicionais do Cerrado: sobre a memória de Dona Flor, raizeira e parteira [dissertation]. Brasília: Universidade de Brasília; 2006

[21] Quattrocchi U. CRC World Dictionary of Medicinal and Poisonous Plants: Common Names, Scientific Names, Eponyms, Synonyms, and Etymology. CRC Press Book; 2012

[22] Almeida SP, Proença CEB, Sano SM, Ribeiro JF. Cerrado: espécies vegetais úteis. Planaltina/Distrito Federal: EMBRAPA-CPAC; 1998

[23] Azevedo AO, Campos JJ, Galdino GS, Braga FC, Duarte IDG, Perez AC. Antinociceptive effect from *Davilla elliptica* hydroalcoholic extract. J Ethnopharmacol 2007; 113: 354–356

[24] Albernaz LC, de Paula JE, Romero GAS, Silva MRR, Grellier P, Mambu L, Espindola LS. Investigation of plant extracts in traditional medicine of the Brazilian Cerrado against protozoans and yeasts. J Ethnopharmacol 2010; 131 (1): 116–121

[25] Carvalho A. Popular Use, Chemical Composition and Trade of Cerrado's Medicinal Plants (Goiás, Brazil). Environment Development and Sustainabili 2004; 6: 307–316

[26] Grandtner MM, Chevrette J. Dictionary of trees: volume 2: South America: nomenclature, taxonomy and ecology. Amsterdam: Academic Press; 2013

[27] Souza LF. Recursos vegetais usados na medicina tradicional do Cerrado (comunidade de Baús, Acorizal, MT, Brasil). Rev Bras Pl Med 2007; 9 (4): 44–54

[28] Trentin DS, Giordani RB, Zimmer KR, da Silva AG, da Silva MV, Correia MT, Baumvol IJ, Macedo AK. Potential of medicinal plants from the Brazilian semi-arid region (Caatinga) against *Staphylococcus epidermidis* planktonic and biofilm lifestyles. J Ethnopharmacol 2011; 137 (1): 327–335

[29] Silva Júnior MCS. 100 Árvores do Cerrado: guia de Campo. Brasília: Rede de Sementes do Cerrado; 2005: 278p

[30] Farrapo NM, Silva GAA, Costa KN, Silva MG, Cogo JC, Dal Belo CA, Santos MG, Groppo FC, Oshima-Franco Y. Inhibition of *Bothrops jararacussu* venom activities by *Plathymenia reticulata* Benth extracts. J Venom Res 2011; 2: 52–58

[31] Santana DB, Da Costa RC, Araújo RM, De Paula JE, Silveira ER, Braz-Filho R, Espindola LS. Activity of Fabaceae species extracts against fungi and *Leishmania*: vatacarpan as a novel potent anti-Candida agente. Rev Bras Farmacogn 2015; 25: 401–406

[32] Silva SR, Silva, AP, Munhoz CB, Silva Jr MC, Medeiros MB. Guia de plantas do Cerrado utilizadas na Chapada dos Veadeiros. Brasília: WWF; 2001: 58p

[33] Agra M, de Freitas PF, Barbosa-Filho JM. Synopsis of the plants known as medicinal and poisonous in Northeast of Brazil. Rev Bras Farmacogn 2007; 17 (1): 114–140

[34] Rodrigues MS, Santos LS, Lopes Junior ML, Solano FAR, Tavares JL, Ripardo Filho HS, Arruda MSP, Guilhon GMSP, Souza Filho APS. Atividade alelopática das folhas de *Myrcia linearifolia*. Congresso Brasileiro de Química, Rio de Janeiro; 2008

[35] Barroso GM. Sistemática de Angiosperma do Brasil. Viçosa/MG: UFV; 1986: 130p

[36] Vencato I, Silva FM, Oliveira CMA, Kato L, Tanaka CMA, Silva CC, Sabino JR Vallesiachotamine. Acta Cryst E 2004; 62: 429–430

[37] Almeida MG, Rigonato VD. As fitofisionomias e a interrelação das populações tradicionais com o bioma Cerrado. Programa Centro Oeste de Pesquisa e Pós-Graduação - POCPG/CNPq: Relatório de pesquisa; 2001

[38] Coelho VPM, Agra MF, Barbosa MRV. Estudo farmacobotânico das folhas de *Tocoyena formosa* (Cham. & Schltdl.) K. Schum. (Rubiaceae). Rev. Bras. Farmacogn 2006b; 16 (2): 170–177

[39] de la Cruz MGF. Plantas medicinais utilizadas por raizeiros. Uma abordagem etnobotânica no contexto da saúde e da doença [dissertation]. Cuiabá: Universidade Federal de Mato Grosso; 1997

[40] Da Silva SL, Figueiredo PMS, Yano T. Chemotherapeutic potential of the volatile oils from *Zanthoxylum rhoifolium* Lam. leaves. Eur J Pharmacol 2007; 576: 180–188

[41] Chaves EMF, Barros RFM. Diversidade e uso de recursos medicinais do carrasco na APA da Serra da

Ibiapaba, Piauí, Nordeste do Brasil. Rev. Bras. Pl. Med. 2012; 14 (3): 476–486
[42] Rodrigues E. Plants of restricted use indicated by three cultures in Brazil (Caboclo-river dweller, Indian and Quilombola). J Ethnopharmacol 2007; 111 (2): 295–302
[43] Mendes FR, Carlini EA. Brazilian plants as possible adaptogens: an ethnopharmacological survey of books edited in Brazil. J Ethnopharmacol 2007; 109: 493–500
[44] Valentini CMA, Almeida JD, Coelho MFB, Ortíz CER. Uso de *Siparuna guianensis* Aublet (negramina) em Bom Sucesso, município de Várzea Grande, Mato Grosso. Revista de Biologia Neotropical 2008; 5: 11–22
[45] Hirschmann GS, de Arias AR. A survey of medicinal plants of Minas Gerail. Brazil. J Ethnopharmacol 1990; 29 (2): 159–172
[46] Meira MR. Ecogeografia e Diversidades Genéticas e Químicas de *Lippia aff. rotundifolia* Cham [dissertation]. Lavras: Universidade Federal de Lavras; 2016
[47] Nasser ALM, Mazzolin LP, Hiruma-Lima A, Santos LS, Eberlin MN, Souza Brito ARM, Vilegas W. Preparative Droplet Counter-Current Chromatography for the Separation of the New Nor-Seco-Triterpene and Pentacyclic Triterpenoids from *Qualea parviflora*. Chromatographia 2006; 64: 695–699
[48] Coelho FBR. Levantamento etnofarmacológico realizado na comunidade mumbuca localizada no Jalapão – TO. Rev. Eletronica Farm 2005; 2 (2): 52–55
[49] Zhou B, Baj NJ, Glass TE, Malone S, Werkhoven MCM, Troon FV, Wisse JH, Kingston DGI. Bioactive labdane diterpenoids from *Renealmia alpinia* collected in the Suriname Rainforest. J Nat Prod 1997; 60 (12): 1287–1293
[50] Zhang J.-H., Chung T.D.Y., Oldenburg K.R. A Simple Statistical Parameter for Use in Evaluation and Validation of High Throughput Screening Assays. J Biomolecular Screening 4: 67-73, 1999.
